# A Great Barrier Reef *Sinularia* sp. Yields Two New Cytotoxic Diterpenes

**DOI:** 10.3390/md10081619

**Published:** 2012-07-31

**Authors:** Anthony D. Wright, Jonathan L. Nielson, Dianne M. Tapiolas, Catherine H. Liptrot, Cherie A. Motti

**Affiliations:** Australian Institute of Marine Science, PMB no. 3, Townsville MC, Townsville, QLD 4810, Australia; Email: adwright@hawaii.edu (A.D.W.); jonathon.nielson@acdlabs.com (J.L.N.); d.tapiolas@aims.gov.au (D.M.T.); catherine.liptrot@jcu.edu.au (C.H.L.)

**Keywords:** *Sinularia*, Alcyoniidae, anticancer activity, lobane, cembrane, diterpene

## Abstract

The methanol extract of a *Sinularia* sp., collected from Bowden Reef, Queensland, Australia, yielded ten natural products. These included the new nitrogenous diterpene (4R*,5R*,9S*,10R*,11*Z*)-4-methoxy-9-((dimethylamino)-methyl)-12,15-epoxy-11(13)-en-decahydronaphthalen-16-ol (**1**), and the new lobane, (1R*,2R*,4S*,15*E*)-loba-8,10,13(14),15(16)-tetraen-17,18-diol-17-acetate (**2**). Also isolated were two known cembranes, sarcophytol-B and (1*E*,3*E*,7*E*)-11,12-epoxycembratrien-15-ol, and six known lobanes, loba-8,10,13(15)-triene-16,17,18-triol, 14,18-epoxyloba-8,10,13(15)-trien-17-ol, lobatrientriol, lobatrienolide, 14,17-epoxyloba-8,10,13(15)-trien-18-ol-18-acetate and (17*R*)-loba-8,10,13(15)-trien-17,18-diol. Structures of the new compounds were elucidated through interpretation of spectra obtained after extensive NMR and MS investigations and comparison with literature values. The tumour cell growth inhibition potential of **1** and **2** along with loba-8,10,13(15)-triene-16,17,18-triol, 14,17-epoxyloba-8,10,13(15)-trien-18-ol-18-acetate, lobatrienolide, (1*E*,3*E*,7*E*)-11,12-epoxycembratrien-15-ol and sarcophytol-B were assessed against three human tumour cell lines (SF-268, MCF-7 and H460). The lobanes and cembranes tested demonstrated 50% growth inhibition in the range 6.8–18.5 µM, with no selectivity, whilst **1** was less active (GI_50_ 70–175 µM).

## 1. Introduction

There have been many reports documenting the diversity of secondary metabolites produced by soft corals from the genus *Sinularia*, including sesquiterpenes [1,2], diterpenes [3,4,5,6,7], cembranoids [8,9,10,11], polyhydroxylated steroids [12], glycosides [13], sphingosines [14], farnesyl quinols [15,16], and polyamines [17]. These metabolites have been shown to possess a range of biological activities including antimicrobial [5], antiviral [4], anti-inflammatory [4,11], cytotoxic [8,9,10,17], anticancer [3,18], antifouling [19], antifeedant [20], and allelopathic [21,22] activities. Given this wide-ranging diversity in chemical structure and biological activity, it is not surprising that soft corals, which do not have a hard calcareous skeleton, are relatively well defended against predation [20] and are effective competitors for space on coral reefs [21]. As a result, the *Sinularia* genus remains an attractive target for the discovery of novel bioactive metabolites.

As part of the biodiscovery program at the Australian Institute of Marine Science (AIMS), the ethanol (EtOH) extract of a Great Barrier Reef soft coral *Sinularia* sp., was determined to have significant activity in the NCI 60 cell line COMPARE analysis [23]. Based on this, the sample was selected for recollection, large scale extraction and workup. The methanol (MeOH) extract of the recollected soft coral tissue was subjected to bioassay-guided fractionation, using C18 flash vacuum liquid chromatography and preparative C18 HPLC, to yield the new nitrogenous diterpene (4R*,5R*,9S*,10R*,11*Z*)-4-methoxy-9-((dimethylamino)-methyl)-12,15-epoxy-11(13)-en-decahydronaphthalen-16-ol (**1**), the new lobane, (1R*,2R*,4S*,15*E*)-loba-8,10,13(14),15(16)-tetraen-17,18-diol-17-acetate (**2**), and eight known diterpenes: two cembranes, sarcophytol-B [24] and (1*E*,3*E*,7*E*)-11,12-epoxycembratrien-15-ol [8], and six known lobanes, loba-8,10,13(15)-triene-16,17,18-triol [25], 14,18-epoxyloba-8,10,13(15)-trien-17-ol [26], lobatrientriol [7], lobatrienolide [7], 14,17-epoxyloba-8,10,13(15)-trien-18-ol-18-acetate [26] and (17*R*)-loba-8,10,13(15)-trien-17,18-diol [27]. The structural elucidation and biological activities of **1**, **2** and of the known compounds loba-8,10,13(15)-triene-16,17,18-triol, 14,17-epoxyloba-8,10,13(15)-trien-18-ol-18-acetate, lobatrienolide, (1*E*,3*E*,7*E*)-11,12-epoxycembratrien-15-ol and sarcophytol-B against a panel of human tumour cell lines are also presented.

## 2. Results and Discussion

The ^13^C NMR and ESI-FTMS of **1** established its molecular formula to be C_24_H_43_O_3_N, requiring four degrees of unsaturation. The ^1^H and ^13^C NMR spectral data of **1** (Table 1) showed the molecule to contain a trisubstituted double-bond (δ_C_ 142.5, s, C-11; 117.8, d, C-13; δ_H_ 5.59, d, 5.2, H-13) as the only multiple bond within the molecule and accounted for one of the degrees of unsaturation. This information, in combination with the molecular formula, showed the molecule to be tricyclic.

**Table 1:** ^1^H and ^13^C NMR data (300 MHz and 75 MHz, CD_3_OD) for (4R*,5R*,9S*,10R*,11*Z*)-4-methoxy-9-((dimethylamino)-methyl)-12,15-epoxy-11(13)-en-decahydronaphthalen-16-ol (**1**).

No.	13C δ (m)	1H δ (m, J Hz)	COSY	gHMBC	nOe
1	42.3 (t)	1.84 (1H, m)	H_b_-1, H_b_-2	C-10, C-5, C-9, C-2, C-3	
		1.24 (1H, m)	H_a_-1, H_a_-2, H_b_-2	C-5, C-19, C-2	
2	28.1 (t)	1.59 (1H, m )	H_a_-1, H_a_-3	C-4	
		1.45 (1H, m)	H_a_-1	C-10, C-1, C-4	
3	36.3 (t)	1.85 (1H, m)	H_a_-2, H_b_-3	C-5, C-1, C-4, C-20	
		1.60 (1H, m)	H_b_-2, H_a_-3	C-5, C-1, C-4	
4	76.8 (s)				
5	53.1 (d)	1.49 (1H, m)	H_2_-6	C-6, C-19, C-4, C-20	
6	26.7 (t)	1.82 (2H, m)	H-5, H-7	C-10, C-8	
7	43.1 (d)	1.86(1H, m)	H_2_-6	C-5, C-8, C-9	
8	25.0 (t)	1.85 (1H, m)	H_b_-8, H-9		
		1.52 (1H, m)	H_a_-8	C-10, C-7, C-22	
9	45.2 (d)	1.52 (1H, m)	H_a_-22, H_b_-22	C-10, C-7, C-22	
10	38.0 (s)				
11	142.5 (s)				
12	69.0 (t)	4.16 (2H, brs)	H-13	C-7, C-11, C-13, C-15	
13	117.8(d)	5.59 (1H, d, 5.2)	H-12, H_a_-14, H_b_-14	C-7, C-12, C-14, C-15	
14	26.3 (t)	2.12 (1H, m)	H-13, H_b_-14, H-15		
		2.00 (1H, m)	H-13, H_a_-14, H-15		
15	81.9 (d)	3.25 (1H, m)	H_a_-14, H_b_-14	C-17, C-18	
16	72.7 (s)				
17	25.2 (q)	1.17 (3H, s)		C-15, C-16, C-18	
18	25.6 (q)	1.17 (3H, s)		C-15, C-16, C-17	
19	15.2 (q)	0.89 (3H, s)			H_b_-1, H_b_-2, H-20, H_2_-22
20	18.7 (q)	1.08 (3H, s)		C-5, C-4, C-3	H_b_-1, H_b_-2, H-19
21	48.1 (q)	3.16 (3H, s)		C-4	H-5
22	60.5 (t)	3.25 (1H, dd, 3.2, 11.0)	H-9, H_b_-22	C-10, C-8, C-9	
		2.93 (1H, dd, 11.0, 13.1)	H-9, H_a_-22	C-9	
23	45.2 (q)	2.90 (3H, s)		C-22, C-24	
24	45.2 (q)	2.90 (3H, s)		C-22, C-23	

From the ^1^H-^1^H COSY spectrum of **1** spin systems from H_b_-1 (δ_H_ 1.24, m) to H_2_-3 (δ_H_ 1.85, m; 1.60, m) via H_a/b_-2 (δ_H_ 1.59, m; 1.45, m) and from H-5 (δ_H_ 1.49, m) to H_2_-22 (δ_H_ 3.25, dd, 3.2, 11.0; 2.93, dd, 11.0, 13.1) via H_2_-6 (δ_H_ 1.82, m), H-7 (δ_H_ 1.86 m), H_2_-8 (δ_H_ 1.85, m; 1.52 m) and H-9 (δ_H_ 1.52, m) could be discerned. This information together with cross-peaks in the HMBC spectrum from H_b_-1 and H-5 to C-19 (δ_C_ 15.2, q), from H-20 (δ_H_ 1.08, s) to C-3, C-4 and C-5, and from H-7 to the olefinic C-11 (δ_C_ 142.5, s), showed the presence of a substituted bicyclic ring system.

The ^1^H-NMR spectrum of **1** displayed singlet resonances of a methoxy (-OCH_3_) at δ_H_ 3.16 and an *N*,*N*-dimethyl substituted tertiary amine (-N(CH_3_)_2_) at δ_H_ 2.90. The HMBC correlation from δ_H_ 3.16 (s) to δ_C_ 76.8 (s, C-4) located the methoxy at C-4 while correlations from both H-23/24 to and δ_C_ 60.5 (C-22) located the tertiary amine at C-22.

**Scheme 1:**
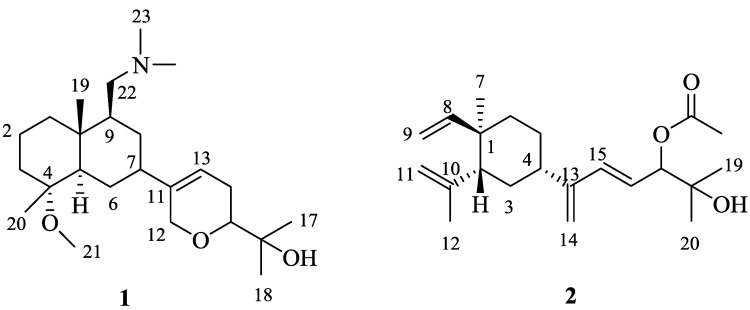
Structures of (4R*,5R*,9S*,10R*,11*Z*)-4-methoxy-9-((dimethylamino)-methyl)-12,15-epoxy-11(13)-en-decahydronaphthalen-16-ol (**1**), and (1R*,2R*,4S*,15*E*)-loba-8,10,13(14),15(16)-tetraen-17,18-diol-17-acetate (**2**).

Further analysis of the ^1^H-^1^H COSY indicated the presence of a spin system from δ_H_ 5.59 (d, 5.2, H-13) to δ_H_ 3.25 (m, H-15) via δ_H_ 2.12 and 2.00 (m, H_2_-14). HMBC correlations from H-13 to C-7 located the C-11 side chain at C-7 of the bicyclic ring system. Based on HMBC correlations from H_3_-17/H_3_-18 to δ_C_ 72.7 (C-16), the two methyl groups with resonances at δ_H_ 1.17 (H-17/18) were connected to a tertiary carbon bearing an OH, forming a propan-2-ol-2-yl moiety [28]. The data so far accounted for three of the four oxygens, the double-bond, two of the rings, and the nitrogen, leaving one oxygen and one ring unassigned. An ether linkage, forming the third ring, was deduced between C-12 and C-15 based on the HMBC correlation from δ_H_ 4.16 (brs, H-12) to δ_C_ 81.9 (C-15), and was further supported by a C-O-C stretch at 1080 cm^−1^ in the IR spectrum of **1**. Hence the planar structure of **1**, a diterpene, is best described as (4R*,5R*,9S*,10R*,11*Z*)-4-methoxy-9-((dimethylamino)-methyl)-12,15-epoxy-11(13)-en-decahydronaphthalen-16-ol (Scheme 1).

**Figure 1:**
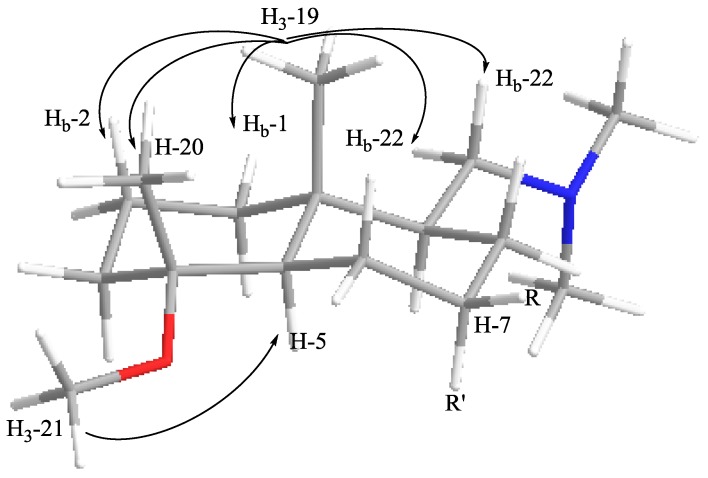
Diagnositic nOe correlations for partial structure of **1**.

The nOe data of **1** showed correlations between H_3_-19 (δ_H_ 0.89, s) and H_b_-1, H_b_-2, H_3_-20 and H_b_-22. These cross peaks revealed the two fused six-membered rings to have a trans-ring junction, CH_3_-19 and CH_3_-20 to be axial and therefore on the same side of **1**, and the side-chain at C-9 to be on the same side as C-19 (Figure 1). Furthermore, nOe correlations were observed between H_3_-21 and H-5 indicating they were on the same side of the molecule as each other but the opposite side of C-19 (Figure 1). The configurations at C-7 and C-15 remain unresolved. Based on the above findings, the relative configurations of chiral carbons C-10, C-5, C-9 and C-4 of **1** were assigned as 4R*, 5R*, 9S* and 10R* (Scheme 1).

The ^13^C NMR and ESI-FTMS of **2** established its molecular formula as C_20_H_34_O_3_, indicating the molecule to have four degrees of unsaturation. The ^1^H and ^13^C NMR spectral data of **2** (Table 2) showed it contained a vinyl moiety (δ_C_ 151.6, d, C-8; 110.4, t, C-9; δ_H_ 5.87, dd, 10.8, 17.6 Hz, H-8; δ_H_ 4.93, dd, 1.2, 17.6 Hz, H_a_-9; δ_H_ 4.90, dd, 1.2, 10.8 Hz, H_b_-9), an isopropenyl group (δ_C_ 148.9, s, C-10; 112.7, t, C-11, 25.3, q, C-12; δ_H_ 4.81, brt, 1.5 Hz, H_a_-11; 4.59, brs, H_b_-11; 1.71, brs, H_3_-12) and a tertiary methyl group (δ_C_ 17.1, q, C-7; δ_H_ 1.03, s, H_3_-7), characteristic of the 3-isopropenyl-4-methyl-4-vinylcyclohexane-1-yl moiety found in lobane-type diterpenoids [7,25,26,27]. This partial structure was confirmed by analysis of the COSY, HMQC, and HMBC NMR spectral data of **2**. The relative configuration about C-1 and C-2 in **2** was found to be the same as in elemol and several other lobanes, as established by comparison of the ^1^H and ^13^C NMR data for each molecule at centres C-1, C-2, C-6, C-7, C-8, C-9, C-10, C-11 and C-12 [25,27]. Also evident from this data was an exo-methylene (δ*_C_* 151.9, s, C-13; 114.3, t, C-14; δ_H_ 5.06, s, H_2_-14), an endo-disubstituted double-bond (δ*_C_* 137.4, d,C-15; 124.4, d, C-16; δ_H_ 6.26, d, 16.0, H-15; 5.78, dd, 7.6, 16.0, H-16), a carbonyl group in the form of an acetate (δ_C_ 172.1, s, O-CO-CH_3_; 21.1, q; δ_H_ 2.09, s, O-CO-CH_3_), a CH bearing the acetate (δ_C_ 82.2, d, C-17; δ_H_ 5.14, brd, 7.6, H-17), and a propan-2-ol-2-yl moiety the same as that found in **1**. These assignments were corroborated by the IR data with terminal vinyl C-H stretches at 3079 and 3012 cm^−1^, a carbonyl ester band at 1734 cm^−1^, and an alcohol OH stretch at 3084 cm^−1^. This data accounted for all of the remaining unsaturation within the molecule as well as the previously unaccounted for C_10_H_15_O_3_. From the HMBC data of **2** (Table 2), it was evident that C-17 bonded to both C-15 and C-18, as well as the oxygen of the acetyl function. Further, HMBC correlations between H-14 and the carbons C-4, C-13 and C-15, confirmed the side-chain to be attached at C-4 and that the two double-bonds were conjugated, an observation supported by the UV maxima of **2** at 227 nm. With the planar structure of **2** deduced, the double-bond geometry and stereochemistry required resolution. The magnitude of the coupling constant between H-15 and H-16 (*J* = 16.0 Hz), showed Δ^15^ to have *E* geometry. The relative configurations at C-1 and C-2 were confirmed to be the same as in the known lobane loba-8,10,13(15)-triene-16,17,18-triol [25] on the basis of comparable ^13^C NMR chemical shift for the same centres. The relative configurations at C-1, C-2 and C-4 were assigned based on NOESY NMR correlations from H-4 to H-2, H_2_-5, H_a_-6, H-14, H-15, H-16, H_3_-19, H_3_-20 and O-CO-CH_3_, and from H-12 to H-2, H_a_-6, H-7, H-8, H_a_-9, H_2_-11, H_3_-19 and confirmed them to be 1R*, 2R* and 4S*, as shown for **2** [6,7,25,26,27]. The configuration at C-17 remains unresolved. Compound **2** is thus best described as (1R*,2R*,4S*,15*E*)-loba-8,10,13(14),15(16)-tetraen-17,18-diol-17-acetate.

**Table 2:** ^1^H and ^13^C NMR data (300 MHz and 75 MHz, CD_3_OD) for (1R*,2R*,4S*,15*E*)-loba-8,10,13(14),15(16)-tetraen-17,18-diol-17-acetate (**2**).

No.	^13^C δ (m)	^1^H δ (m, *J* Hz)	COSY	gHMBC	nOe
1	41.0 (s)				
2	54.1(d)	2.13 (1H, m)	H-3	C-1, C-4, C-7, C-10, C-11, C-12	H-4, H-12
3	35.2 (t)	1.66 (2H, m)	H-2, H-4	C-2, C-4	
4	41.5 (d)	2.31 (1H, tdd, 3.4, 4.2, 11.7)	H-3, H_a_-5, H_b_-5	C-3, C-13, C-14	H-2, H_a_-5, H_b_-5, H_a_-6, H-14, H-15, H-16, H-19, H-20, O-CO-CH_3_
5	28.8 (t)	1.64 (1H, m)	H-4, H_b_-5, H_a_-6	C-3	H-4
		1.52 (1H, m)	H-4, H_a_-5	C-1, C-4	H-2, H-4
6	41.2 (t)	1.60 (1H, m)	H_a_-5, H_b_-6	C-1, C-2, C-4, C-5, C-7	H-4, H-12
		1.45 (1H, m)	H_a_-6	C-2, C-4, C-5	
7	17.1 (q)	1.03 (3H, s)		C-1, C-2, C-6, C-8	H-12
8	151.6 (d)	5.87 (1H, dd, 10.8, 17.6)	H_a_-9	C-1, C-2, C-6, C-7	H-12
9	110.4 (t)	4.93 (1H, dd, 1.2. 17.6)	H-8, H_b_-9	C-1, C-2, C-8	H-12, H-17
		4.90 (1H, dd, 1.2, 10.8)	H-8, H_b_-9	C-1, C-2, C-8	H-17
10	148.9 (s)				
11	112.7 (t)	4.81 (1H, brt, 1.5)	H_b_-11, H_3_-12	C-1, C-2, C-10, C-12	H-12
		4.59 (1H, brs)	H_a_-11, H_3_-12	C-1, C-2, C-10, C-12	H-12
12	25.3 (q)	1.71 (3H, brs)	H_a_-11, H_b_-11	C-1, C-2, C-10, C-11	H-2, H_a_-6, H-7, H-8, H_a_-9, H_a_-11, H_b_-11, H-19
13	151.9 (s)				
14	114.3 (t)	5.06 (2H, s)		C-4, C-13, C-15, C-16	H-4
15	137.4(d)	6.26 (1H, d, 16.0)	H-16	C-4, C-13, C-14, C-16, C-17	H-4, H-17
16	124.4 (d)	5.78 (1H, dd, 7.6, 16.0)	H-15, H-17	C-13,C-14, C-17, C-18	H-4, H-17
17	82.2 (d)	5.14 (1H, brd, 7.6)	H-16	C-15, C-16, C-18, C-19, C-20, O-CO-CH_3_	H_a_-9, H_b_-9, H-15, H-16, H-19, H-20, O-CO-CH_3_
18	72.7 (s)				
19	25.6 (q)	1.17 (3H, s)		C-17, C-18, C-20	H-4, H-12, H-17, O-CO-CH_3_
20	26.2 (q)	1.18 (3H, s)		C-17, C-18, C-19	H-4, H-17, O-CO-CH_3_
O-CO-CH_3_	172.1 (s)				
O-CO-CH_3_	21.1 (q)	2.09 (3H, s)		OAc	H-4, H-7, H-17, H-19, H-20

Complete 1D and 2D NMR data for the known cembranes: sarcophytol-B and (1*E*,3*E*,7*E*)-11,12-epoxycembratrien-15-ol, and the six known lobanes: loba-8,10,13(15)-triene-16,17,18-triol, 14,18-epoxyloba-8,10,13(15)-trien-17-ol, lobatrientriol, lobatrienolide, 14,17-epoxyloba-8,10,13(15)-trien-18-ol-18-acetate and (17*R*)-loba-8,10,13(15)-trien-17,18-diol, are provided for the first time (Supplementary Information). Raju *et al.* reported that loba-8,10,13(15)-triene-16,17,18-triol was the product of long-term, cold storage of the natural product 17,18-epoxyloba-8,10,13(15)-trien-16-ol in CDC1_3_ [25]. Closer inspection of the FTMS and ^13^C NMR of the fresh extract in CD_3_OD showed the presence of only the triol in our study.

The cytotoxic activities of compounds **1** and **2**, and of the known compounds loba-8,10,13(15)-triene-16,17,18-triol, 14,17-epoxyloba-8,10,13(15)-trien-18-ol-18-acetate, lobatrienolide, (1*E*,3*E*,7*E*)-11,12-epoxycembratrien-15-ol and sarcophytol-B towards a panel of human tumour cell lines are given in Table 3. With the exception of **1** (GI_50_s all over 70 μM), all compounds showed good activity with GI_50_s in the range 6.8–18.5 µM. From these data there appears to be no obvious SAR. The four lobanes (including **2**) and the two cembranes all had approximately the same overall activities against the human tumour cell lines SF-268, MCF-7 and H460, with no selectivity.

**Table 3:** Cytotoxicity data [GI_50_ (µM)] for compounds **1**, **2** and the known compounds loba-8,10,13(15)-triene-16,17,18-triol, 14,17-epoxyloba-8,10,13(15)-trien-18-ol-18-acetate, lobatrienolide, (1*E*,3*E*,7*E*)-11,12-epoxycembratrien-15-ol and sarcophytol-B against the human tumour cell lines SF-268, MCF-7 and H460.

Compound	SF-268 ^a^	MCF-7 ^b^	H460 ^c^
**1**	175	70	125
**2**	15	8.8	11.5
Loba-8,10,13(15)-triene-16,17,18-triol	18.5	17	13
Lobatrientriol	NT	NT	NT
14,17-Epoxyloba-8,10,13(15)-trien-18-ol-18-acetate	14	16	18.5
Lobatrienolide	7.4	17	18
14,18-Epoxyloba-8,10,13(15)-trien-17-ol	NT	NT	NT
(17*R*)-Loba-8,10,13(15)-trien-17,18-diol	NT	NT	NT
(1*E*,3*E*,7*E*)-11,12-Epoxycembratrien-15-ol	6.8	12	18.5
Sarcophytol-B	16	12.5	15

^a^ SF-268 Central nervous system-glioblastoma cells; ^b^ MCF-7 Breast-pleural effusion adenocarcinoma cells; ^c^ H460 Lung-large cell carcinoma cells; NT = Not tested.

## 3. Experimental Section

### 3.1. General Experimental Procedures

General experimental procedures are as described previously [29].

### 3.2. Animal Material

The soft coral *Sinularia* sp., (Order Alcyonacea, Family Alcyoniidae) was collected from the eastern edge of the lagoon at Bowden Reef (19°2.1′S, 147°56.0′E) in the Central Great Barrier Reef, Queensland, Australia, at a depth of 9 m, in June 2005. Collection of this material was conducted under the GBRMPA Permit no. G05/11866.1 and kept frozen (−20 °C) until work-up. A voucher specimen (AIMS 27026) has been lodged with the AIMS Bioresources Library.

### 3.3. Bioassay

Cellular bioassays were undertaken as described previously [29].

### 3.4. Extraction and Isolation

Freeze dried animal material (29.6 g) was extracted with MeOH (3 × 400 mL) and a butanol:CH_2_Cl_2_:H_2_O (150:50:100 mL) partition performed. The aqueous phase was further partitioned with BuOH:CH_2_Cl_2_ (150:50 mL) and the organic phase added to the first organic fraction. The organic fraction (16.8 g) was then subjected to reversed phase C18 flash vacuum chromatography (RP-C18, 25%, 50%, 75%, 100% MeOH in H_2_O and 1:1 MeOH:CH_2_Cl_2_). Activity was observed for the first four fractions. A portion of the 25% MeOH fraction (3.44 g of 10.27 g) was pre-absorbed onto C18, packed into a cartridge, and further separated by preparative C18 HPLC (52 mL/min, isocratic elution at 15% CH_3_CN:H_2_O for 3 min followed by gradient elution from 15% CH_3_CN:H_2_O to 100% CH_3_CN:H_2_O over 50 min and an isocratic elution at 100% CH_3_CN for 30 min through a 250 × 41.1 mm Varian Dynamax Microsorb 60-8 C18 column), fractions were collected every 30 s (*n* = 176) to yield (in order of elution) (4R*,5R*,9S*,10R*,11*Z*)-4-methoxy-9-((dimethylamino)-methyl)-12,15-epoxy-11(13)-en-decahydronaphthalen-16-ol (**1**, fr 19 and 20 combined, 18.3 mg, 0.06% dry wt of extract), lobatrientriol [7] (fr 71, 24.6 mg, 0.08% dry wt of extract), loba-8,10,13(15)-triene-16,17,18-triol (fr 81, 21.1 mg, 0.07% dry wt of extract), 14,17-epoxyloba-8,10,13(15)-trien-18-ol-18-acetate [26] (fr, 83 and 84 combined, 72.8 mg, 0.07% dry wt of extract), lobatrienolide [7] (fr 87, 24.9 mg, 0.08% dry wt of extract), (1*E*,3*E*,7*E*)-11,12-epoxycembratrien-15-ol [8] (fr 89, 30.9 mg, 0.10% dry wt of extract) and 14,18-epoxyloba-8,10,13(15)-trien-17-ol [26] (fr 109, 123.7 mg, 0.42% dry wt of extract). Fractions 100 to 102 were combined (108.4 mg) and further purified by C18 analytical HPLC (1 mL/min, gradient elution from 5% CH_3_CN:H_2_O to 100% CH_3_CN over 18 min, followed by 6 min with 100% CH_3_CN through 250 × 4.6 mm, 5μ Phenomenex Luna (2) C18 column and fractions collected every 30 s) to yield the known compounds (17*R*)-loba-8,10,13(15)-triene-17,18-diol [27] (fr 34, 6.2 mg, 0.02% dry wt of extract) and sarcophytol-B [24] (fr 36, 18.3 mg, 0.06% dry wt of extract) and the new compound (1R*,2R*,4S*,15*E*)-loba-8,10,13(14),15(16)-tetraen-17,18-diol-17-acetate (**2**, fr 33, 2.3 mg, 0.008% dry wt of extract). The known compounds had identical physical and spectroscopic properties to those previously published [7,8,24,26,27].

#### 3.4.1. (4R*,5R*,9S*,10R*,11*Z*)-4-Methoxy-9-((dimethylamino)-methyl)-12,15-epoxy-11(13)-en-decahydronaphthalen-16-ol (**1**)

Pale yellow oil. [α]^24^_D_ +72° (CH_3_OH; *c* 0.67); UV (PDA) λ_max_ nm: 195, 208; IR ν_max_ cm^−1^: 3388, 2969, 2935, 1645, 1468, 1384, 1161, 1080; ^1^H (300 MHz, CD_3_OD) and ^13^C (75 MHz, CD_3_OD) NMR data Table 1; ESI-FTMS *m/z* [M + H]^+^ 394.3316 (calcd for C_24_H_44_O_3_N 394.3303), [M + Na]^+^ 416.3128 (calcd for C_24_H_43_O_3_NNa 416.3135).

#### 3.4.2. (1R*,2R*,4S*,15*E*)-Loba-8,10,13(14),15(16)-tetraen-17,18-diol-17-acetate (**2**)

Colourless oil. [α]^24^_D_ −9.5° (CH_3_OH; *c* 0.23); UV (PDA) λ_max_ nm: 203, 227; IR ν_max_ cm^−1^: 3408, 2963, 2926, 1734, 1635, 1455, 1372, 1234, 1024, 904; ^1^H (300 MHz, CD_3_OD) and ^13^C (75 MHz, CD_3_OD) NMR data Table 2; ESI-FTMS *m/z* [M + Na]^+^ 369.2396 (calcd for C_22_H_34_O_3_Na 369.2400).

#### 3.4.3. Loba-8,10,13(15)-triene-16,17,18-triol

Colourless oil. ^1^H-NMR and ^13^C-NMR spectral data were consistent with published values [25]; ESI-FTMS *m/z* [M + Na]^+^ 345.2398 (calcd for C_20_H_34_O_3_Na 345.2400).

#### 3.4.4. Lobatrientriol

Colourless oil. ^1^H-NMR and ^13^C-NMR spectral data were consistent with published values [7].

#### 3.4.5. 14,17-Epoxyloba-8,10,13(15)-trien-18-ol-18-acetate

Colourless oil. ^1^H-NMR and ^13^C-NMR spectral data were consistent with published values [26].

#### 3.4.6. Lobatrienolide

Colourless oil. ^1^H-NMR and ^13^C-NMR spectral data were consistent with published values [7].

#### 3.4.7. (1*E*,3*E*,7*E*)-11,12-Epoxycembratrien-15-ol

Colourless oil. ^1^H-NMR and ^13^C-NMR spectral data were consistent with published values [8].

#### 3.4.8. 14,18-Epoxyloba-8,10,13(15)-trien-17-ol

Colourless oil. ^1^H-NMR and ^13^C-NMR spectral data were consistent with published values [26].

#### 3.4.9. (17*R*)-Loba-8,10,13(15)-trien-17,18-diol

Colourless oil. ^1^H-NMR and ^13^C-NMR spectral data were consistent with published values [27].

#### 3.4.10. Sarcophytol-B

Colourless oil. ^1^H-NMR and ^13^C-NMR spectral data were consistent with published values [24].

## 4. Conclusion

Two new compounds, the somewhat unprecedented nitrogen containing (4R*,5R*,9S*,10R*,11*Z*)-4-methoxy-9-((dimethylamino)-methyl)-12,15-epoxy-11(13)-en-decahydronaphthalen-16-ol (**1**) and the lobane (1R*,2R*,4S*,15*E*)-loba-8,10,13(14),15(16)-tetraen-17,18-diol-17-acetate (**2**), together with the eight known compounds sarcophytol-B [24], (1*E*,3*E*,7*E*)-11,12-epoxycembratrien-15-ol [8], loba-8,10,13(15)-triene-16,17,18-triol [25], 14,18-epoxyloba-8,10,13(15)-trien-17-ol [26], lobatrientriol [7], lobatrienolide [7], 14,17-epoxyloba-8,10,13(15)-trien-18-ol-18-acetate [26] and (17*R*)-loba-8,10,13(15)-trien-17,18-diol [27], were isolated from the Australian soft coral *Sinularia* sp. Although there are many publications detailing the isolation of lobanes [6,7] and cembranes [8,9,11] from soft corals of the genus *Sinularia*, this report shows that new investigations are still yielding further new and somewhat unprecedented derivatives, and that continued investigations of this genus are warranted. The biological and pharmacological properties associated with soft coral chemistry, in particular terpenoids, have been shown to be highly promising [30], leading to the need for more extensive structure-activity relationship studies and further evaluation of their mechanism of action.

## Supplementary Files

Supplementary File 1: PDF-Document (PDF, 5848 KB)
