# Supplementary material for: A Great Barrier Reef Sinularia sp. Yields Two New Cytotoxic Diterpenes
Source: Mar Drugs. 2012 Jul 31;10(8):1619–30. doi: 10.3390/md10081619 (PMC3447330; doi:10.3390/md10081619)
Supplement: Supplementary File 1: — PDF-Document (PDF, 5848 KB) [file marinedrugs-10-01619-s001.pdf]

Supplementary Information

Table of Contents.

Page No

Compound **1**: (4*R**,5*R**,9*S**,10*R**,11*Z*)-4-methoxy-12,15-epoxy-11(13)-en-9-((dimethylamino)-methyl)-decadehydronaphthalen-16-ol.
Compound **2**: (1*R**,2*R**,4*S**,15*E*)-loba-8,10,13(14),15(16)-tetraen-17,18-diol-17-acetate

Figure S1. ^{1}H NMR spectrum (300 MHz) of (**1**) in CD_3OD........ 5
Figure S2. ^{13}C NMR spectrum (75 MHz) of (**1**) in CD_3OD........ 6
Figure S3. DEPT spectrum (75 MHz) of (**1**) in CD_3OD........ 7
Figure S4. COSY spectrum (300 MHz) of (**1**) in CD_3OD........ 8
Figure S5. HSQC spectrum (300 MHz) of (**1**) in CD_3OD. 9
Figure S6. HMBC spectrum (300 MHz) of (**1**) in CD_3OD........ 10
Figure S7. Selective NOESY spectrum (300 MHz; 4.16 ppm) of (**1**) in CD_3OD 11
Figure S8. Selective NOESY spectrum (300 MHz; 3.25 ppm) of (**1**) in CD_3OD 12
Figure S9. Selective NOESY spectrum (300 MHz; 3.16 ppm) of (**1**) in CD_3OD 13
Figure S10. Selective NOESY spectrum (300 MHz; 1.08 ppm) of (**1**) in CD_3OD 14
Figure S11. Selective NOESY spectrum (300 MHz; 0.89 ppm) of (**1**) in CD_3OD 15
Figure S12. ^{1}H NMR spectrum (300 MHz) of (**2**) in CD_3OD........ 16
Figure S13. ^{13}C NMR spectrum (75 MHz) of (**2**) in CD_3OD........ 17
Figure S14. DEPT spectrum (75 MHz) of (**2**) in CD_3OD........ 18
Figure S15. COSY spectrum (300 MHz) of (**2**) in CD_3OD........ 19
Figure S16. HSQC spectrum (300 MHz) of (**2**) in CD_3OD. 20
Figure S17. HMBC spectrum (300 MHz) of (**2**) in CD_3OD........ 21

Figure S18. Selective NOESY spectrum (300 MHz; 5.14 ppm) of (**2**) in CD_3OD 22
Figure S19. Selective NOESY spectrum (300 MHz; 2.31 ppm) of (**2**) in CD_3OD 23
Figure S20. Selective NOESY spectrum (300 MHz; 2.09 ppm) of (**2**) in CD_3OD 24
Figure S21. Selective NOESY spectrum (300 MHz; 1.71 ppm) of (**2**) in CD_3OD 25
Figure S22. Selective NOESY spectrum (300 MHz; 1.03 ppm) of (**2**) in CD_3OD 26
Scheme 1: Structures of known compounds isolated from *Sinularia* sp. 27
Figure S23. ^{1}H NMR spectrum (300 MHz) of loba-8,10,13(15)-triene-16,17,18-triol in CD_3OD.. 28
Figure S24. ^{13}C NMR spectrum (75 MHz) of loba-8,10,13(15)-triene-16,17,18-triol in CD_3OD.. 29
Figure S25. DEPT spectrum (75 MHz) of loba-8,10,13(15)-triene-16,17,18-triol in CD_3OD.. 30
Figure S26. COSY spectrum (300 MHz) of loba-8,10,13(15)-triene-16,17,18-triol in CD_3OD.. 31
Figure S27. HSQC spectrum (300 MHz) of loba-8,10,13(15)-triene-16,17,18-triol in CD_3OD. 32
Figure S28. HMBC spectrum (300 MHz) of loba-8,10,13(15)-triene-16,17,18-triol in CD_3OD.. 33
Figure S29. Selective NOESY spectrum (300 MHz; 4.65 ppm) of loba-8,10,13(15)-triene-16,17,18-triol in CD_3OD 34
Figure S30. Selective NOESY spectrum (300 MHz; 3.15 ppm) of loba-8,10,13(15)-triene-16,17,18-triol in CD_3OD 35
Figure S31. Selective NOESY spectrum (300 MHz; 1.74 ppm) of loba-8,10,13(15)-triene-16,17,18-triol in CD_3OD 36
Figure S32. Selective NOESY spectrum (300 MHz; 1.71 ppm) of loba-8,10,13(15)-triene-16,17,18-triol in CD_3OD 37
Figure S33. Selective NOESY spectrum (300 MHz; 1.25 ppm) of loba-8,10,13(15)-triene-16,17,18-triol in CD_3OD 38
Figure S34. Selective NOESY spectrum (300 MHz; 1.23 ppm) of loba-8,10,13(15)-triene-16,17,18-triol in CD_3OD 39
Figure S35. Selective NOESY spectrum (300 MHz; 1.02 ppm) of loba-8,10,13(15)-triene-16,17,18-triol in CD_3OD 40
Table S1. ^{1}H and ^{13}C NMR data (300 MHz and 75 MHz, CD_3OD) for loba-8,10,13(15)-triene-16,17,18-triol.. 41
Figure S36. ^{1}H NMR spectrum (300 MHz) of lobatrienolide in CD_3OD.. 42
Figure S37. ^{13}C NMR spectrum (75 MHz) of lobatrienolide in CD_3OD.. 43
Figure S38. DEPT spectrum (75 MHz) of lobatrienolide in CD_3OD.. 44
Figure S39. COSY spectrum (75 MHz) of lobatrienolide in CD_3OD.. 45
Figure S40. HSQC spectrum (300 MHz) of lobatrienolide in CD_3OD.. 46
Figure S41. HMBC spectrum (300 MHz) of lobatrienolide in CD_3OD.. 47
Table S2. ^{1}H and ^{13}C NMR data (300 MHz and 75 MHz, CD_3OD) for lobatrienolide.. 48
Figure S42. ^{1}H NMR spectrum (300 MHz) of lobatrientriol in CD_3OD.. 49

Figure S43. ^{13}C NMR spectrum (75 MHz) of lobatrientriol in CD_3OD... 50
Figure S44. COSY spectrum (300 MHz) of lobatrientriol in CD_3OD... 51
Figure S45. HSQC spectrum (300 MHz) of lobatrientriol in CD_3OD... 52
Figure S46. HMBC spectrum (300 MHz) of lobatrientriol in CD_3OD... 53
Table S3. ^{1}H and ^{13}C NMR data (300 MHz and 75 MHz, $CDCl_3$) for lobatrientriol.. 54
Figure S47. ^{1}H NMR spectrum (300 MHz) of 14,18-epoxyloba-8,10,13(15)-trien-17-ol in CD_3OD.. 55
Figure S48. ^{13}C NMR spectrum (75 MHz) of 14,18-epoxyloba-8,10,13(15)-trien-17-ol in CD_3OD.. 56
Figure S49. DEPT spectrum (75 MHz) of 14,18-epoxyloba-8,10,13(15)-trien-17-ol in CD_3OD.. 57
Figure S50. COSY spectrum (300 MHz) of 14,18-epoxyloba-8,10,13(15)-trien-17-ol in CD_3OD.. 58
Figure S51. HSQC spectrum (300 MHz) of 14,18-epoxyloba-8,10,13(15)-trien-17-ol in CD_3OD.. 59
Figure S52. HMBC spectrum (300 MHz) of 14,18-epoxyloba-8,10,13(15)-trien-17-ol in CD_3OD.. 60
Table S4. ^{1}H and ^{13}C NMR data (300 MHz and 75 MHz, CD_3OD) for 14,18-epoxyloba-8,10,13(15)-trien-17-ol................................ 61
Figure S53. ^{1}H NMR spectrum (300 MHz) of 14,17-epoxyloba-8,10,13(15)-trien-18-ol-18-acetate in CD_3OD..................................... 62
Figure S54. ^{13}C NMR spectrum (75 MHz) of 14,17-epoxyloba-8,10,13(15)-trien-18-ol-18-acetate in CD_3OD.................................... 63
Figure S55. DEPT spectrum (75 MHz) of 14,17-epoxyloba-8,10,13(15)-trien-18-ol-18-acetate in CD_3OD... 64
Figure S56. COSY spectrum (300 MHz) of 14,17-epoxyloba-8,10,13(15)-trien-18-ol-18-acetate in CD_3OD... 65
Figure S57. HSQC spectrum (300 MHz) of 14,17-epoxyloba-8,10,13(15)-trien-18-ol-18-acetate in CD_3OD... 66
Figure S58. HMBC spectrum (300 MHz) of 14,17-epoxyloba-8,10,13(15)-trien-18-ol-18-acetate in CD_3OD....................................... 67
Table S5. ^{1}H and ^{13}C NMR data (300 MHz and 75 MHz, CD_3OD) for 14,17-epoxyloba-8,10,13(15)-trien-18-ol-18-acetate............. 68
Figure S59. ^{1}H NMR spectrum (300 MHz) of (17*R*)-loba-8,10,13(15)-trien-17,18-diol in CD_3OD... 69
Figure S60. ^{13}C NMR spectrum (75 MHz) of (17*R*)-loba-8,10,13(15)-trien-17,18-diol in CD_3OD... 70
Figure S61. COSY spectrum (300 MHz) of (17*R*)-loba-8,10,13(15)-trien-17,18-diol in CD_3OD... 71
Figure S62. HSQC spectrum (300 MHz) of (17*R*)-loba-8,10,13(15)-trien-17,18-diol in CD_3OD... 72
Figure S63. HMBC spectrum (300 MHz) of (17*R*)-loba-8,10,13(15)-trien-17,18-diol in CD_3OD... 73
Table S6. ^{1}H and ^{13}C NMR data (300 MHz and 75 MHz, CD_3OD) for (17*R*)-loba-8,10,13(15)-trien-17,18-diol ……………............... 74
Figure S64. ^{1}H NMR spectrum (300 MHz) of sarcophytol-B in CD_3OD.. 75
Figure S65. ^{13}C NMR spectrum (75 MHz) of sarcophytol-B in CD_3OD.. 76
Figure S66. DEPT spectrum (75 MHz) of sarcophytol-B in CD_3OD... 77

Figure S67. COSY spectrum (300 MHz) of sarcophytol-B in CD_3OD.. 78
Figure S68. HSQC spectrum (300 MHz) of sarcophytol-B in CD_3OD.. 79
Figure S69. HMBC spectrum (300 MHz) of sarcophytol-B in CD_3OD... 80
Table S7.^{1}H and ^{13}C NMR data (300 MHz and 75 MHz, CD_3OD) for sarcophytol-B………………..……………………….................. 81
Figure S70. ^{1}H NMR spectrum (300 MHz) of (1*E*,3*E*,7*E*)-11,12-epoxycembratrien-15-ol in CD_3OD.. 82
Figure S71. ^{13}C NMR spectrum (75 MHz) of 11(1*E*,3*E*,7*E*)-11,12-epoxycembratrien-15-ol in CD_3OD... 83
Figure S72. DEPT spectrum (75 MHz) of (1*E*,3*E*,7*E*)-11,12-epoxycembratrien-15-ol in CD_3OD... 84
Figure S73. COSY spectrum (300 MHz) of (1*E*,3*E*,7*E*)-11,12-epoxycembratrien-15-ol in CD_3OD... 85
Figure S74. HSQC spectrum (300 MHz) of (1*E*,3*E*,7*E*)-11,12-epoxycembratrien-15-ol in CD_3OD... 86
Figure S75. HMBC spectrum (300 MHz) of (1*E*,3*E*,7*E*)-11,12-epoxycembratrien-15-ol in CD_3OD... 87
Table S8. ^{1}H and ^{13}C NMR data (300 MHz and 75 MHz, CD_3OD) for (1*E*,3*E*,7*E*)-11,12-epoxycembratrien-15-ol.…………….......... 88

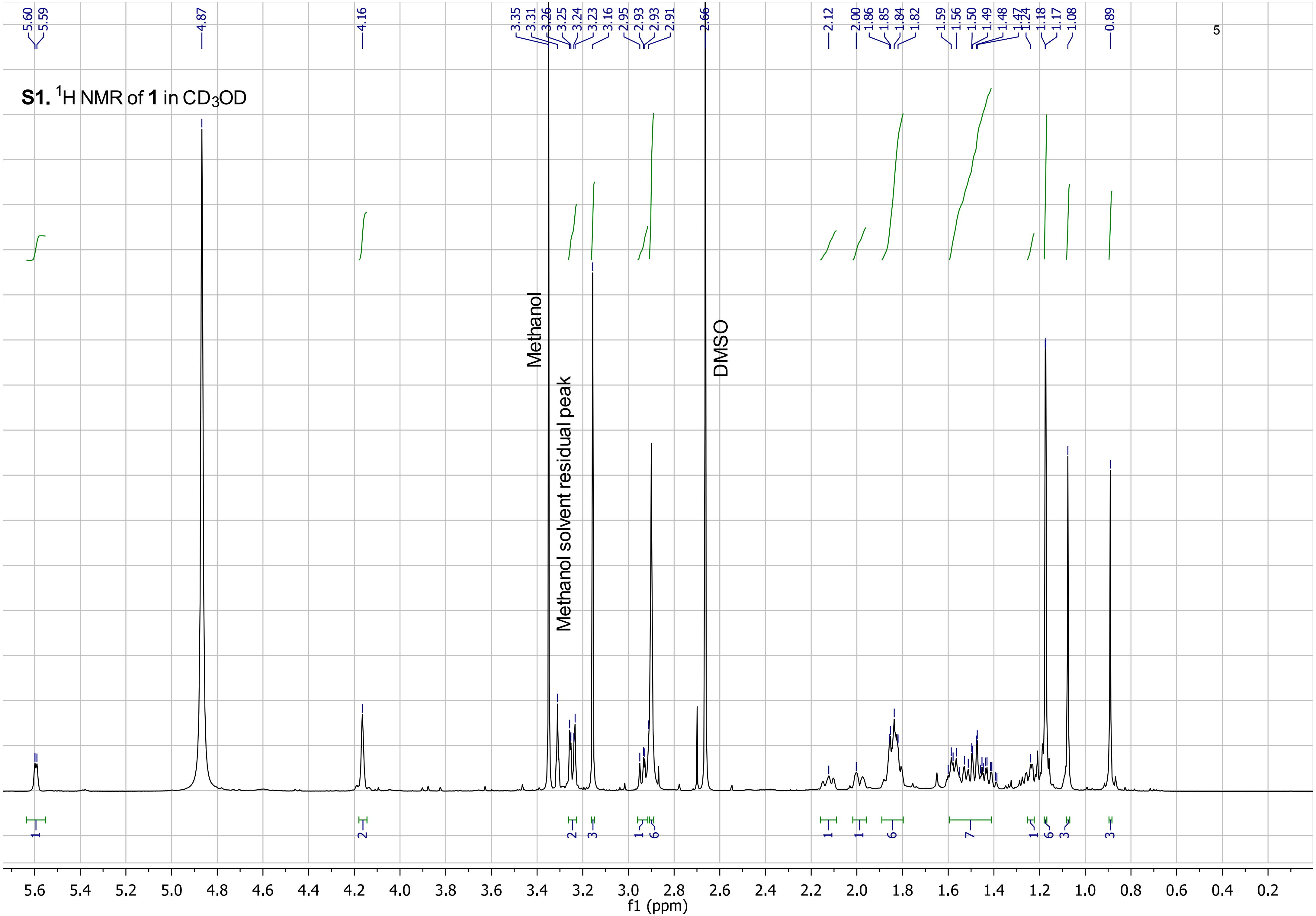

S1. ^{1}H NMR of **1** in CD_3OD

S2. ^{13}C NMR of **1** in CD_3OD

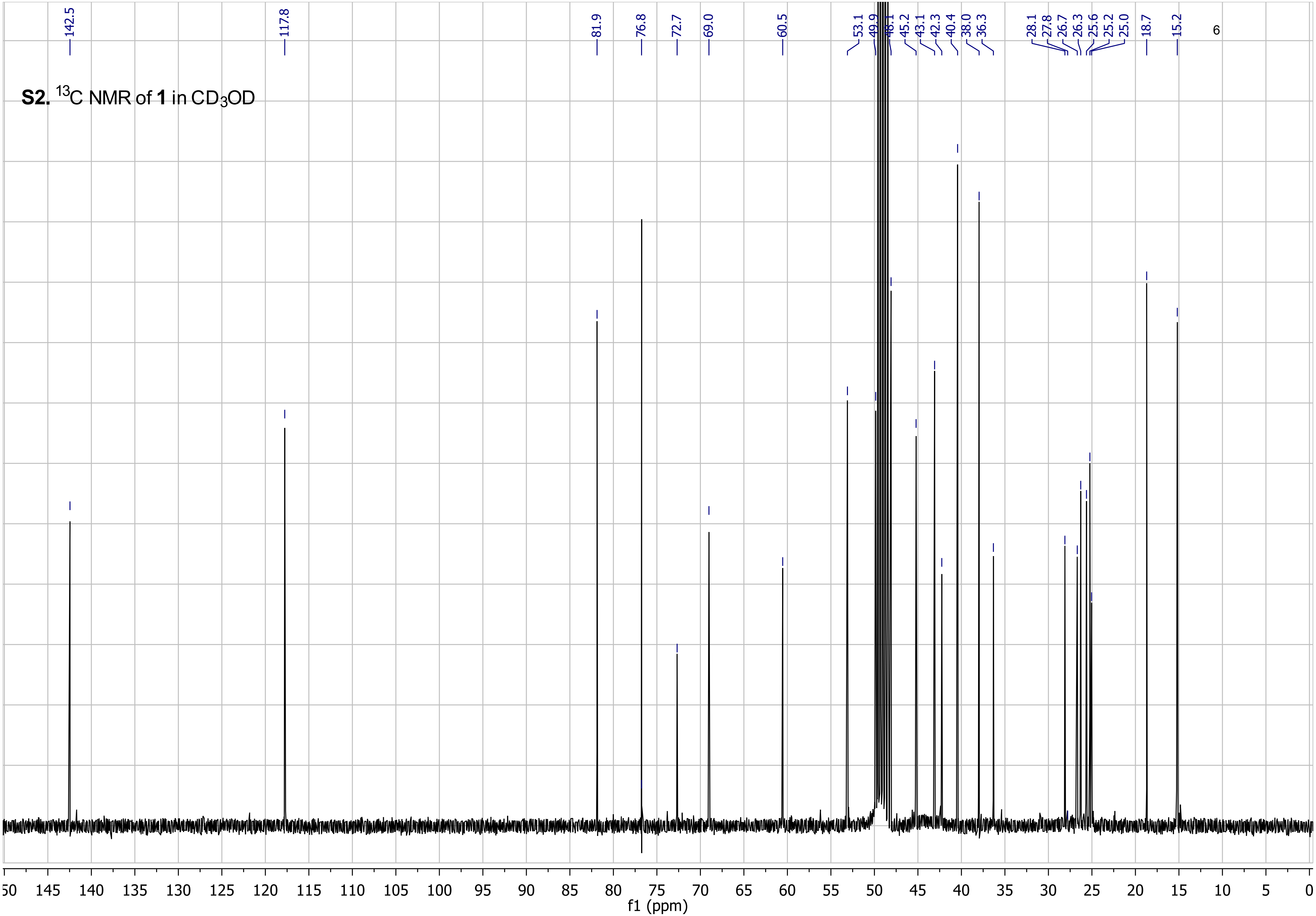

S3. DEPT NMR of **1** in CD_3OD

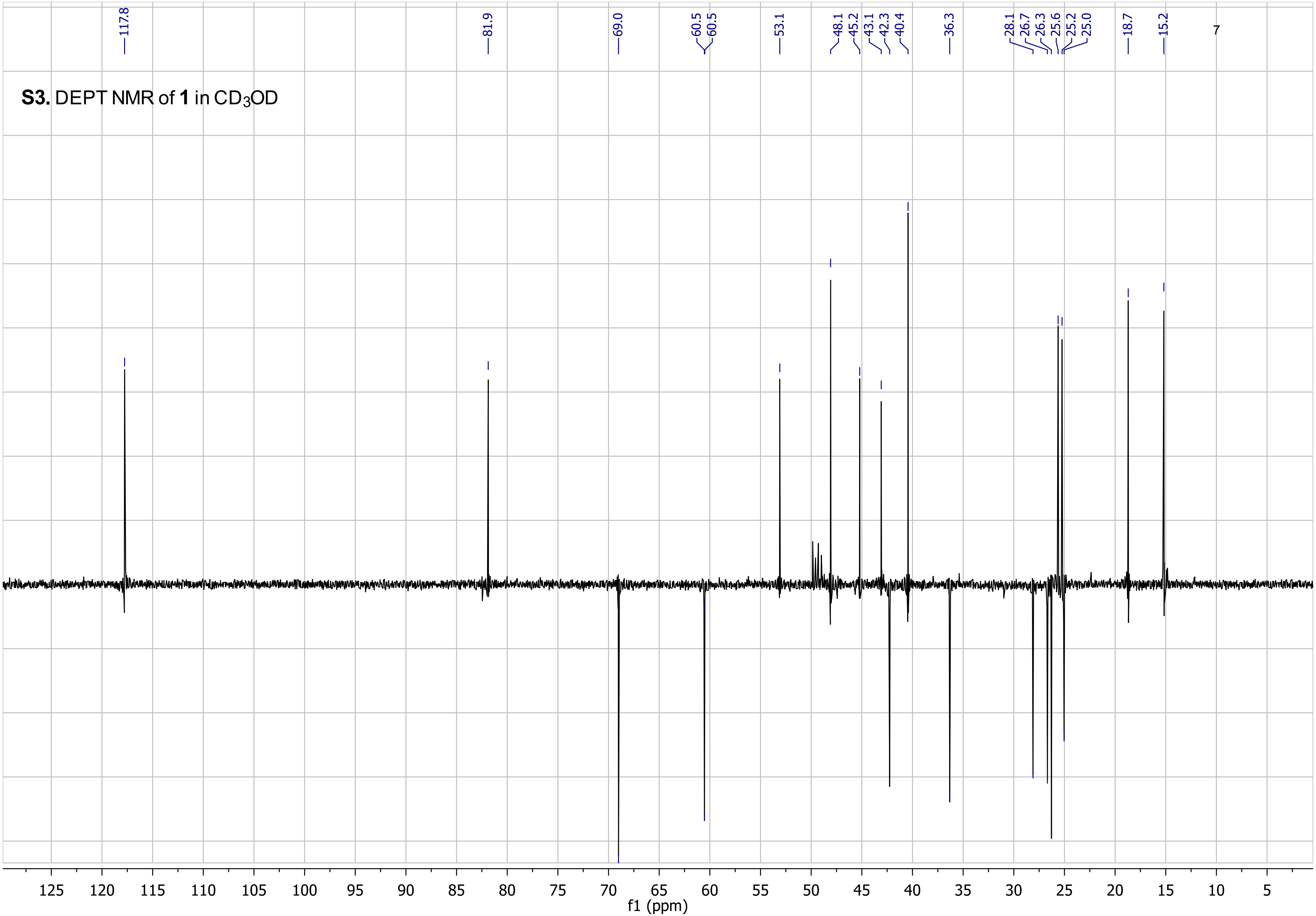

S4. COSY NMR of **1** in CD_3OD

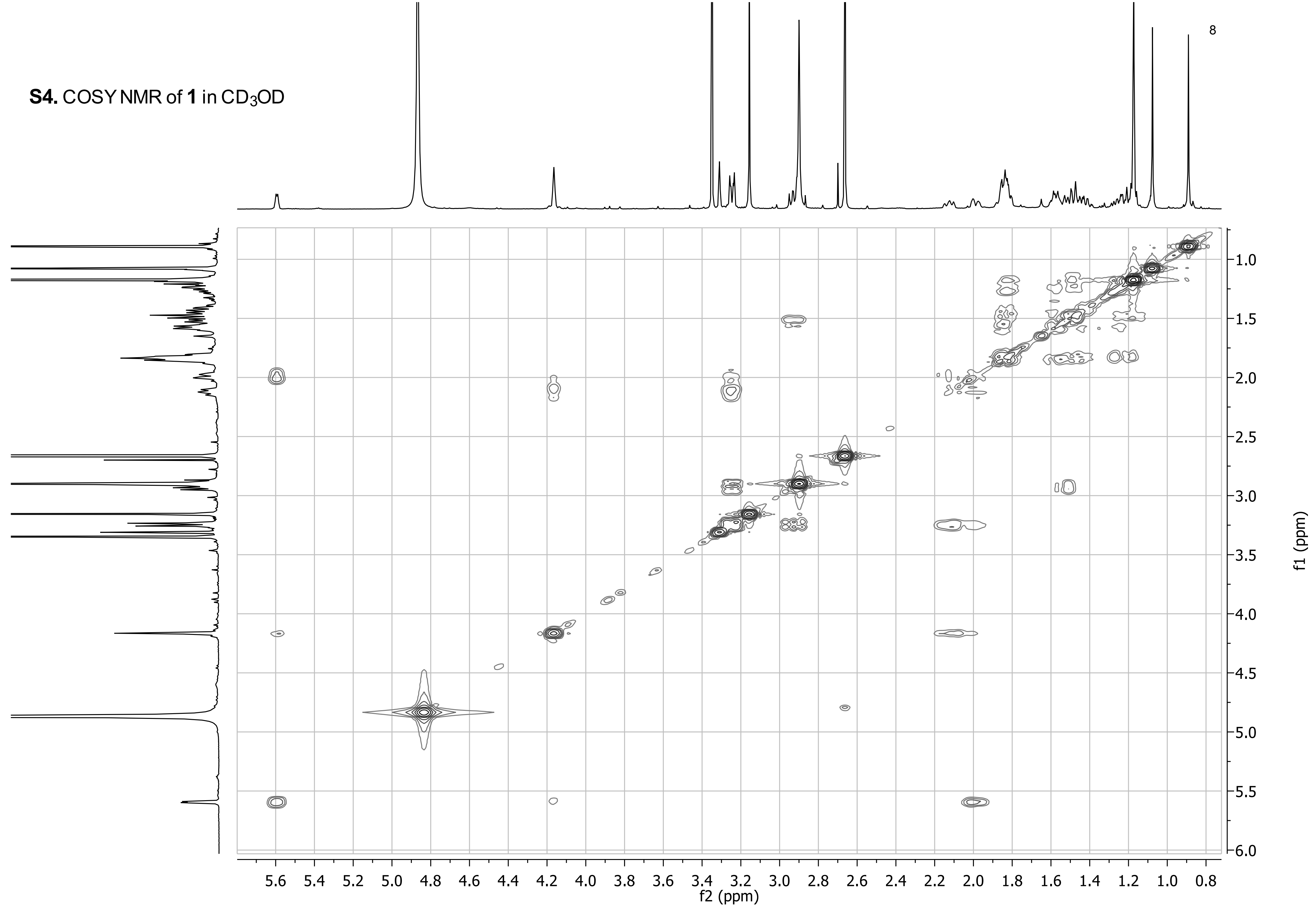

S5. HSQC NMR of **1** in CD_3OD

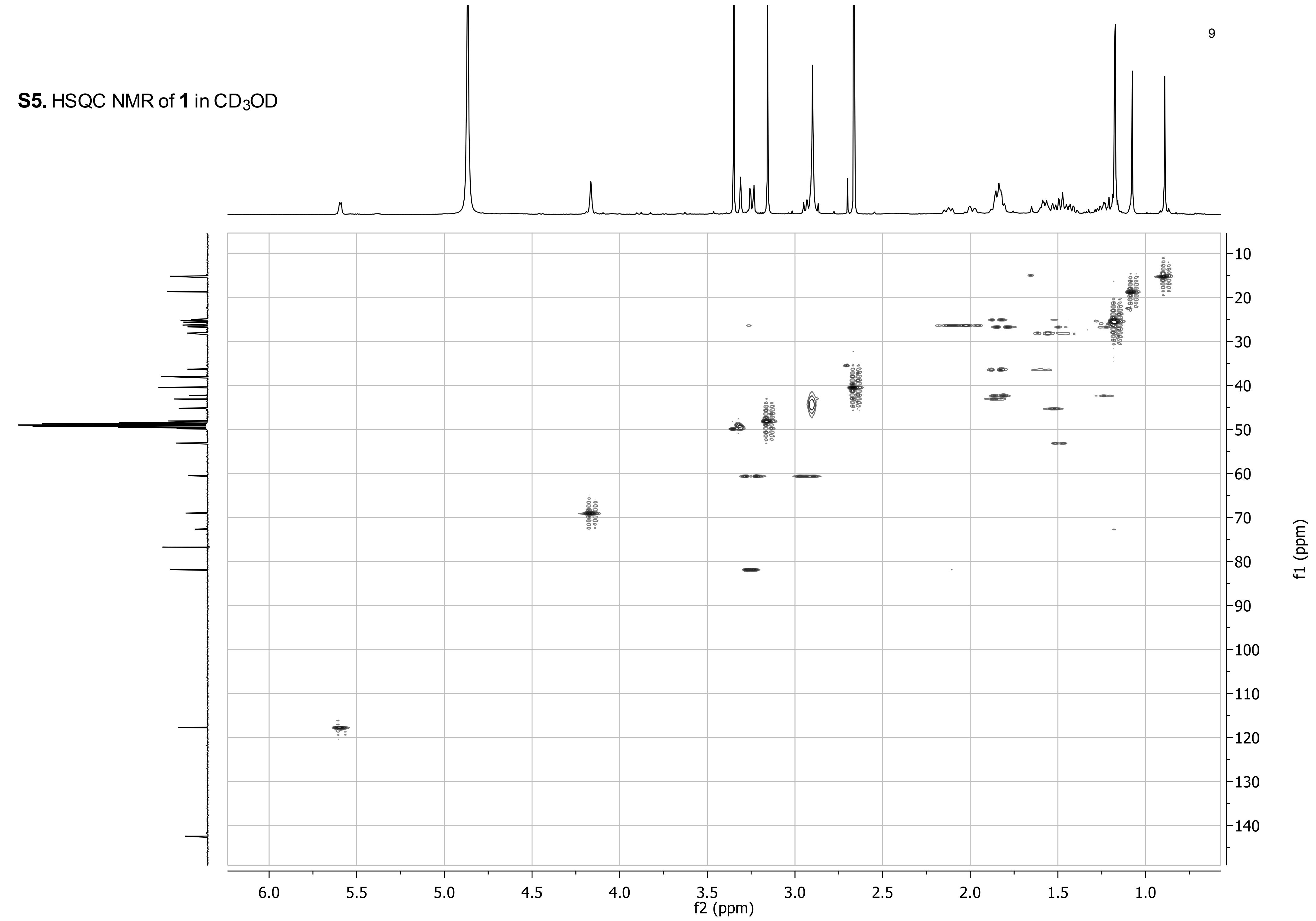

S6. HMBC NMR of **1** in CD_3OD

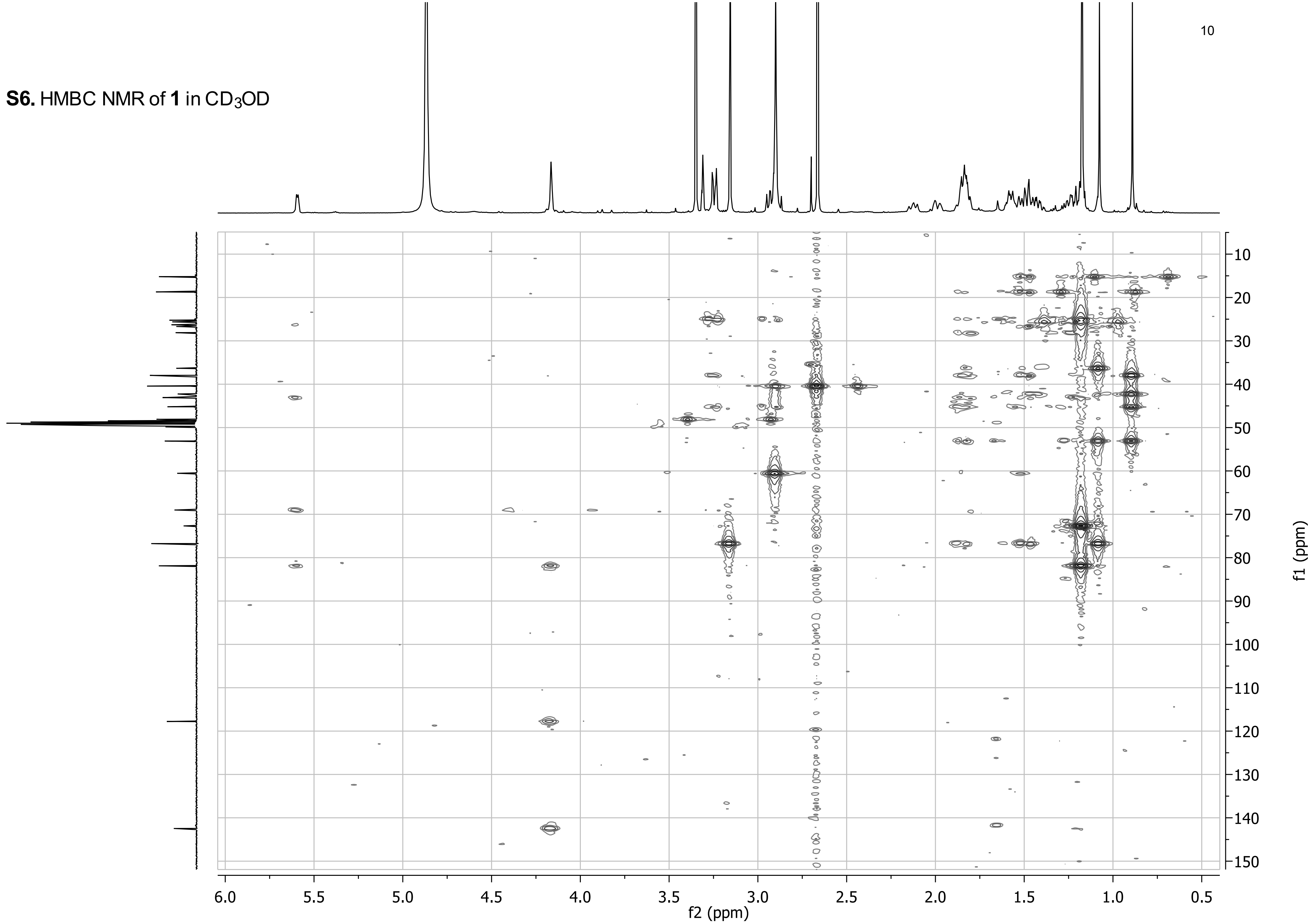

S7. Selective NOESY NMR of H-12 (4.16 ppm) in **1** in CD_3OD

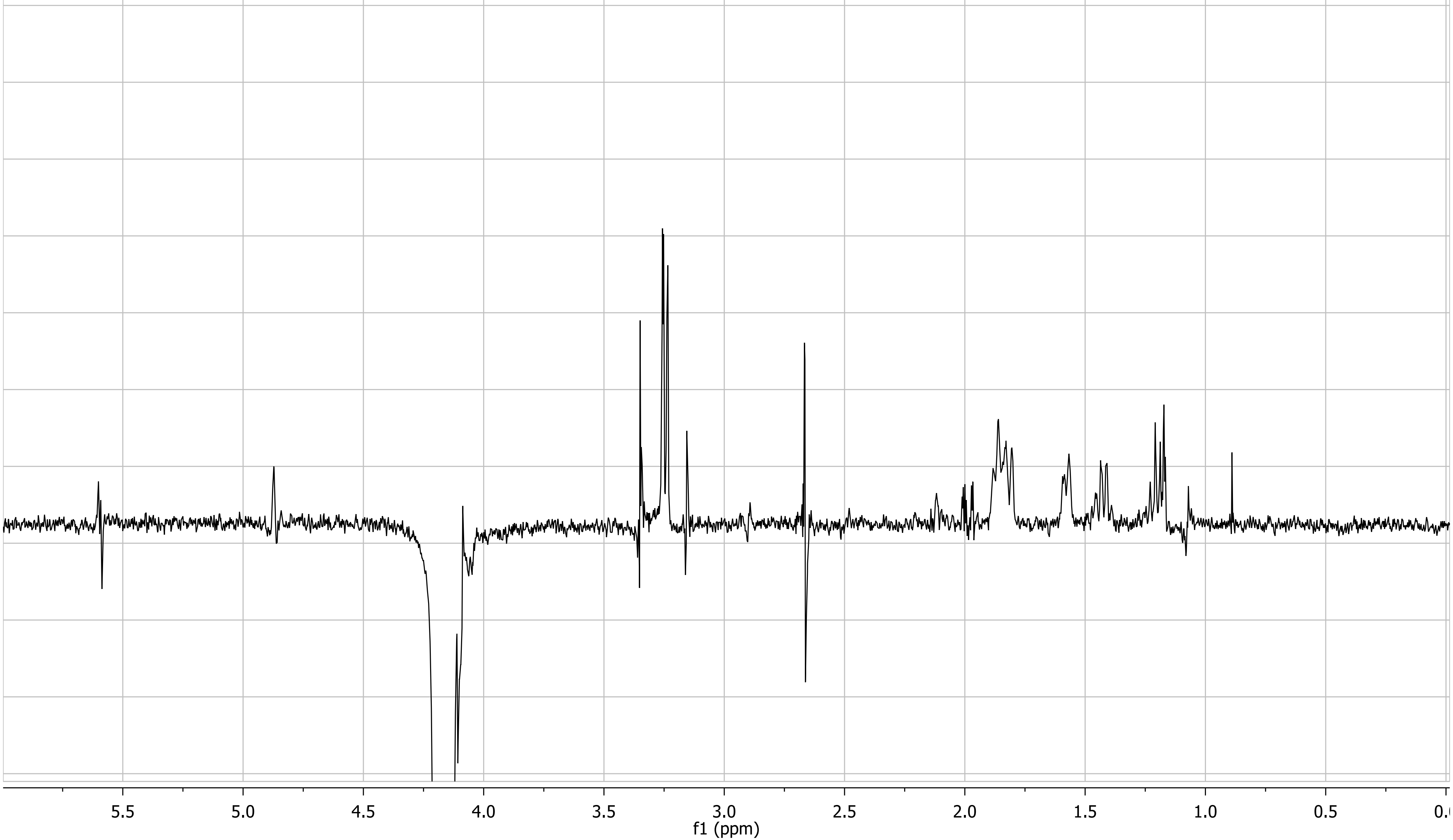

S8. Selective NOESY NMR of H-15/H-22 (3.25 ppm) in **1** in CD_3OD

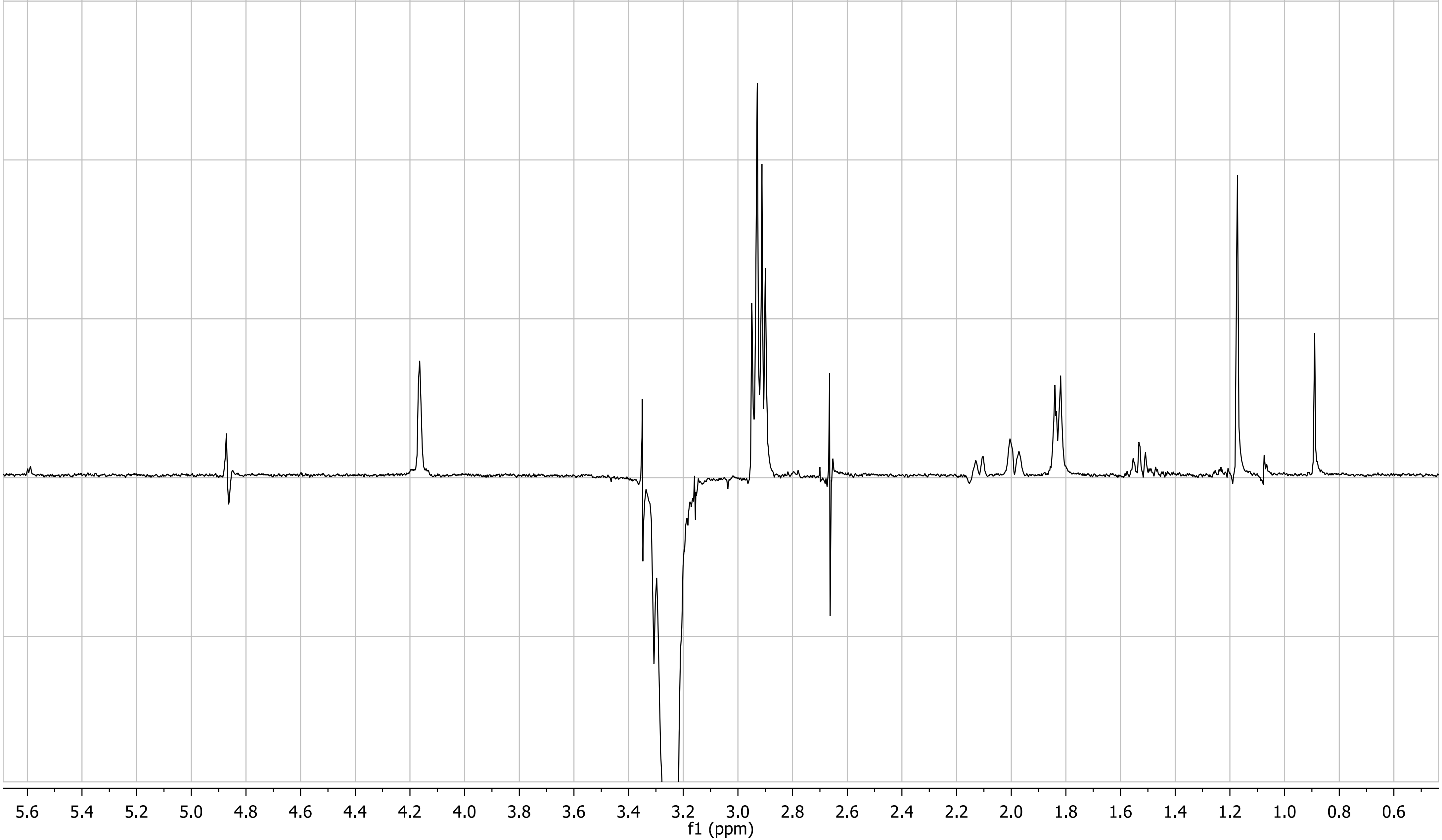

S9. Selective NOESY NMR of H-21 (3.16 ppm) in **1** in CD_3OD

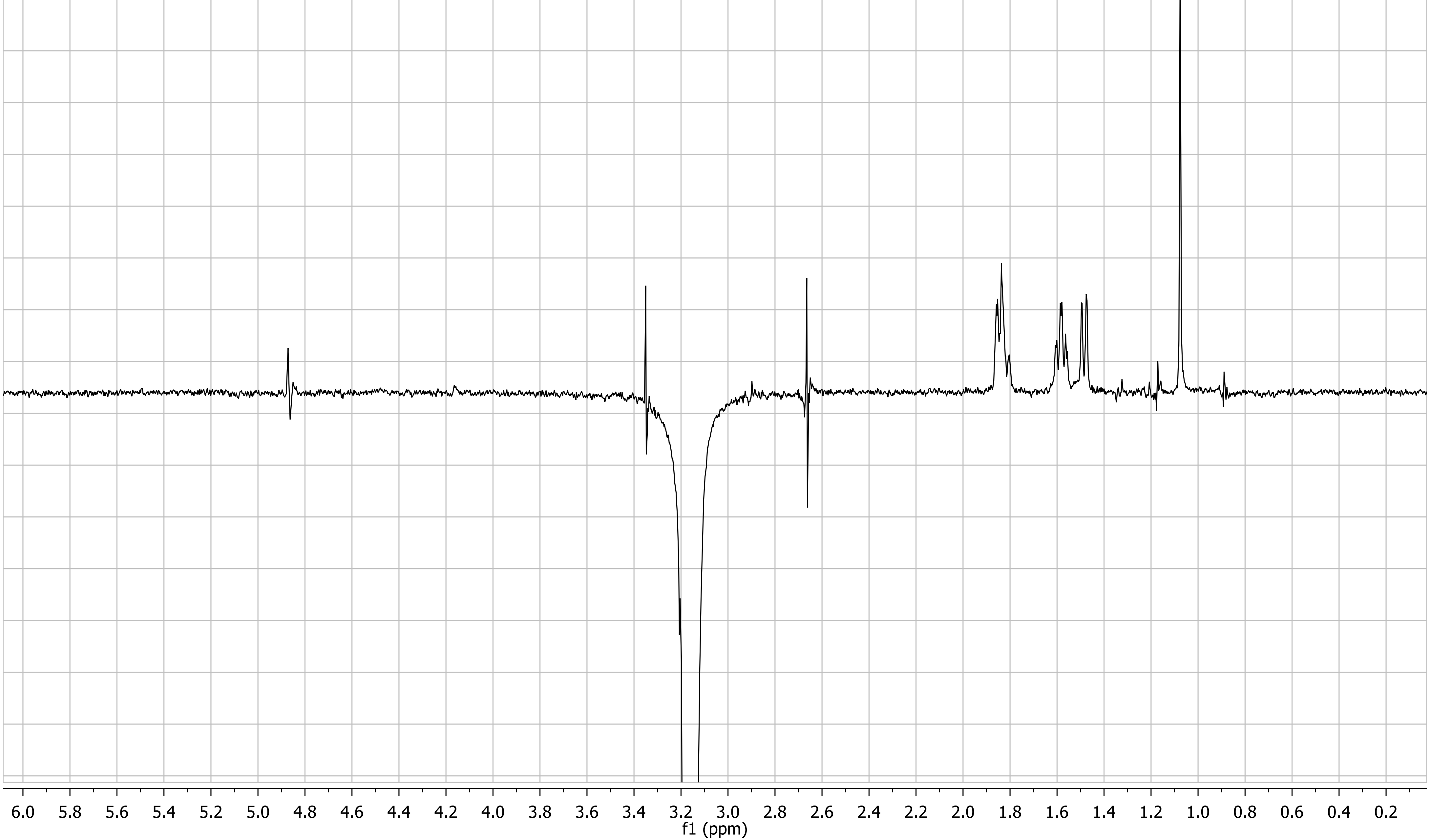

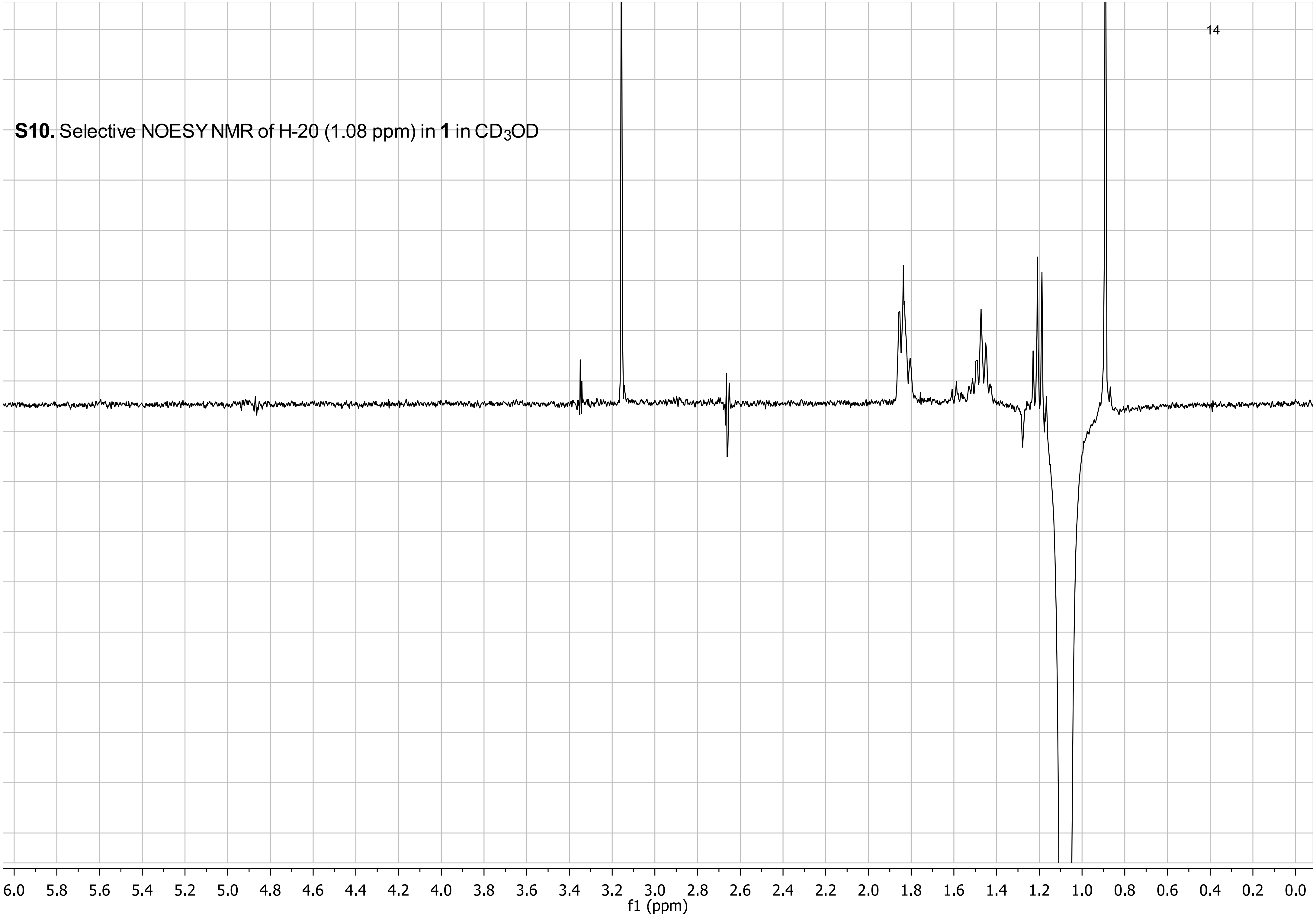

S10. Selective NOESY NMR of H-20 (1.08 ppm) in **1** in CD_3OD

S11. Selective NOESY NMR of H-19 (0.89 ppm) in **1** in CD_3OD

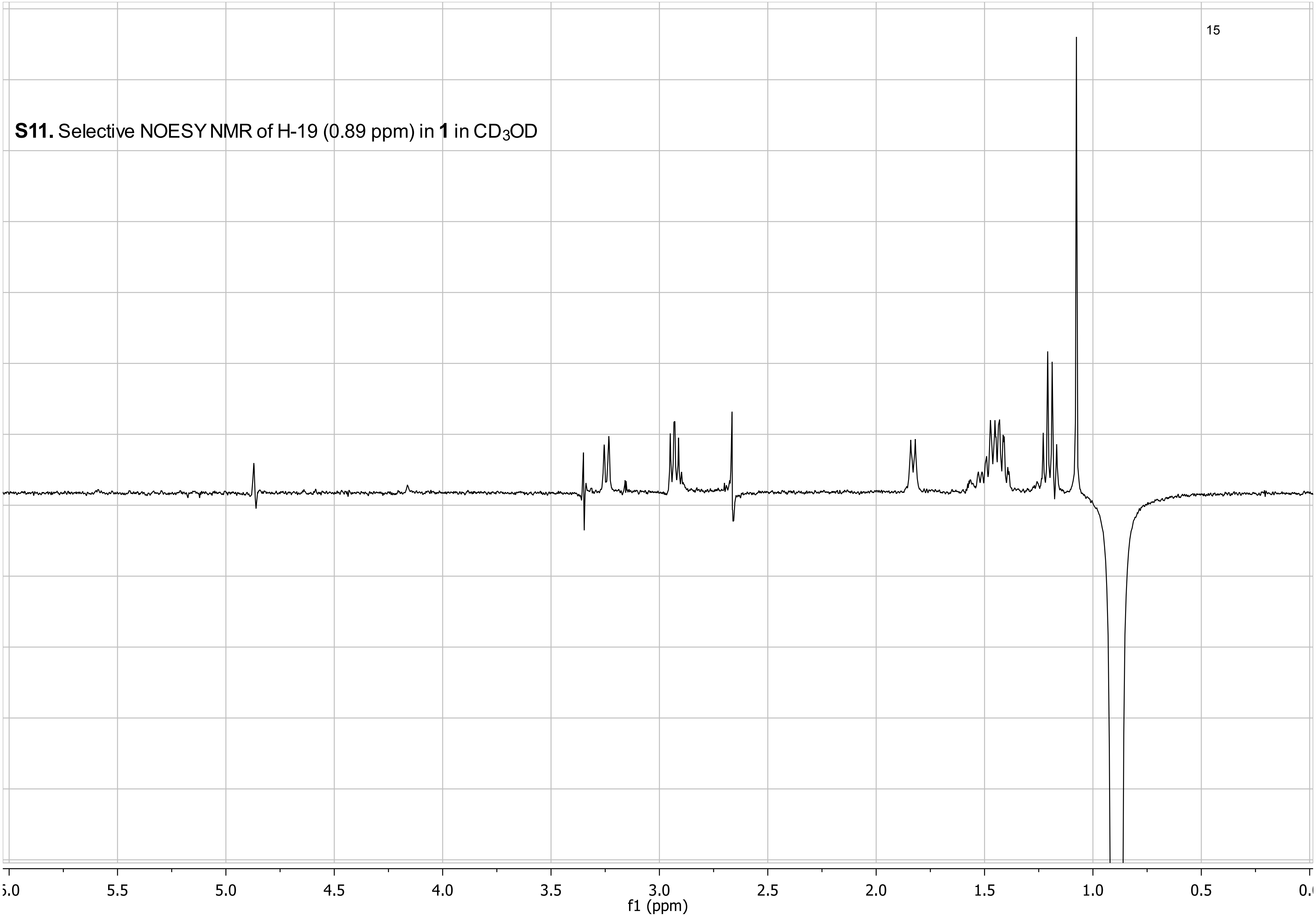

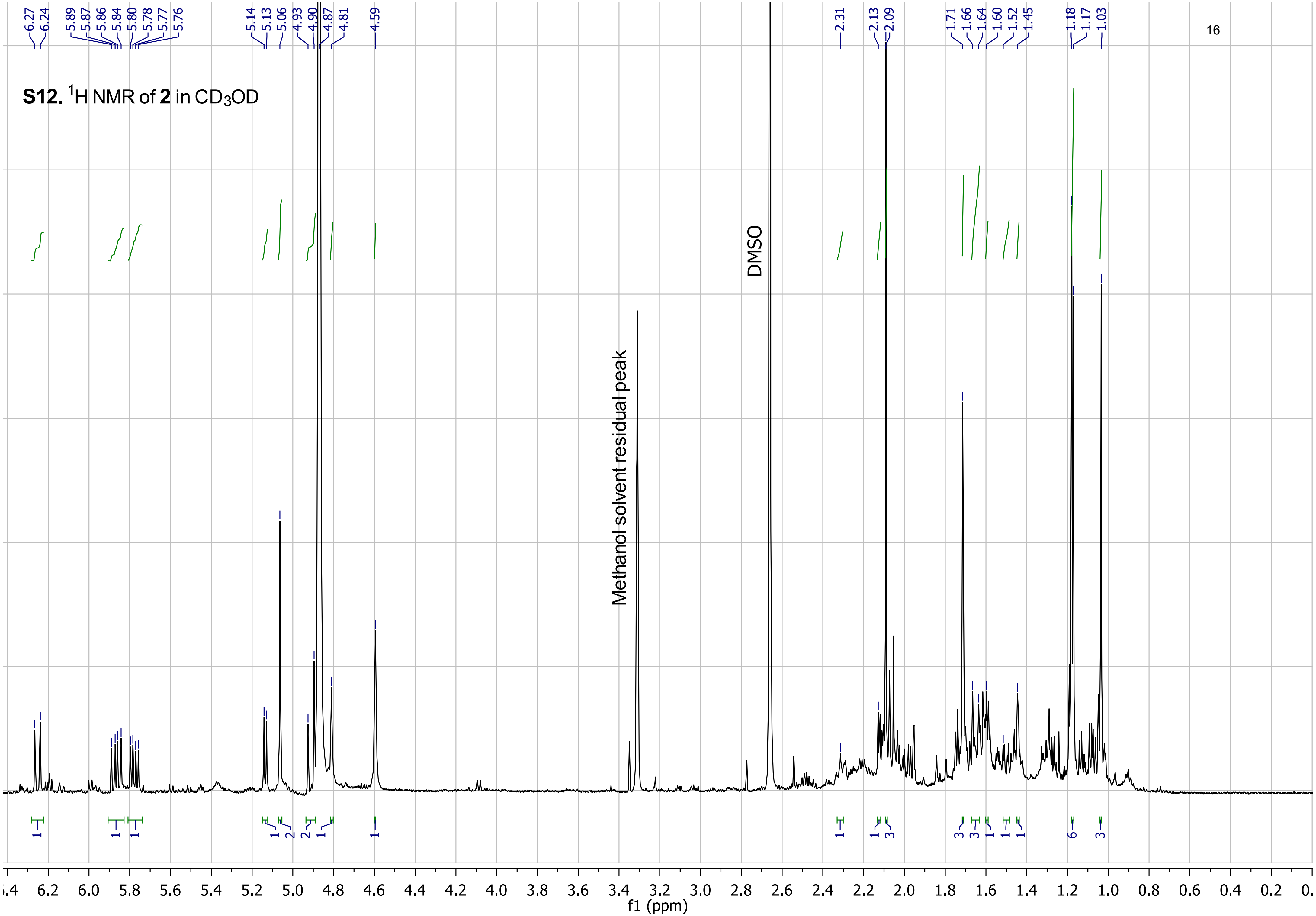
S12. ¹H NMR of 2 in CD₃OD
Methanol solvent residual peak
DMSO
f1 (ppm)

S13. ^{13}C NMR of **2** in CD_3OD

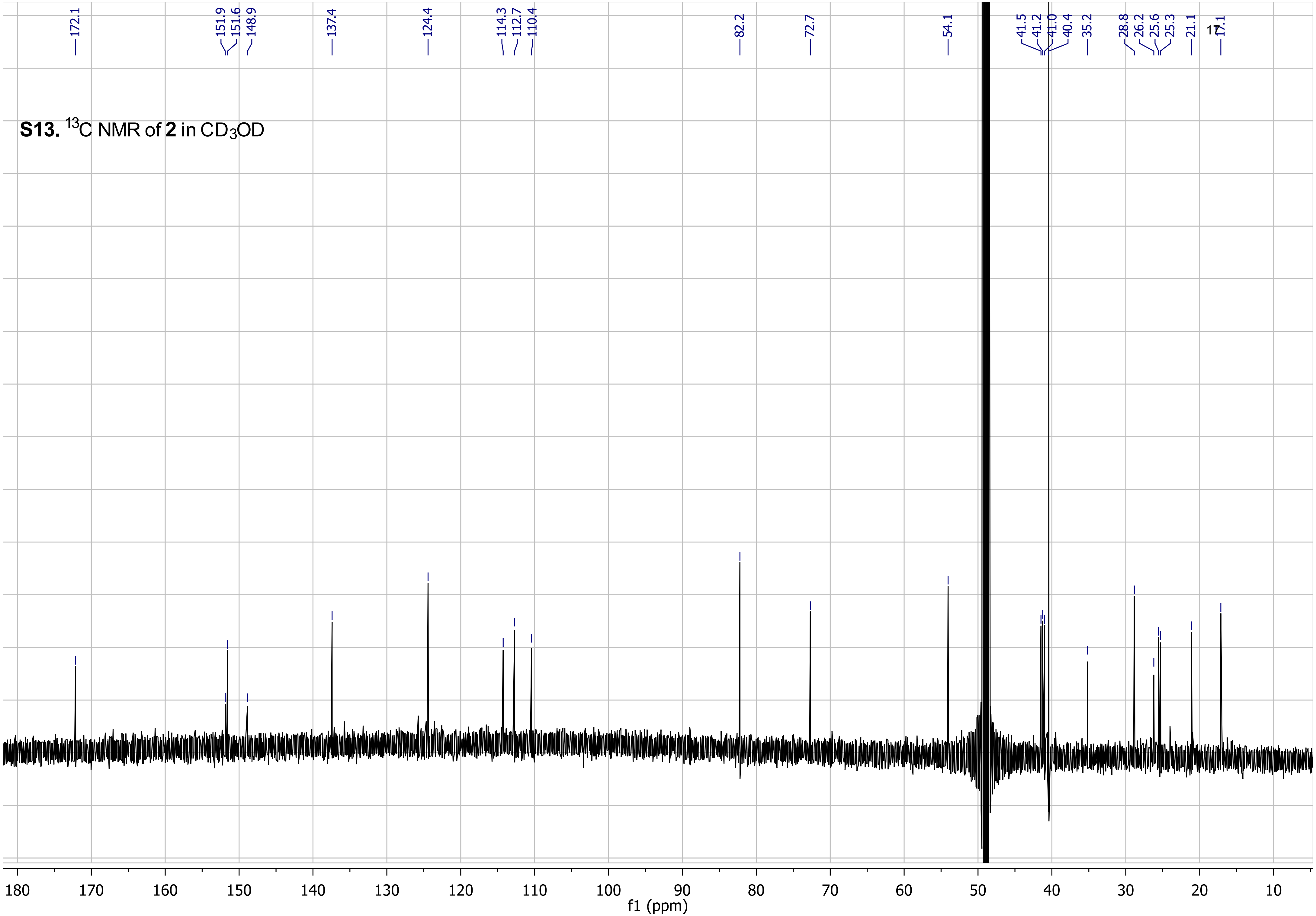

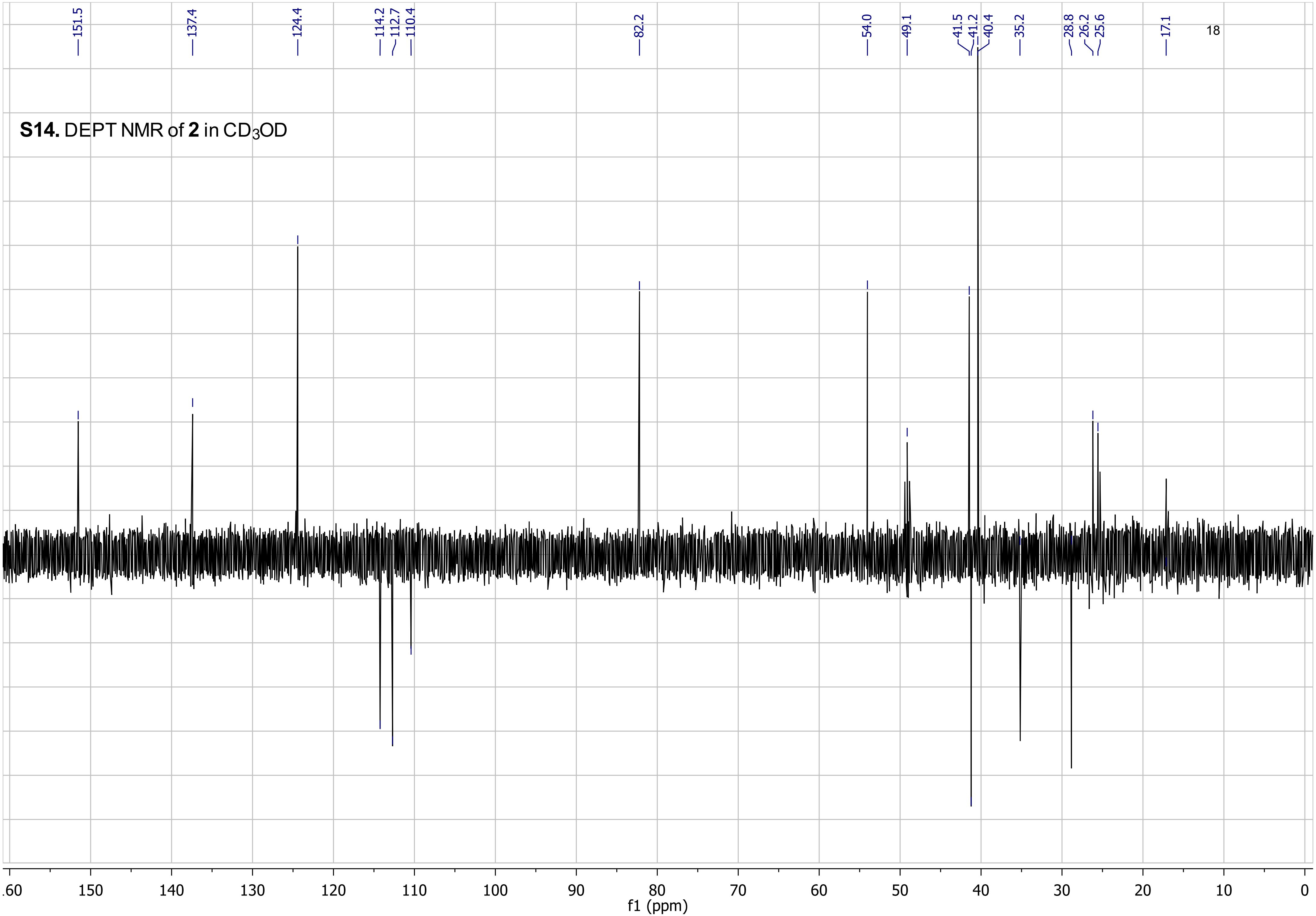

S14. DEPT NMR of 2 in CD3OD
151.5
137.4
124.4
114.2
112.7
110.4
82.2
54.0
49.1
41.5
41.2
40.4
35.2
28.8
26.2
25.6
17.1
f1 (ppm)

S15. COSY NMR of **2** in CD_3OD

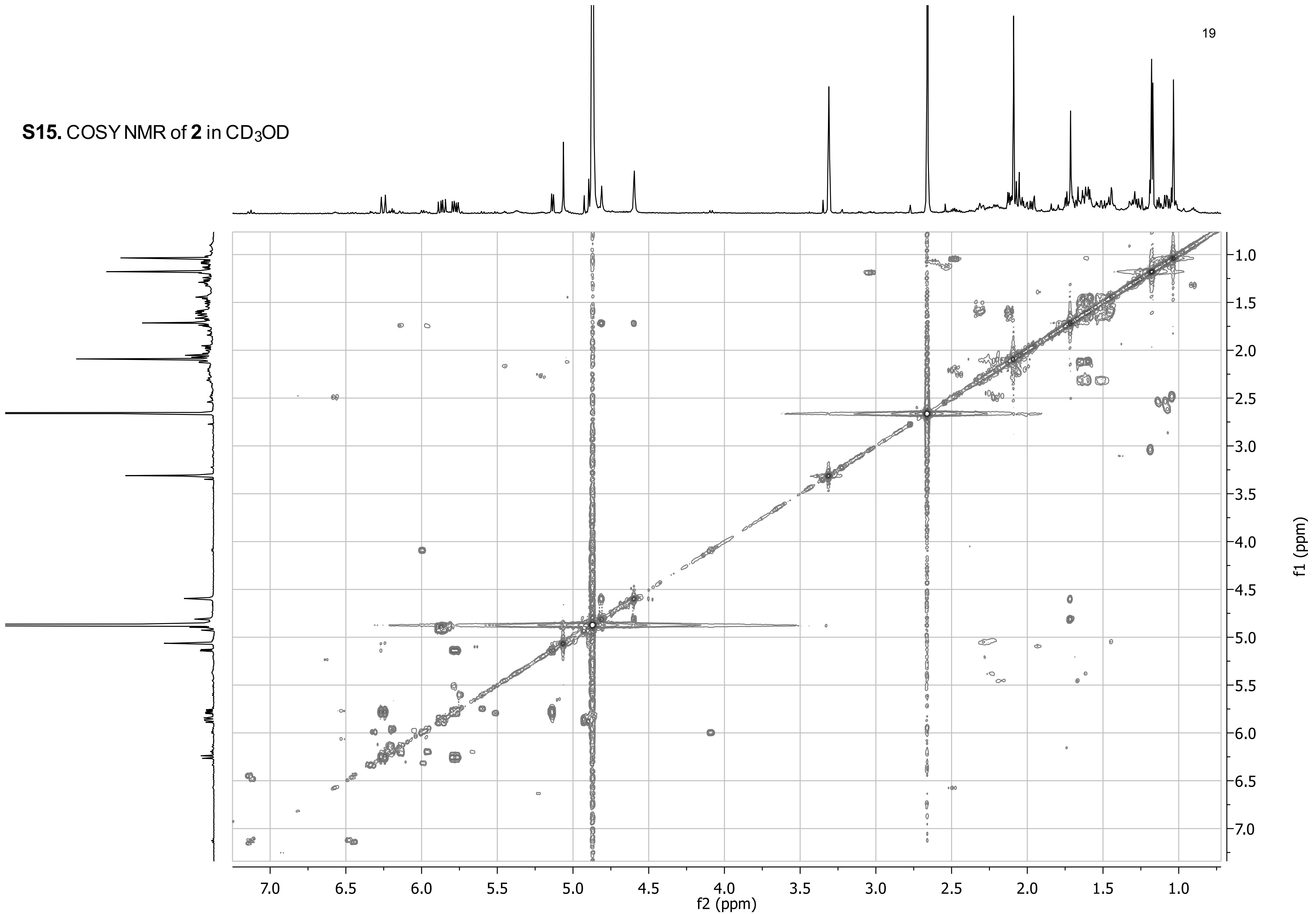

S16. HSQC NMR of **2** in CD_3OD

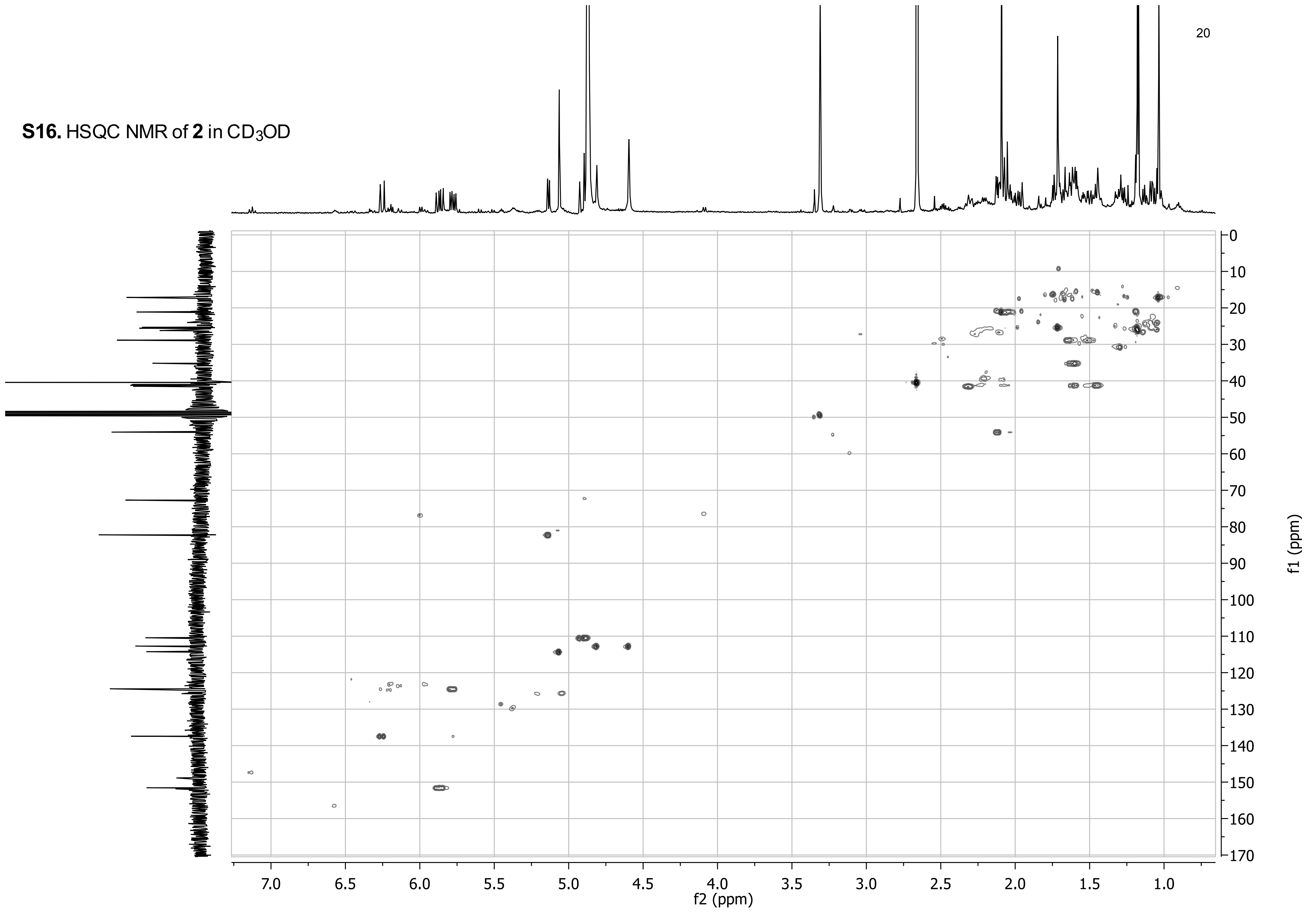

S17. HMBC NMR of **2** in CD_3OD

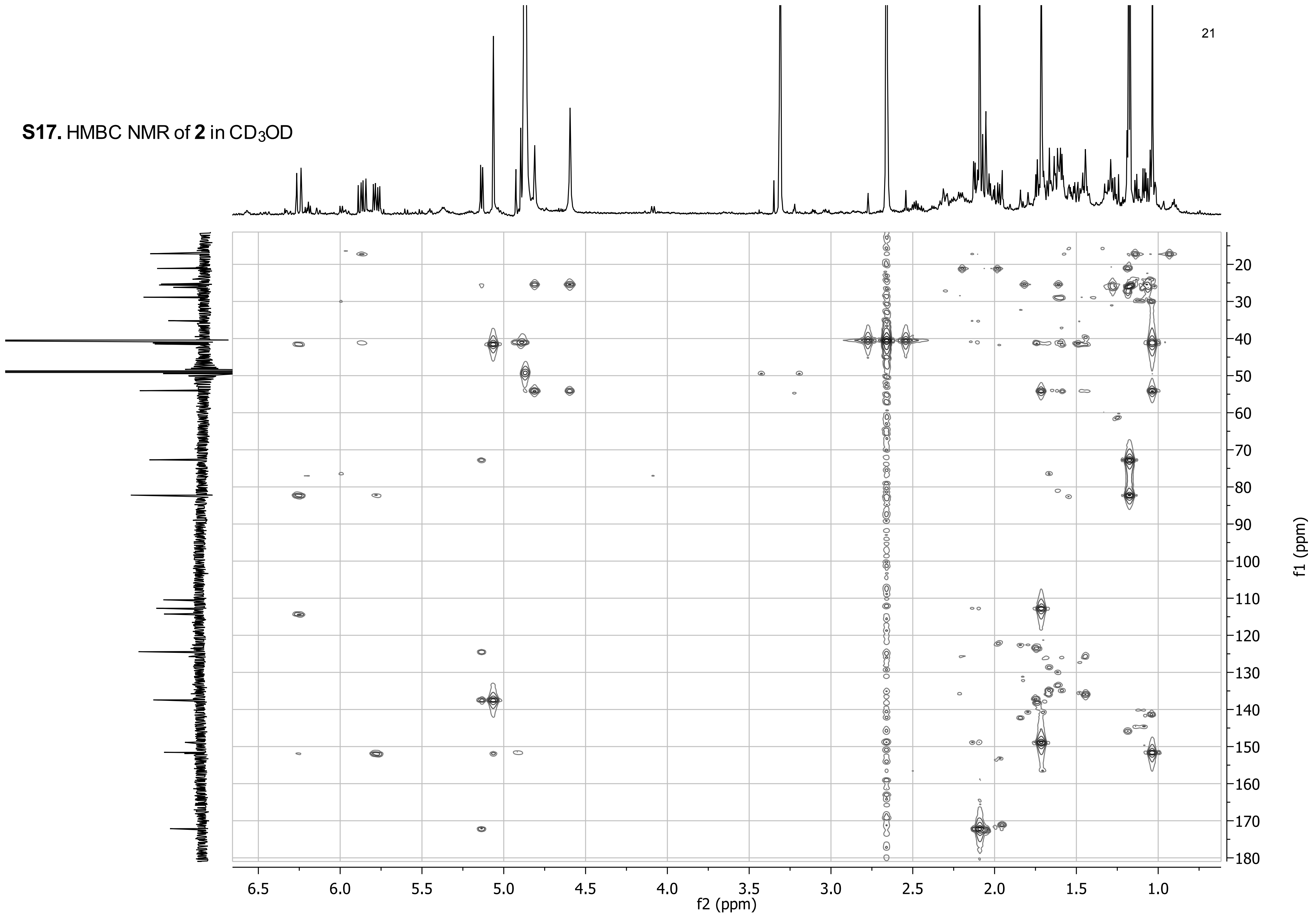

S18. Selective NOESY NMR of H-17 (5.14 ppm) in **2** in CD_3OD

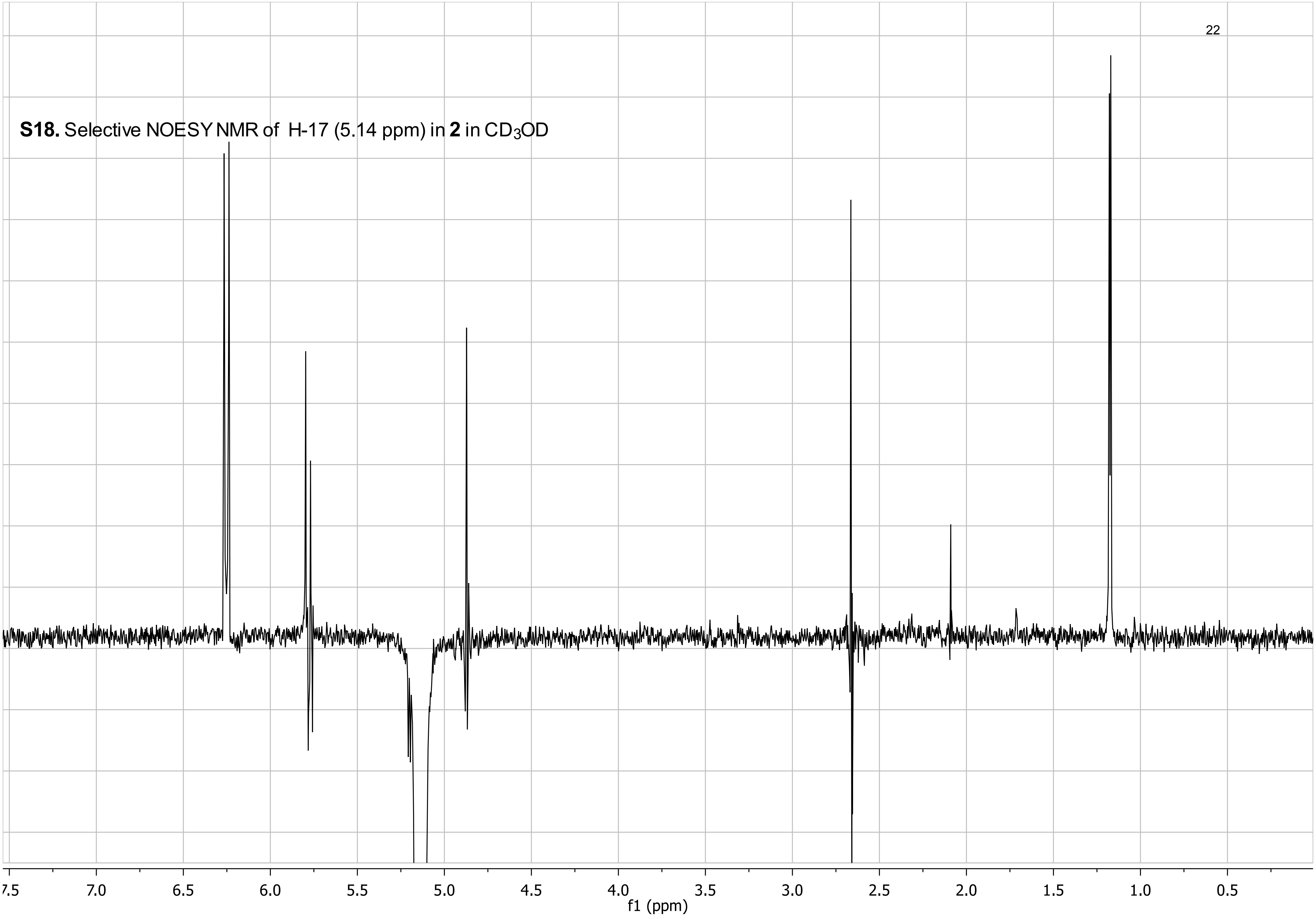

S19. Selective NOESY NMR of H-4 (2.31 ppm) in **2** in CD_3OD

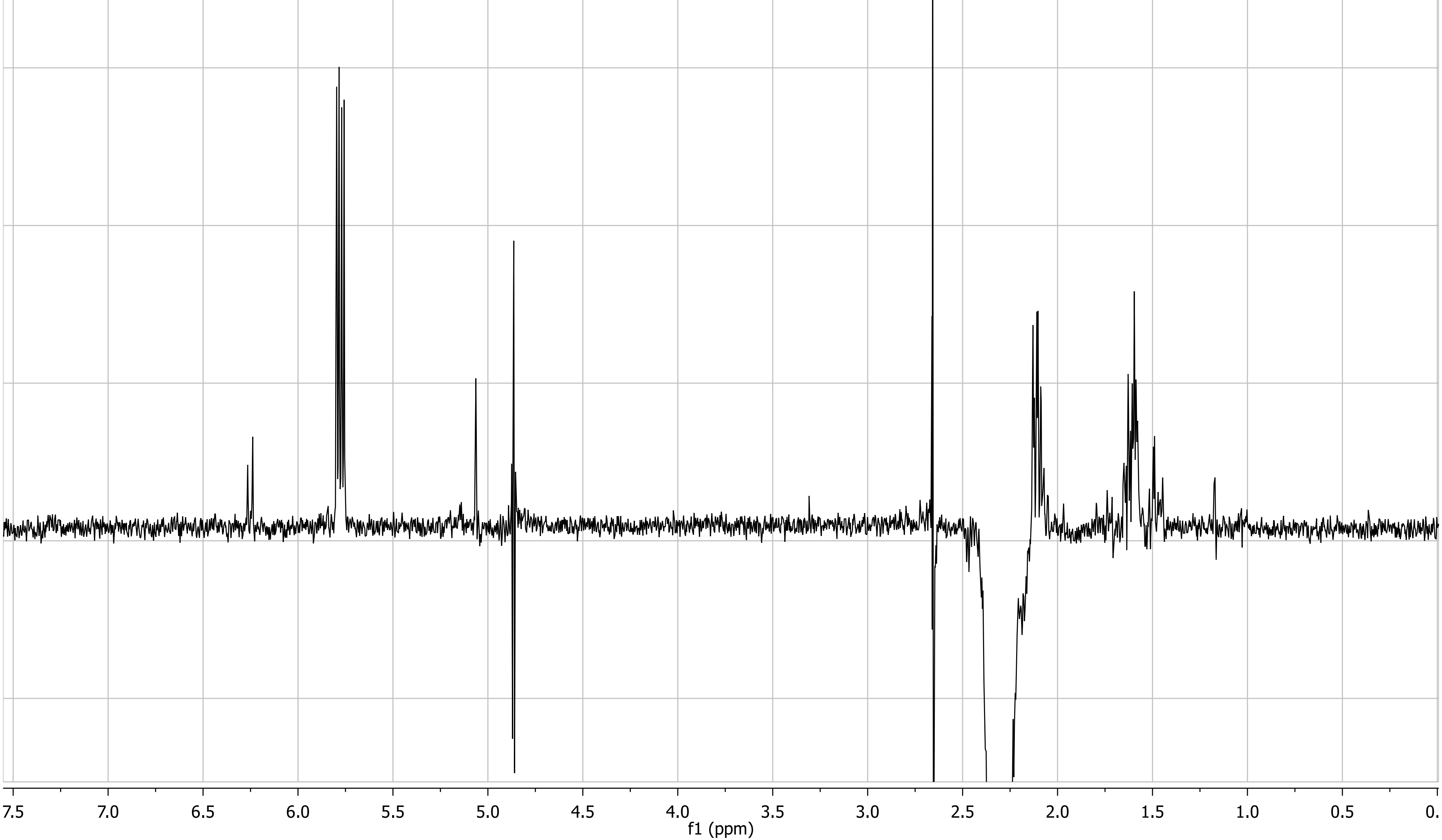

S20. Selective NOESY NMR of OAcMe (2.09 ppm) in **2** in CD_3OD

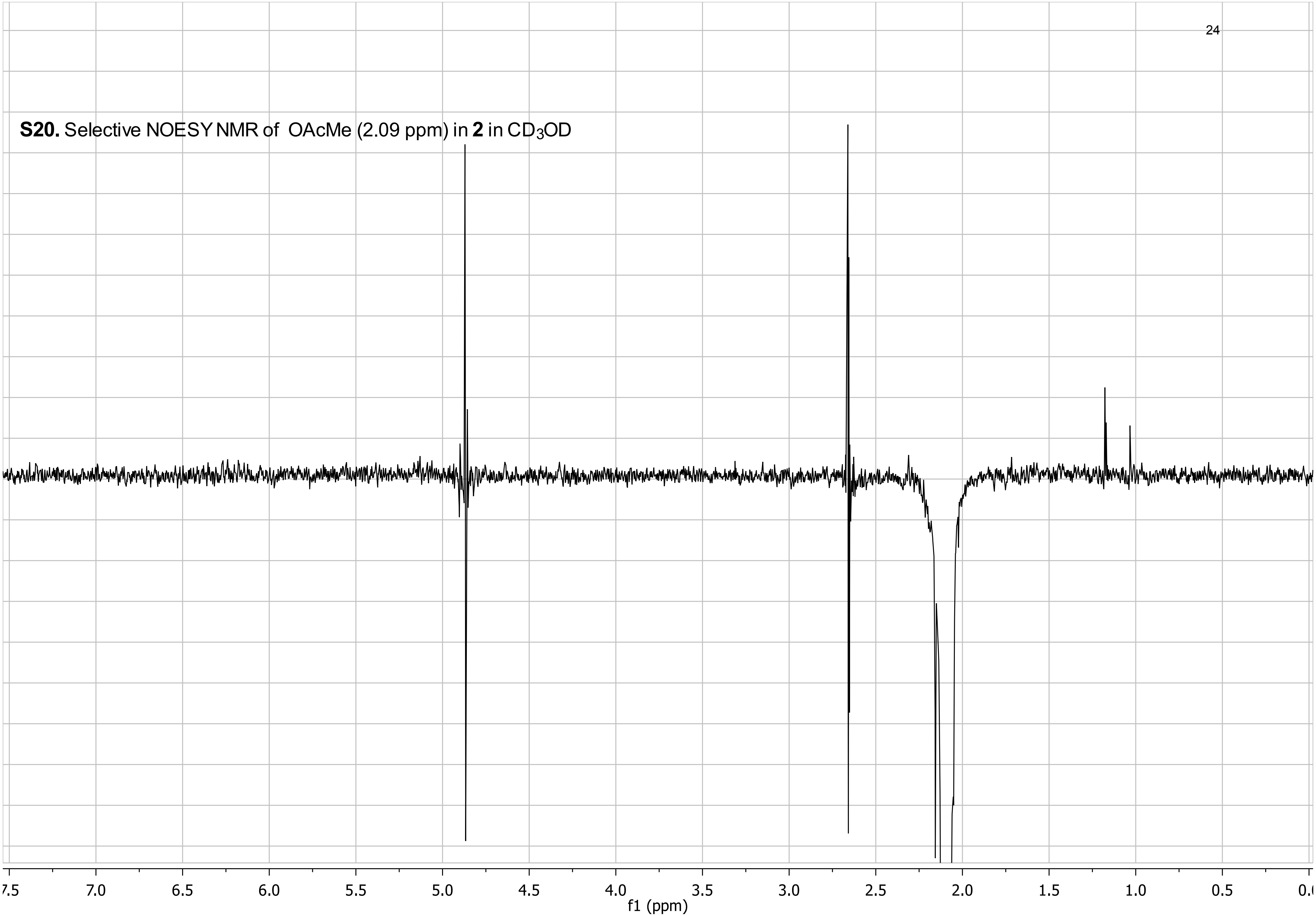

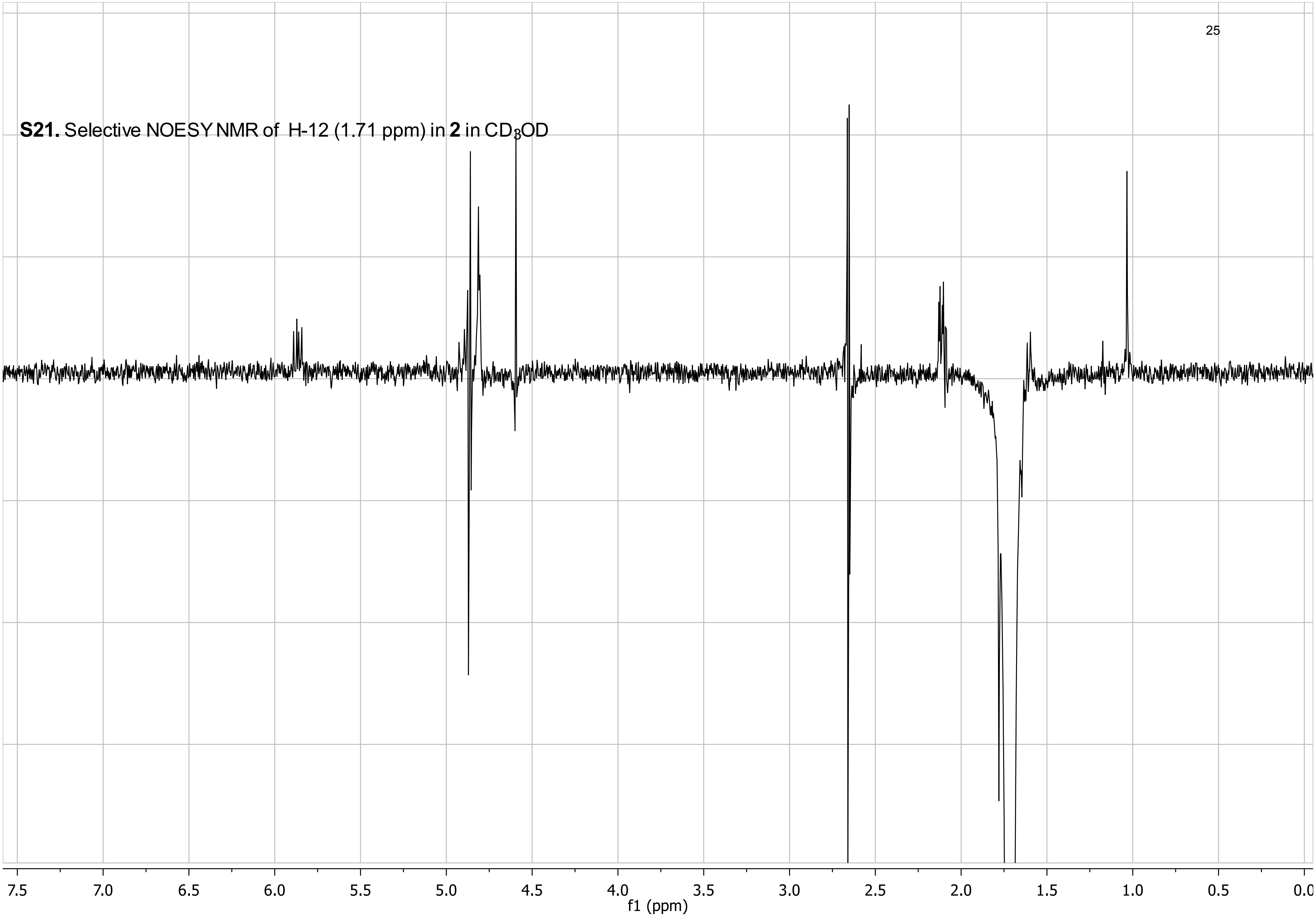

S21. Selective NOESY NMR of H-12 (1.71 ppm) in **2** in CD_3OD

S22. Selective NOESY NMR of H-7 (1.03 ppm) in **2** in CD_3OD

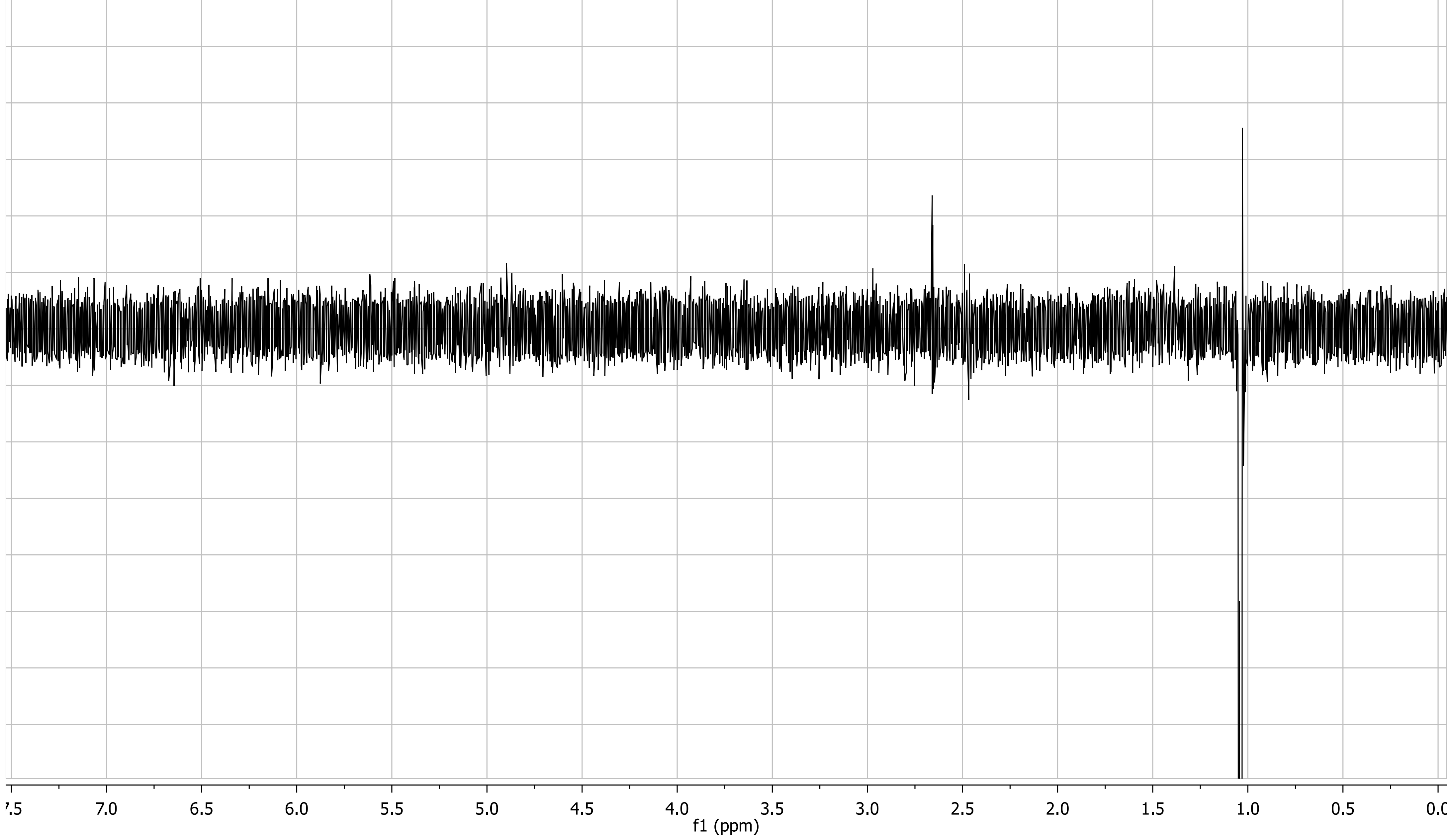

Scheme 1: Structures of known compounds isolated from *Sinularia* sp.

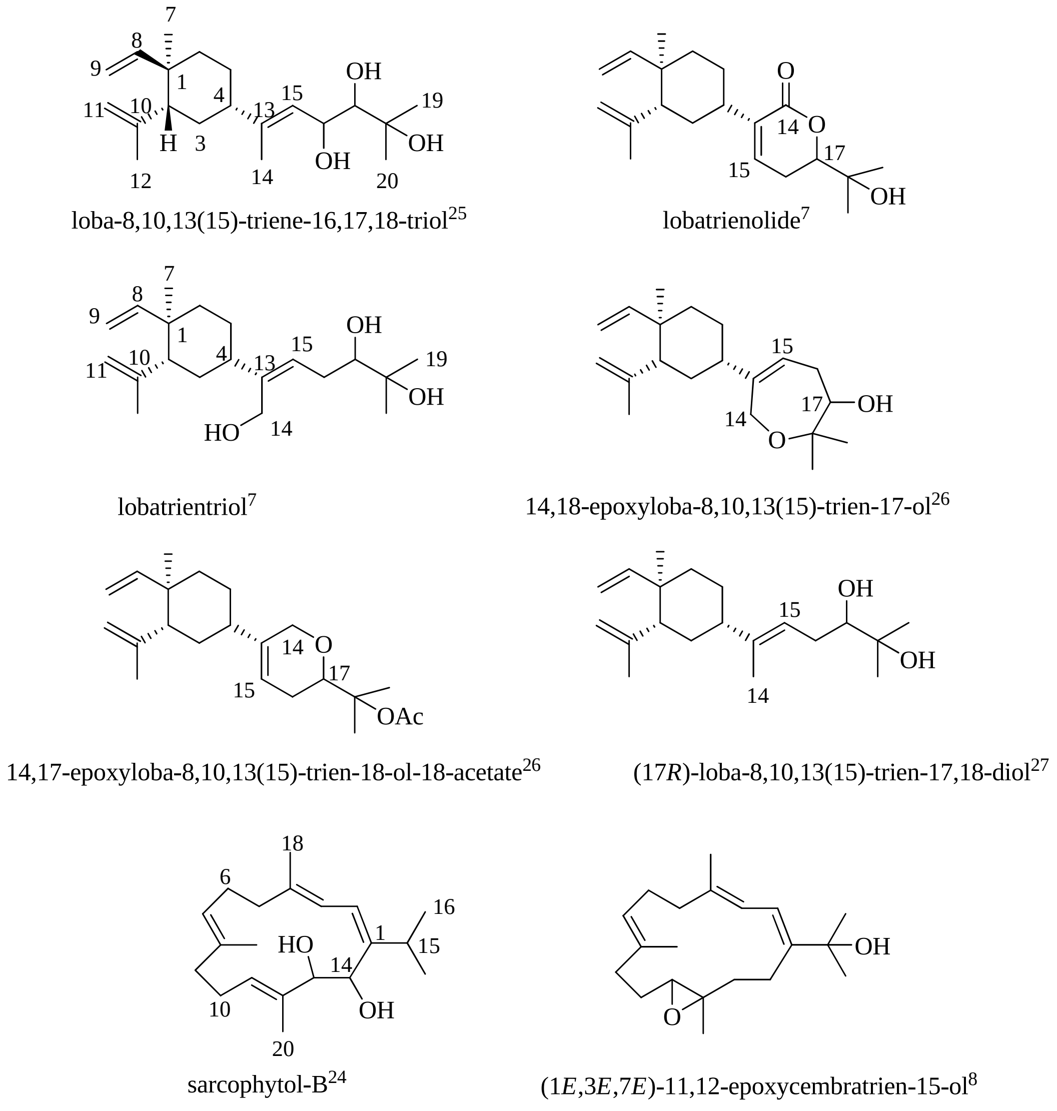

S23. ^{1}H NMR of loba-8,10,13(15)-triene-16,17,18-triol in CD_3OD

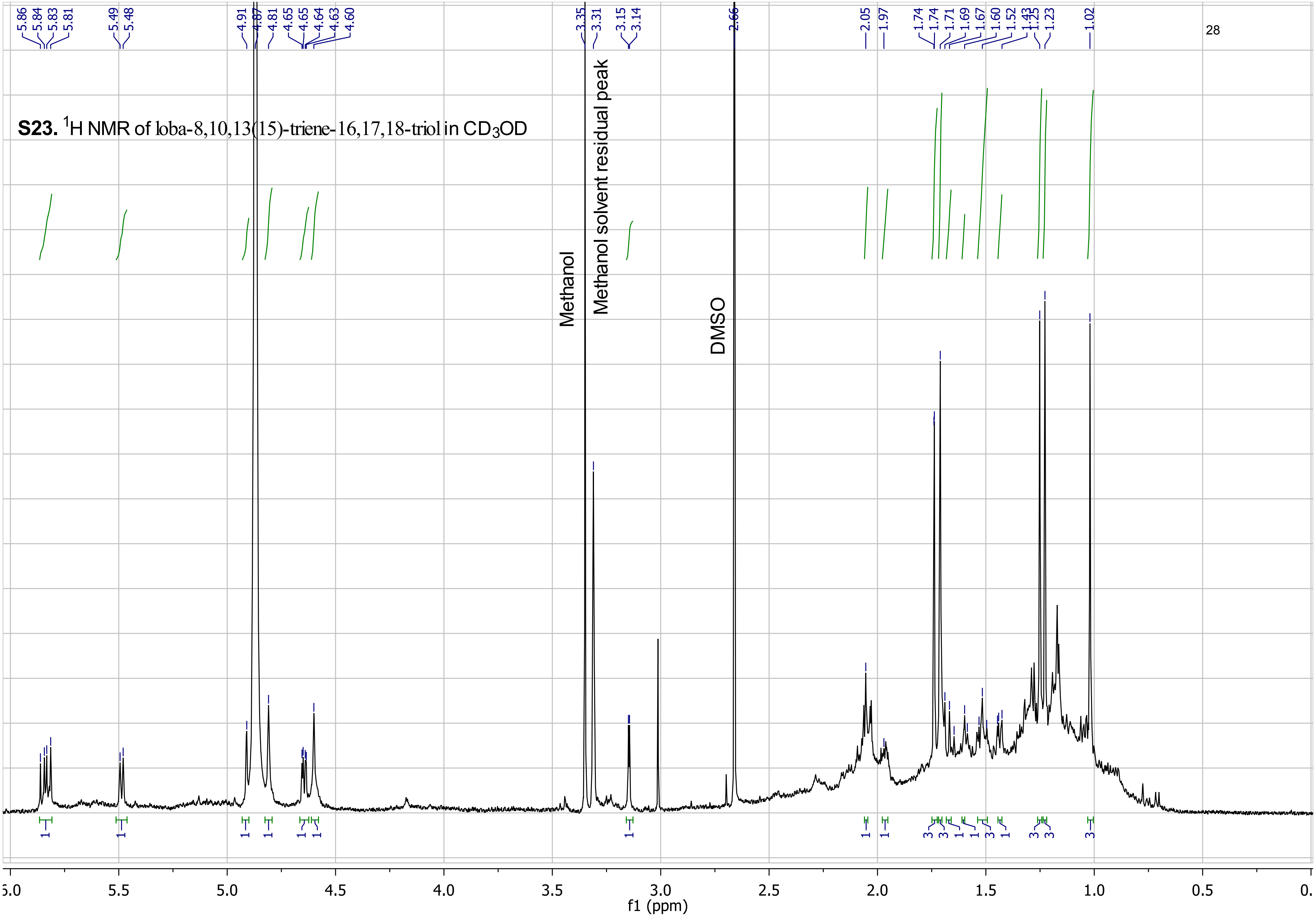

S24. ^{13}C NMR of loba-8,10,13(15)-triene-16,17,18-triol in CD_3OD

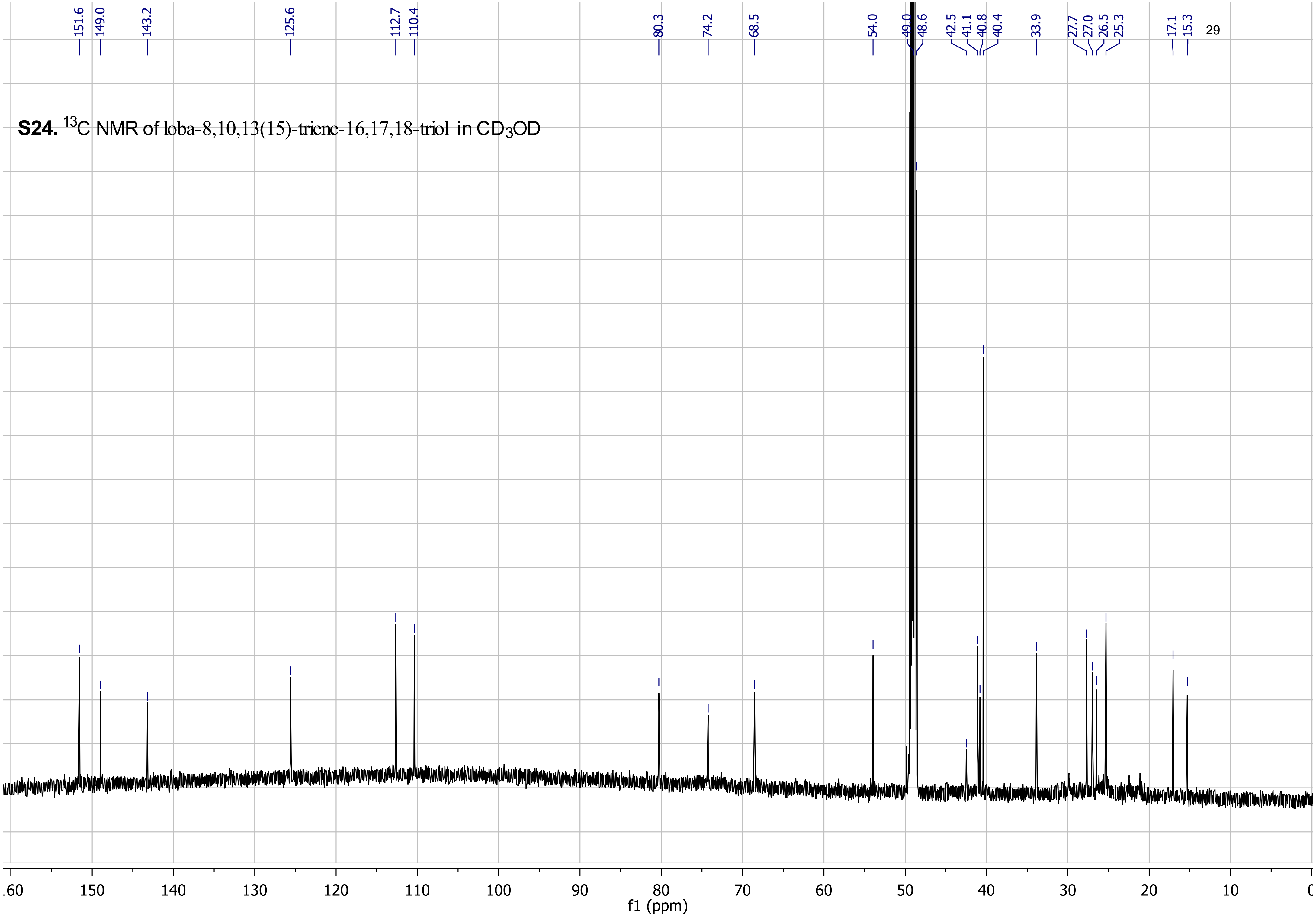

S25. DEPT NMR of loba-8,10,13(15)-triene-16,17,18-triol in CD_3OD

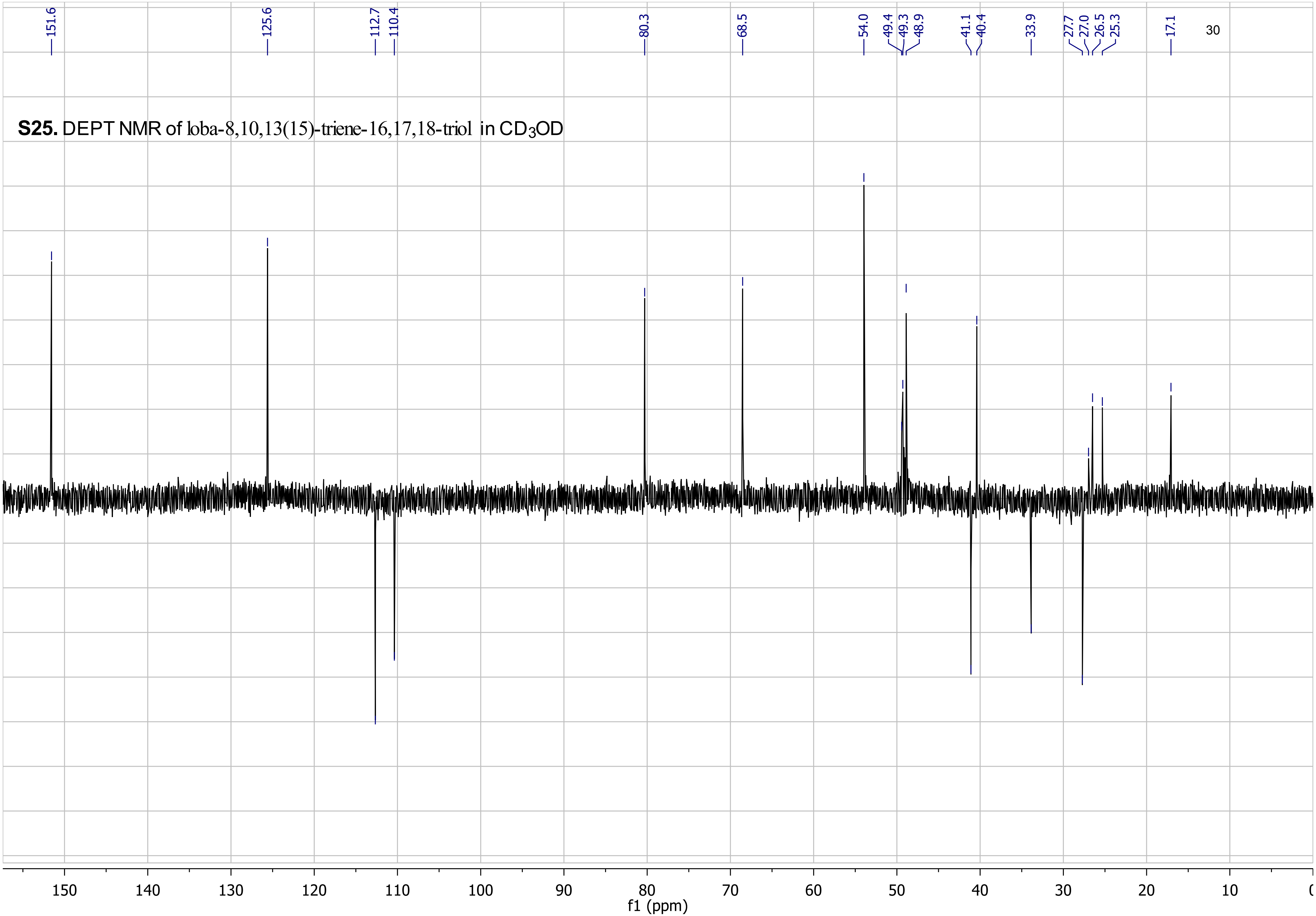

S26. COSY NMR of loba-8,10,13(15)-triene-16,17,18-triol in CD_3OD

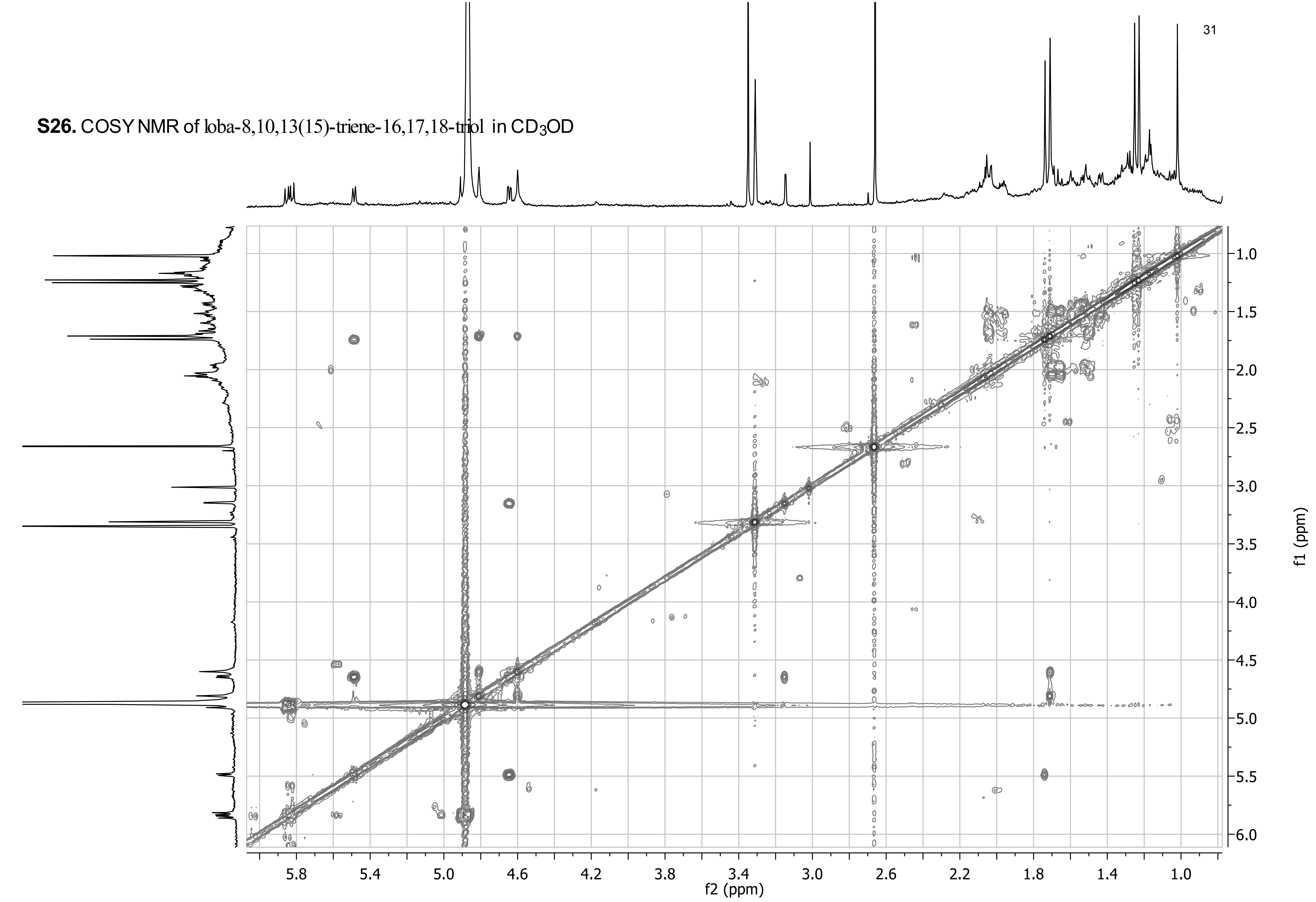

S27. HSQC NMR of loba-8,10,13(15)-triene-16,17,18-triol in CD_3OD

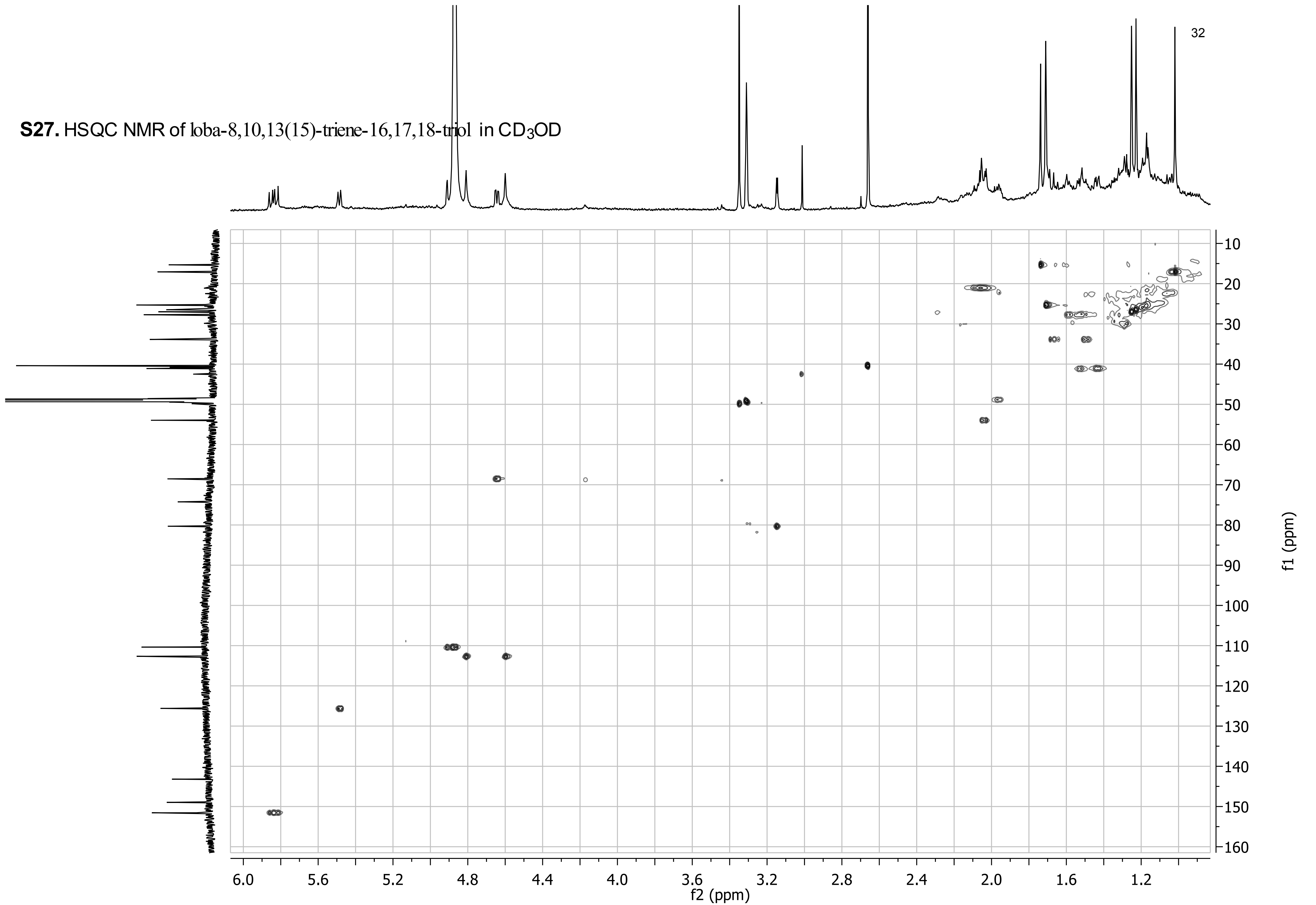

S28. HMBC NMR of loba-8,10,13(15)-triene-16,17,18-triol in CD_3OD

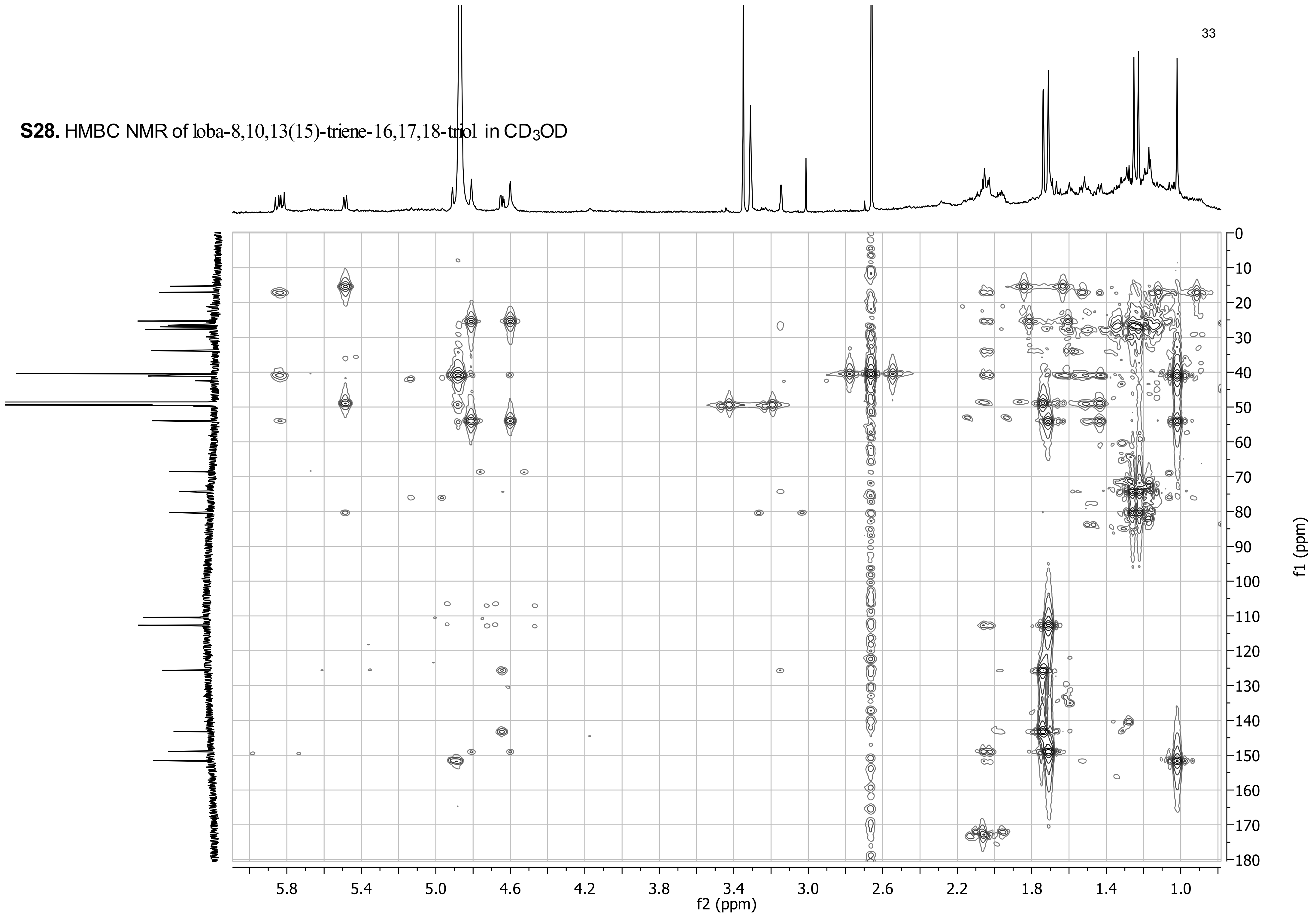

S29. Selective NOESY NMR of H-16 (4.65 ppm) in loba-8,10,13(15)-triene-16,17,18-triol in CD_3OD

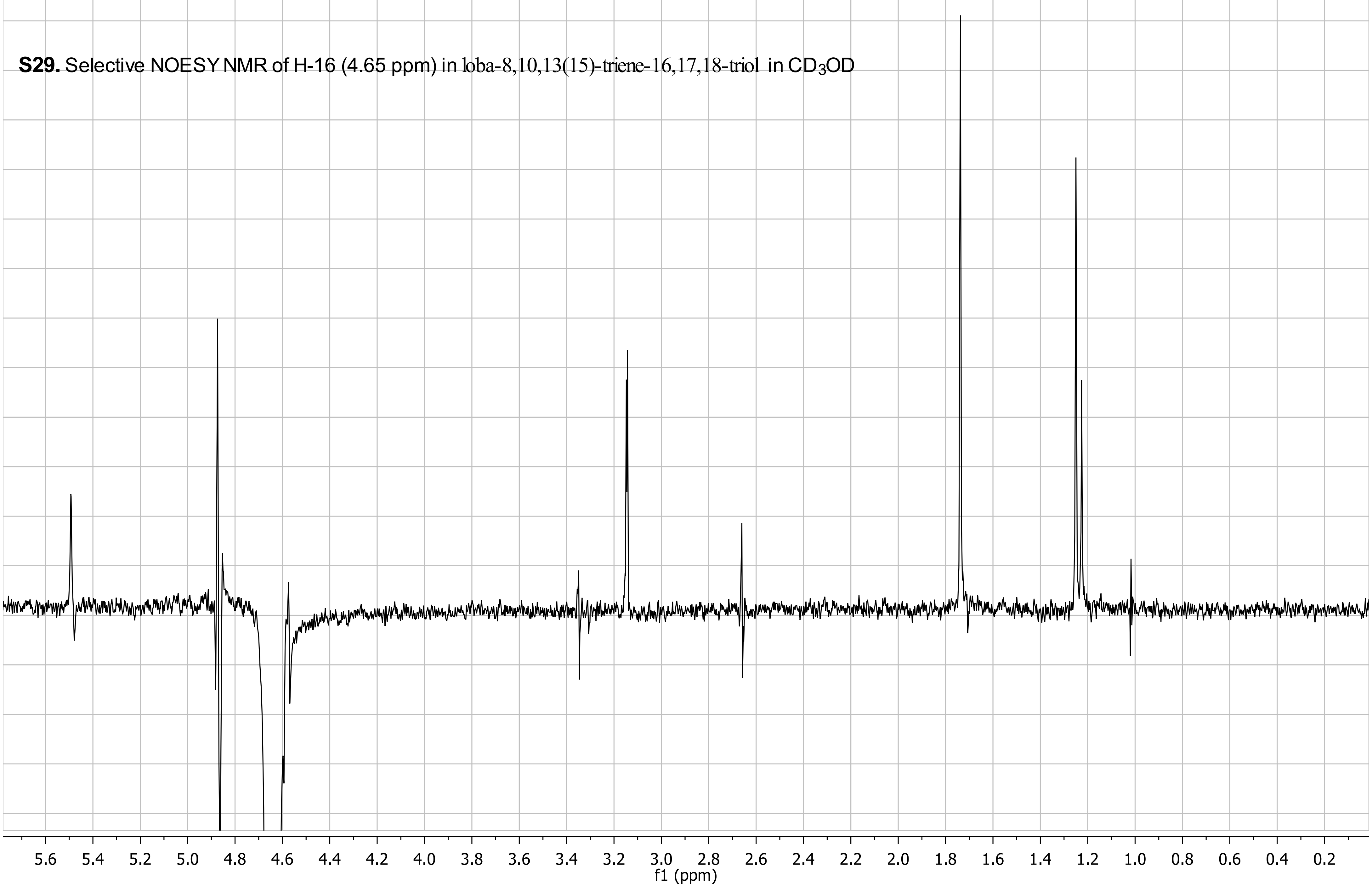

S30. Selective NOESY NMR of H-17 (3.15 ppm) in loba-8,10,13(15)-triene-16,17,18-triol in CD_3OD

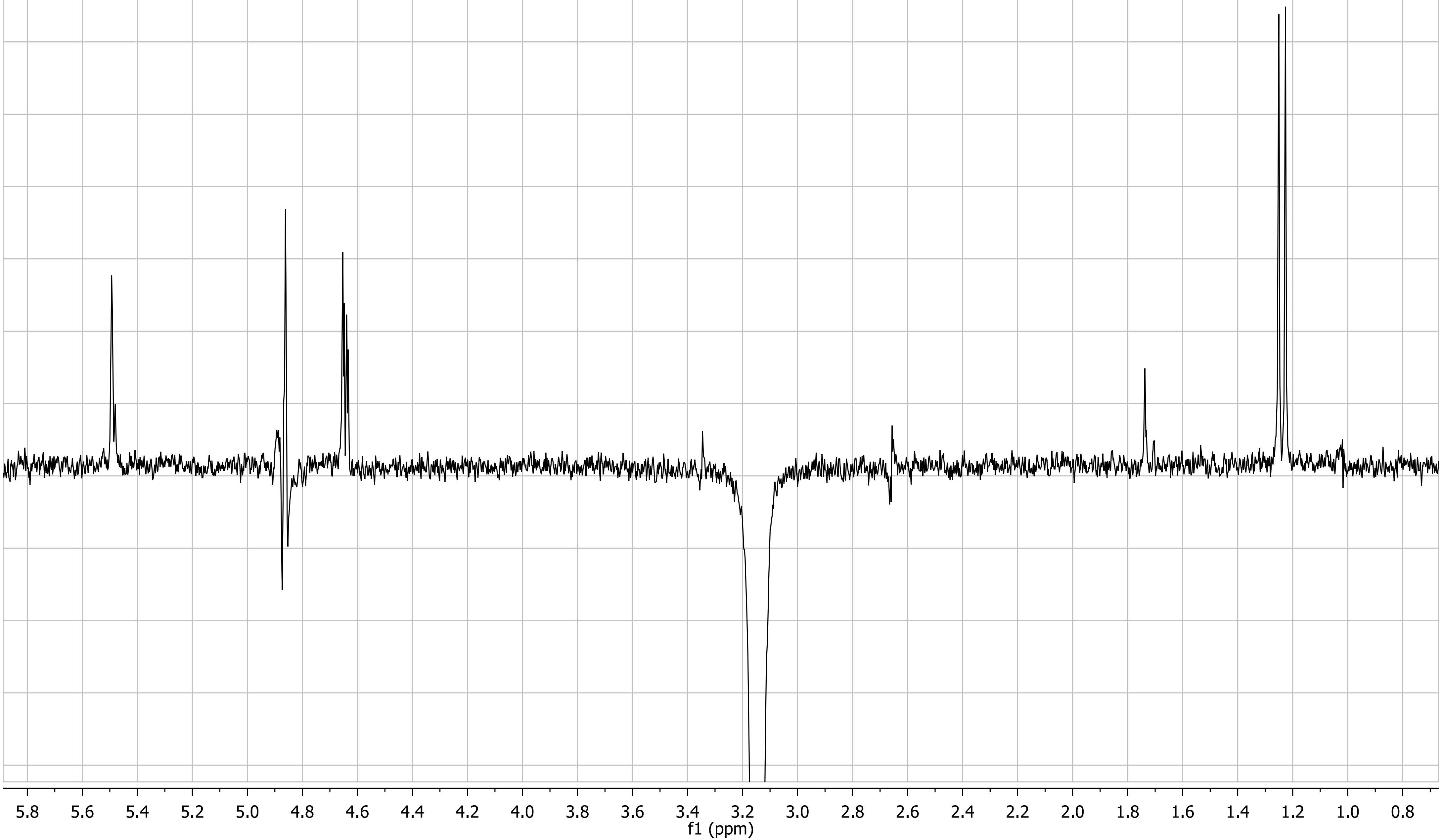

S31. Selective NOESY NMR of H-14 (1.74 ppm) in loba-8,10,13(15)-triene-16,17,18-triol in CD_3OD

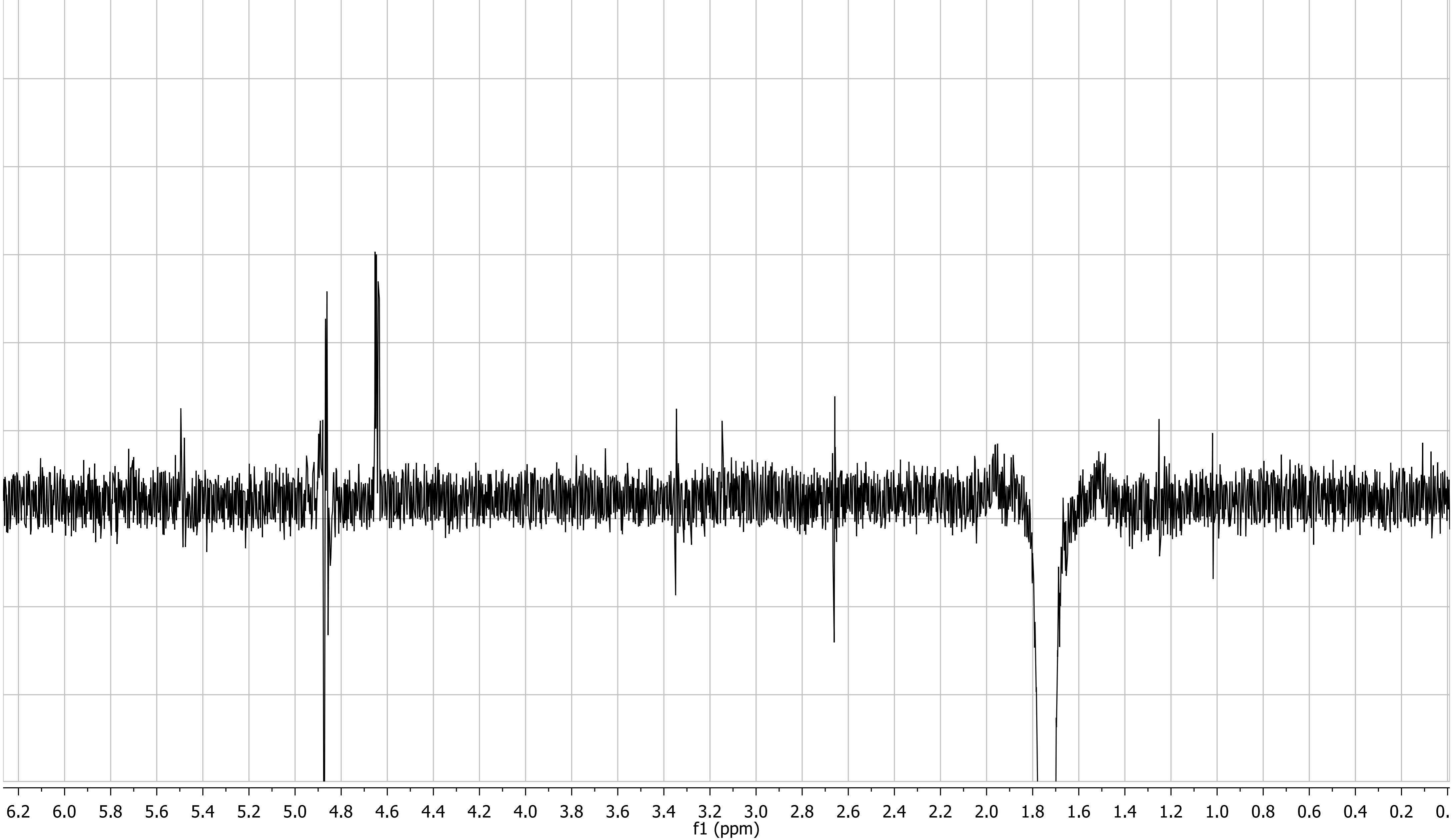

S32. Selective NOESY NMR of H-12 (1.71 ppm) in loba-8,10,13(15)-triene-16,17,18-triol in CD_3OD

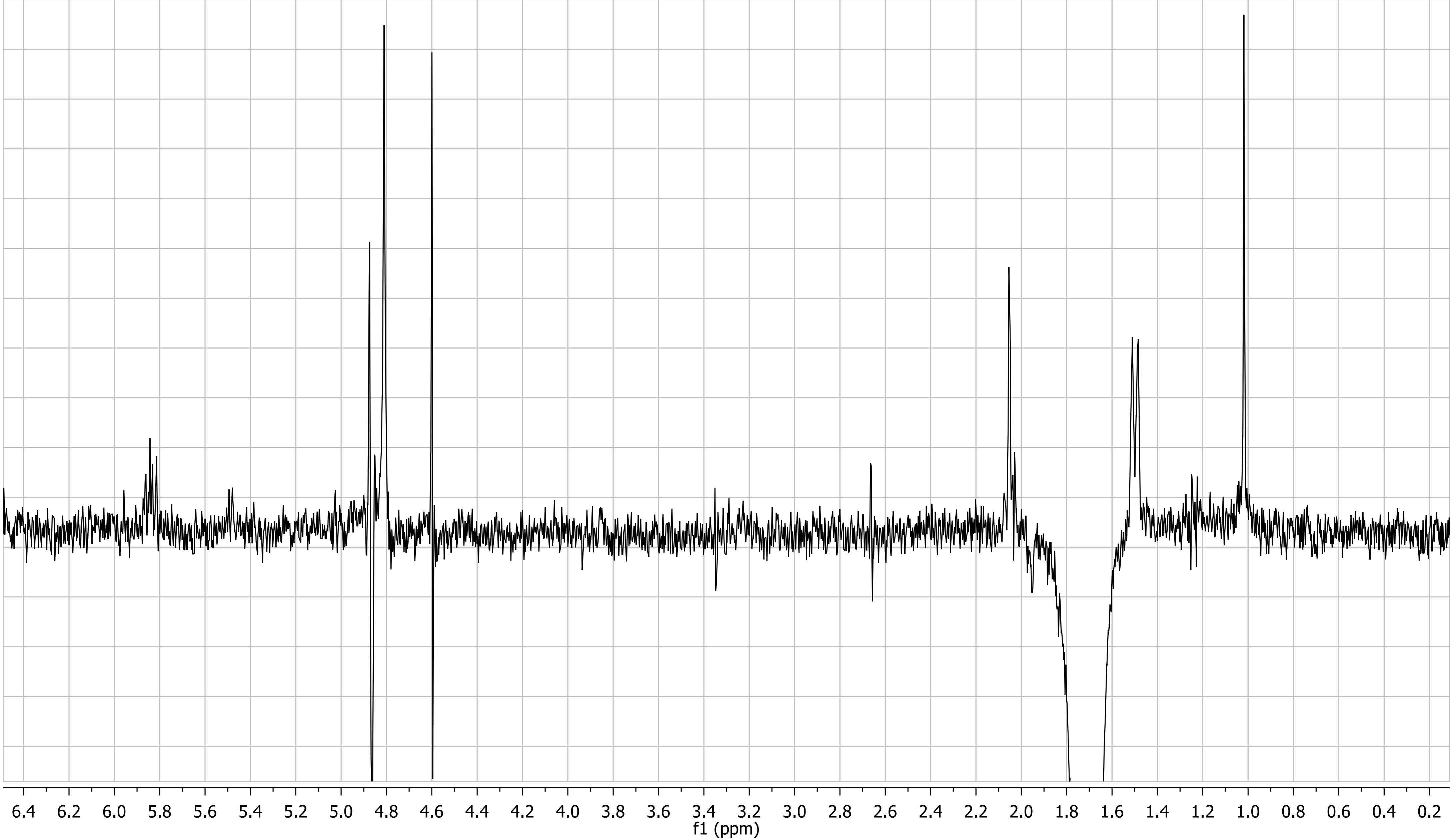

S33. Selective NOESY NMR of H-20 (1.25 ppm) in loba-8,10,13(15)-triene-16,17,18-triol in CD_3OD

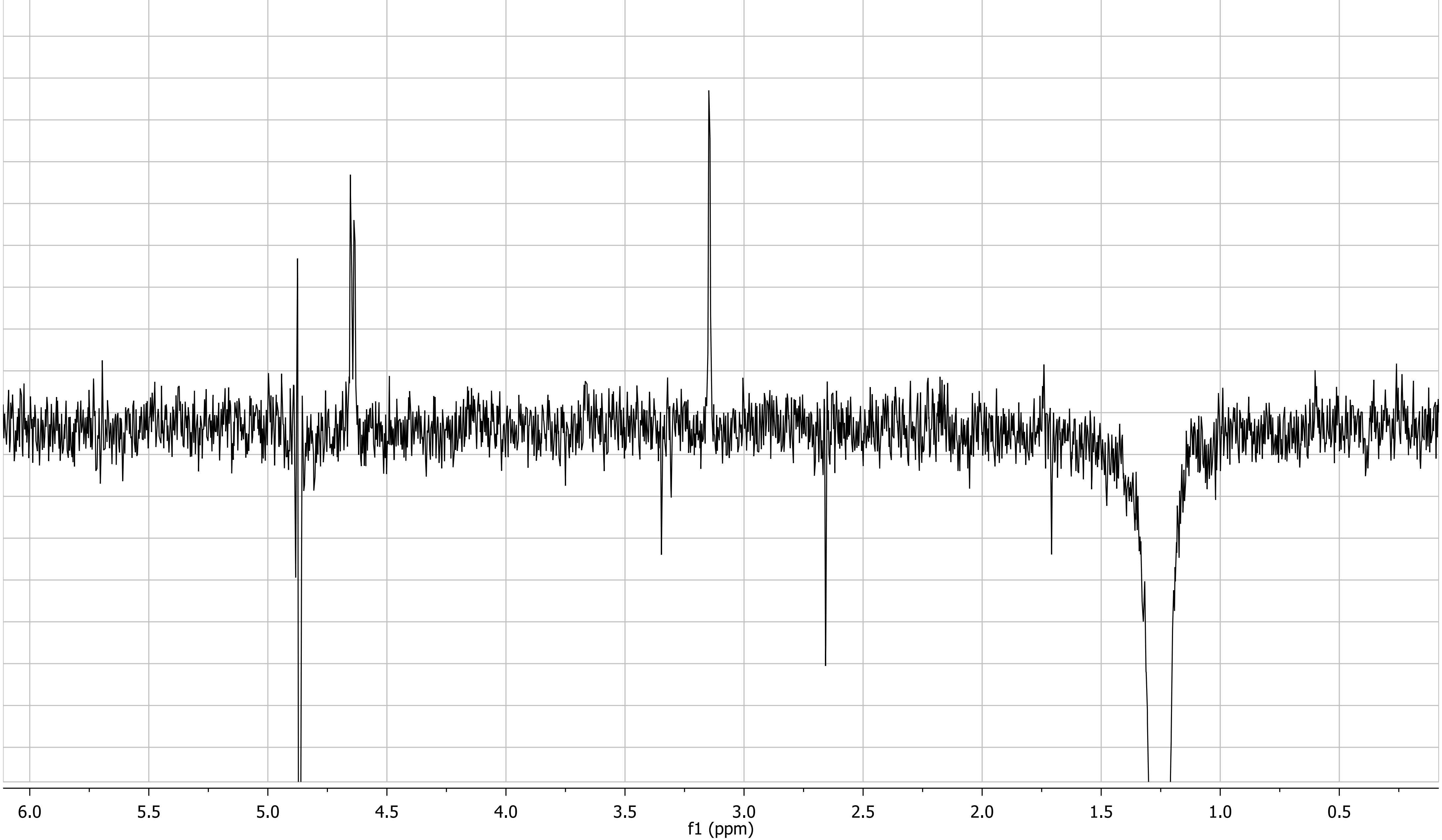

S34. Selective NOESY NMR of H-19 (1.23 ppm) in loba-8,10,13(15)-triene-16,17,18-triol in CD_3OD

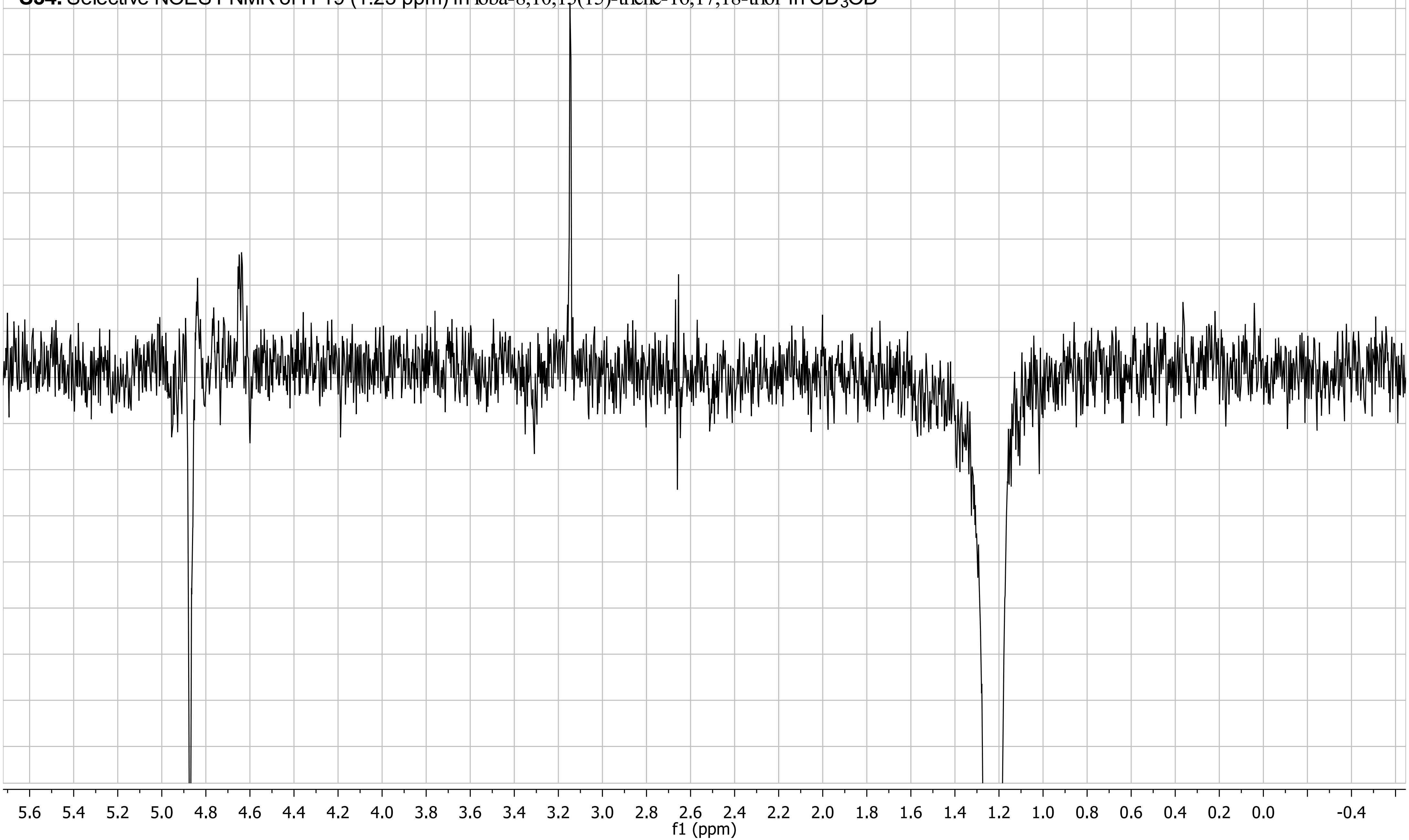

S35. Selective NOESY NMR of H-7 (1.02 ppm) in loba-8,10,13(15)-triene-16,17,18-triol in CD_3OD

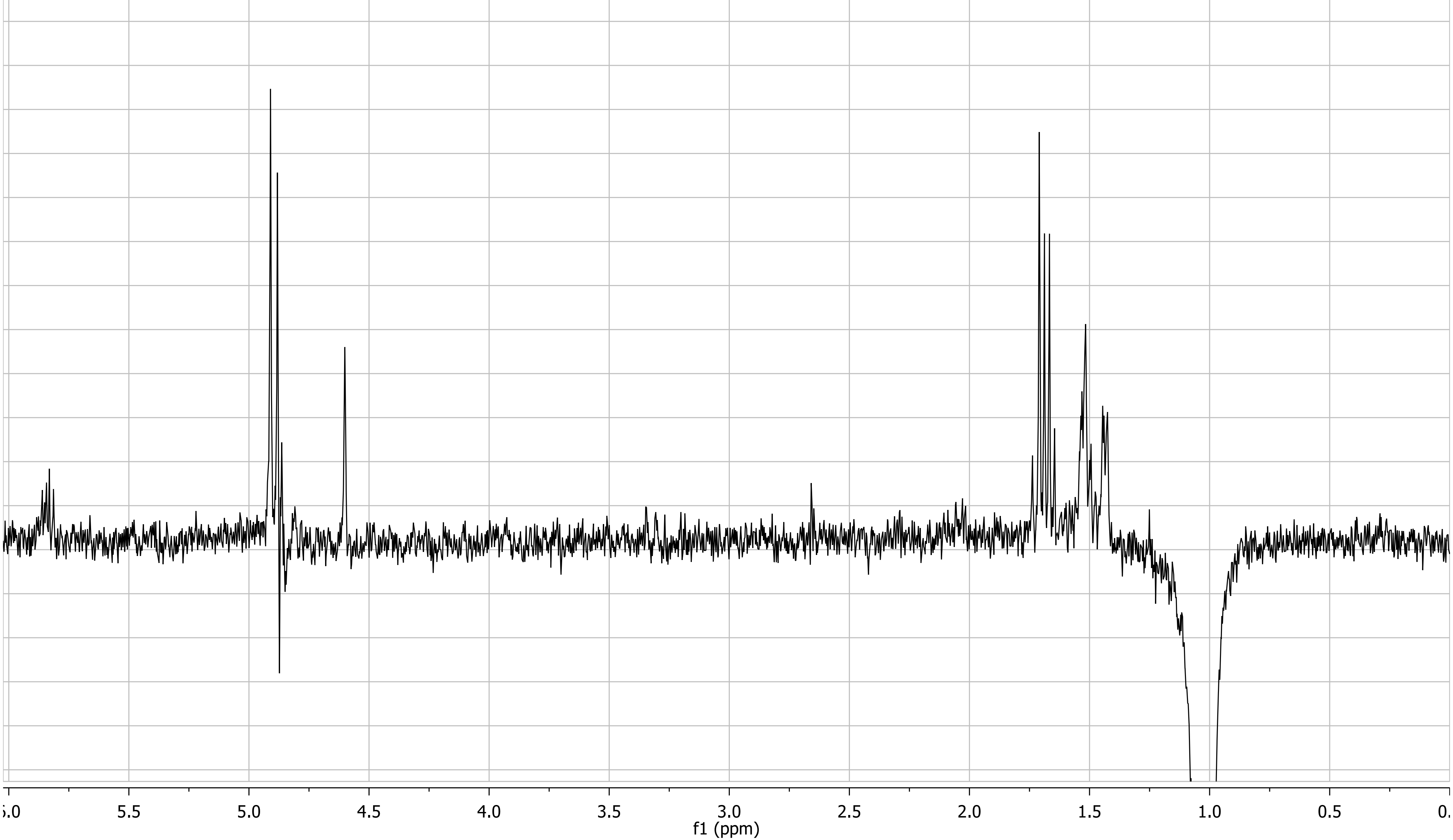

Table S1. ^{1}H and ^{13}C NMR data (300 MHz and 75 MHz, CD_3OD) for loba-8,10,13(15)-triene-16,17,18-triol.

**No.**	**^{13}C δ (m)**	**^{1}H δ (m, J Hz)**	**COSY**	**gHMBC**	**nOe**
1	40.8 (s)				
2	54.0(d)	2.05 (1H, m)	H-3	C-1, C-3, C-7, C-10, C-11, C-12	H-12
3	33.9 (t)	1.68 (1H, m)	H-2, H-4	C-1, C-4	H-7, H-12
		1.50 (1H, m)		C-1, C-4, C-5	
4	48.6 (d)	1.97 (1H, m)	H-3, H_a-5, H_b-5	C-5, C-13, C-15	
5	27.7 (t)	1.60 (1H, m)	H-4, H_b-5, H_b-6	C-1, C-3	
		1.52 (1H, m)	H-4, H_a-5, H_b-6		H-7
6	41.1 (t)	1.53 (1H, m)	H_a-5, H_b-6	C-1, C-7	H-7
		1.43 (1H, m)	H_a-5, H_b-5, H_a-6	C-1, C-2, C-4, C-7	H-7
7	17.1 (q)	1.02 (3H, s)		C-2, C-6, C-8	H_a-3, H_b-5, H_a-6, H_b-6, H-8, H_a-9, H_b-9, H_b-11, H-12, H-14
8	151.6 (d)	5.84 (1H, dd, 10.9, 17.5)	H_a-9, H_b-9	C-1, C-2, C-7, C-8	H-7, H-12
9	110.4 (t)	4.91 (1H, dd, 1.3, 17.5)	H-8, H_b-9	C-1, C-2, C-8	H-7, H-14
		4.87 (1H, dd, 1.3, 10.9)	H-8, H_b-9	C-1, C-2, C-8	H-7, H-12, H-14
10	149.0 (s)				
11	112.7 (t)	4.81 (1H, brt, 1.4)	H_b-11, H_3-12	C-1, C-2, C-10, C-12	H-12
		4.60 (1H, brs)	H_a-11, H_3-12	C-1, C-2, C-10, C-12	H-7, H-12
12	25.3 (q)	1.71 (3H, brs)	H_a-11, H_b-11	C-1, C-2, C-10, C-11	H-2, H_a-3, H-7, H-8, H_b-9, H_a-11, H_b-11, H-15, H-20
13	151.6 (s)				
14	15.3 (q)	1.74 (3H, d, 1.1)		C-4, C-13, C-15	H-7, H_a-9, H_b-9, H-15, H-16, H-17, H-20
15	125.6 (d)	5.49 (1H, brd, 8.8)	H-14, H-16	C-3, C-4, C-14, C-17	H-12, H-14, H-16, H-17
16	68.5 (d)	4.65 (1H, dd, 3.0, 8.8)	H-15, H-17	C-13,C-15, C-18	H-14, H-15, H-17, H-18, H-19, H-20
17	80.3 (d)	3.15 (1H, d, 3.0)	H-16	C-15, C-18, C-19, C-20	H-14, H-15, H-16, H-19, H-20
18	74.2 (s)				H-16
19	26.5 (q)	1.23 (3H, s)		C-17, C-18, C-20	H-16, H-17
20	27.0 (q)	1.25 (3H, s)		C-17, C-18, C-19	H-12, H-14, H-16, H-17

S36. ^{1}H NMR of lobatrienolide in CD_3OD

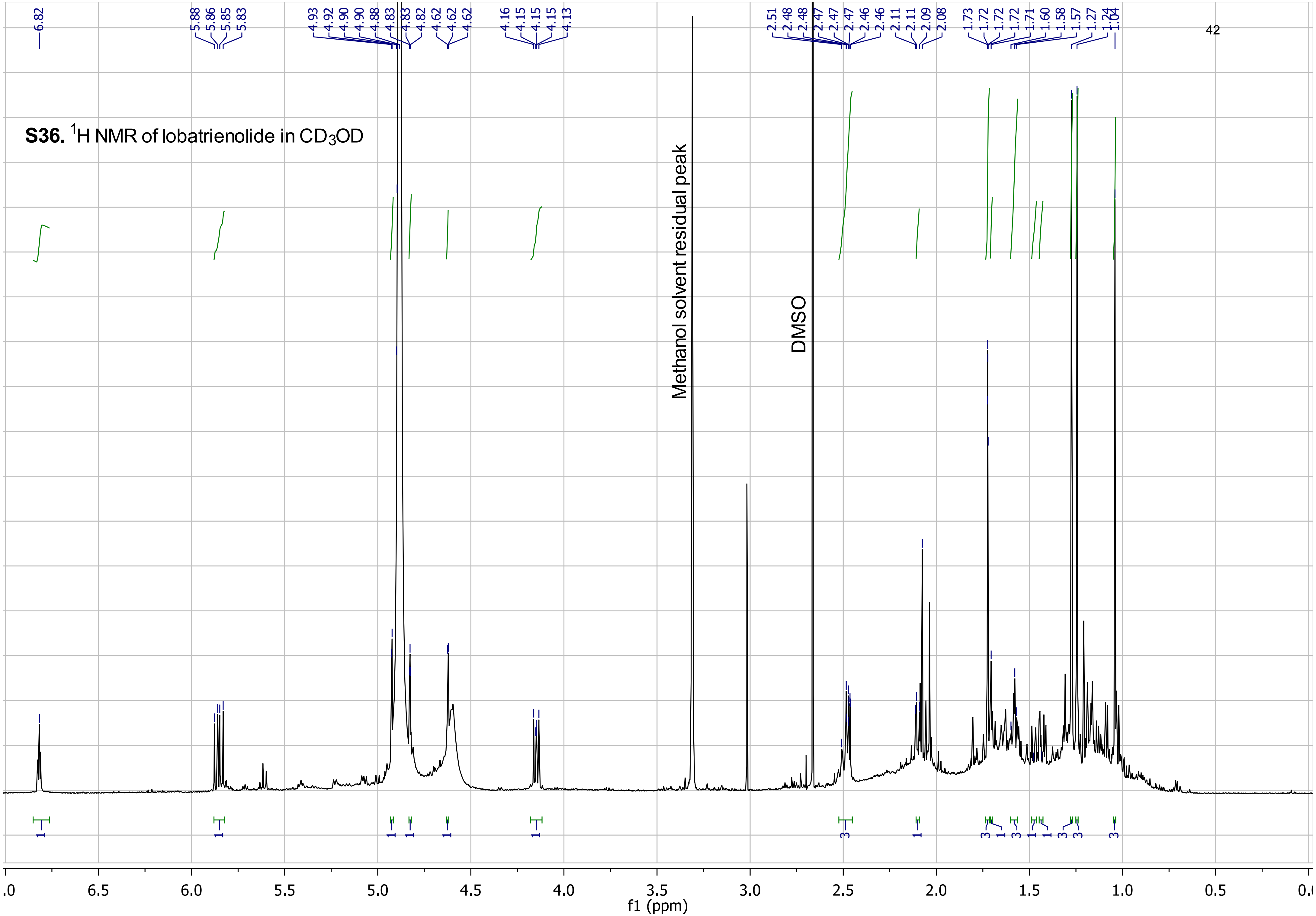

S37. ^{13}C NMR of lobatrienolide in CD_3OD

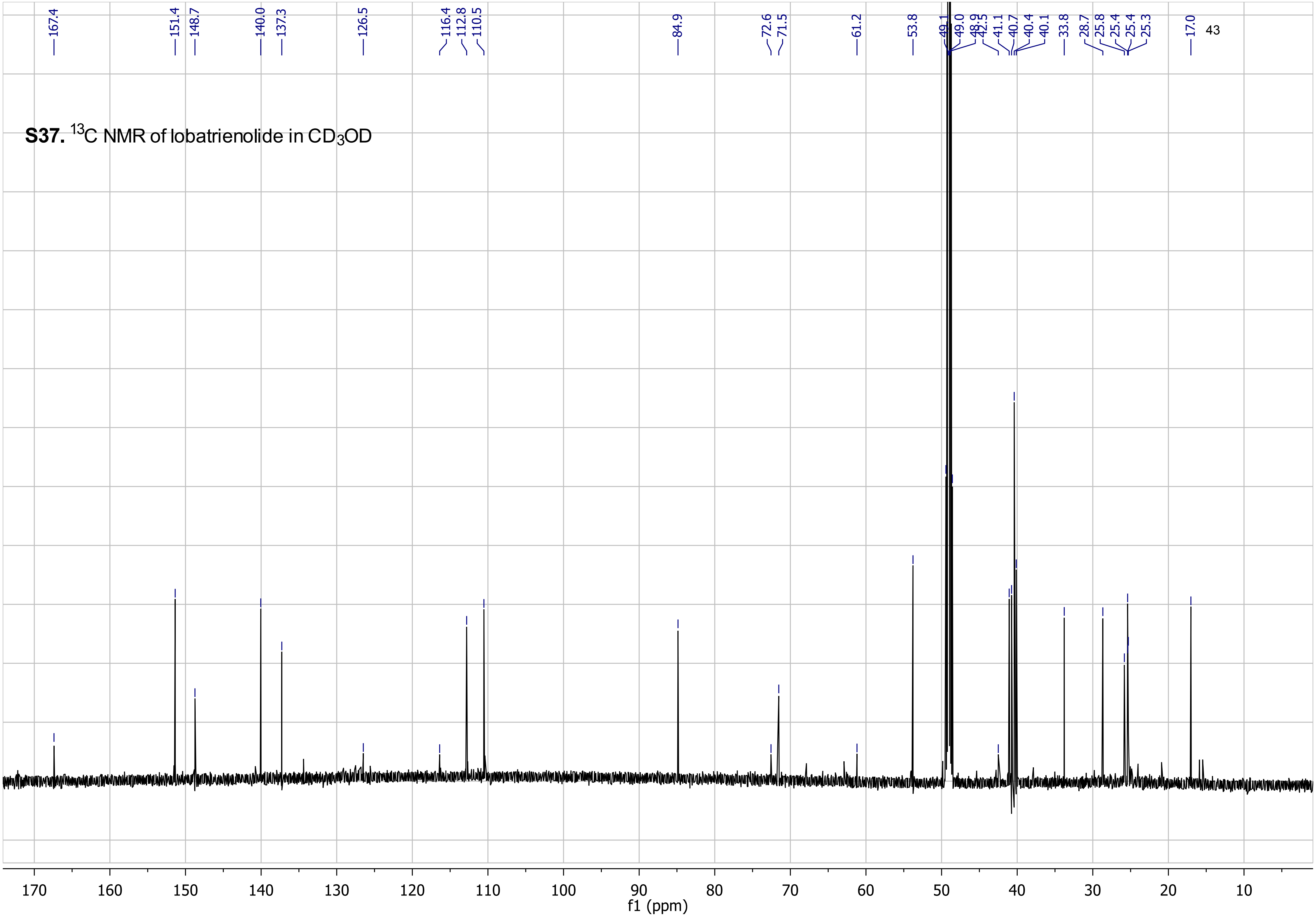

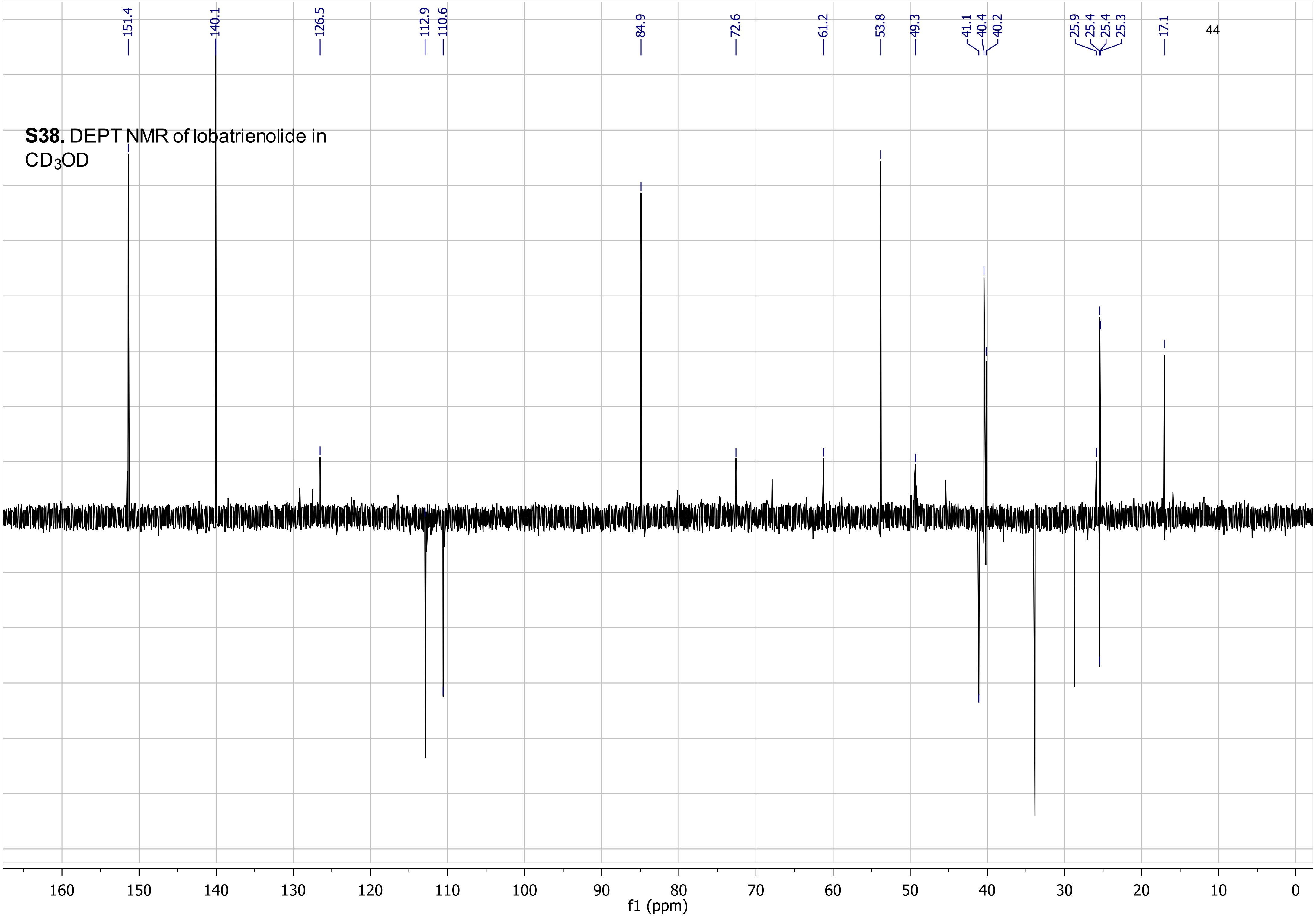

S38. DEPT NMR of lobatrienolide in CD_3OD

S39. COSY NMR of lobatrienolide in CD_3OD

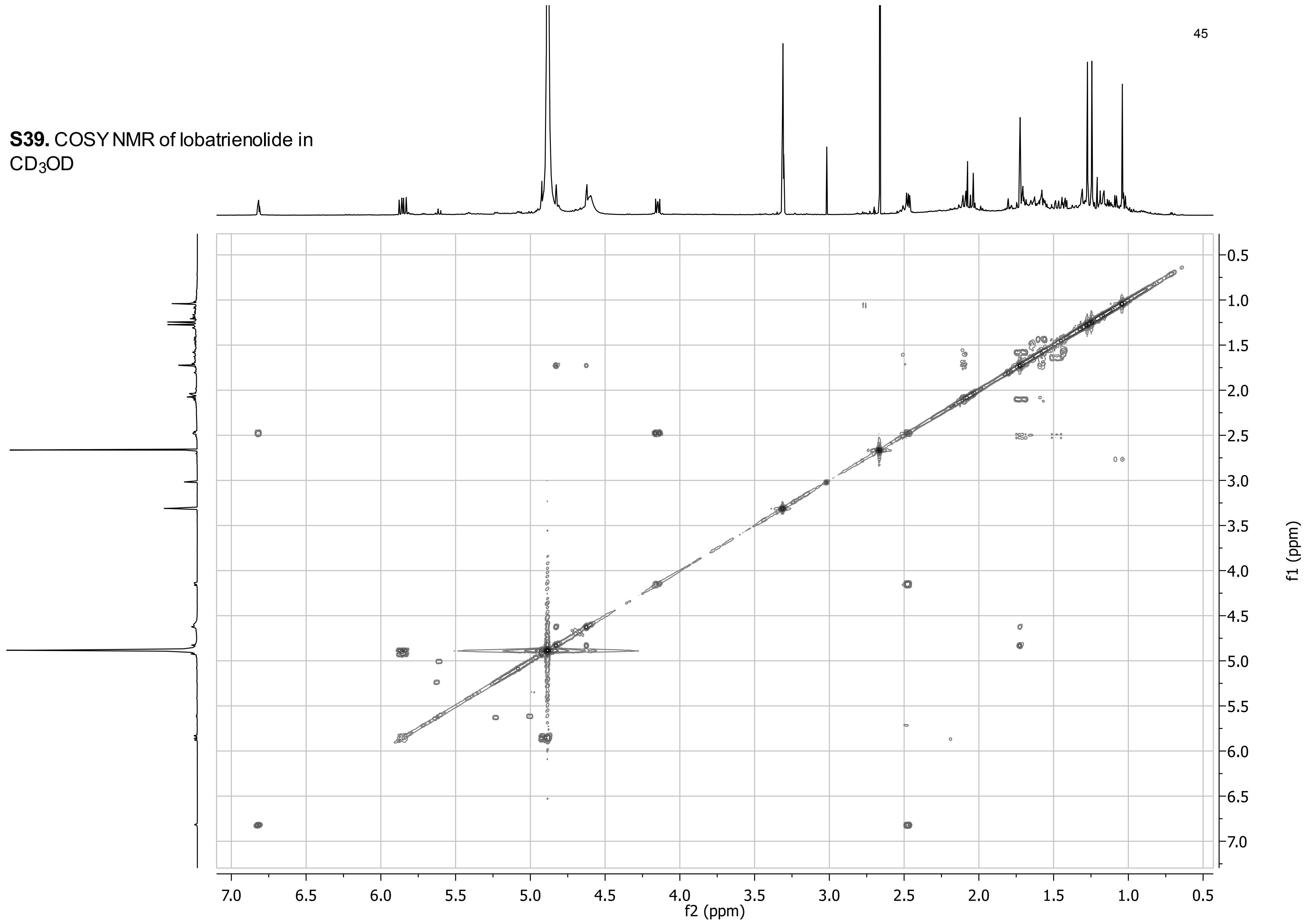

S40. HSQC NMR of lobatrienolide in CD_3OD

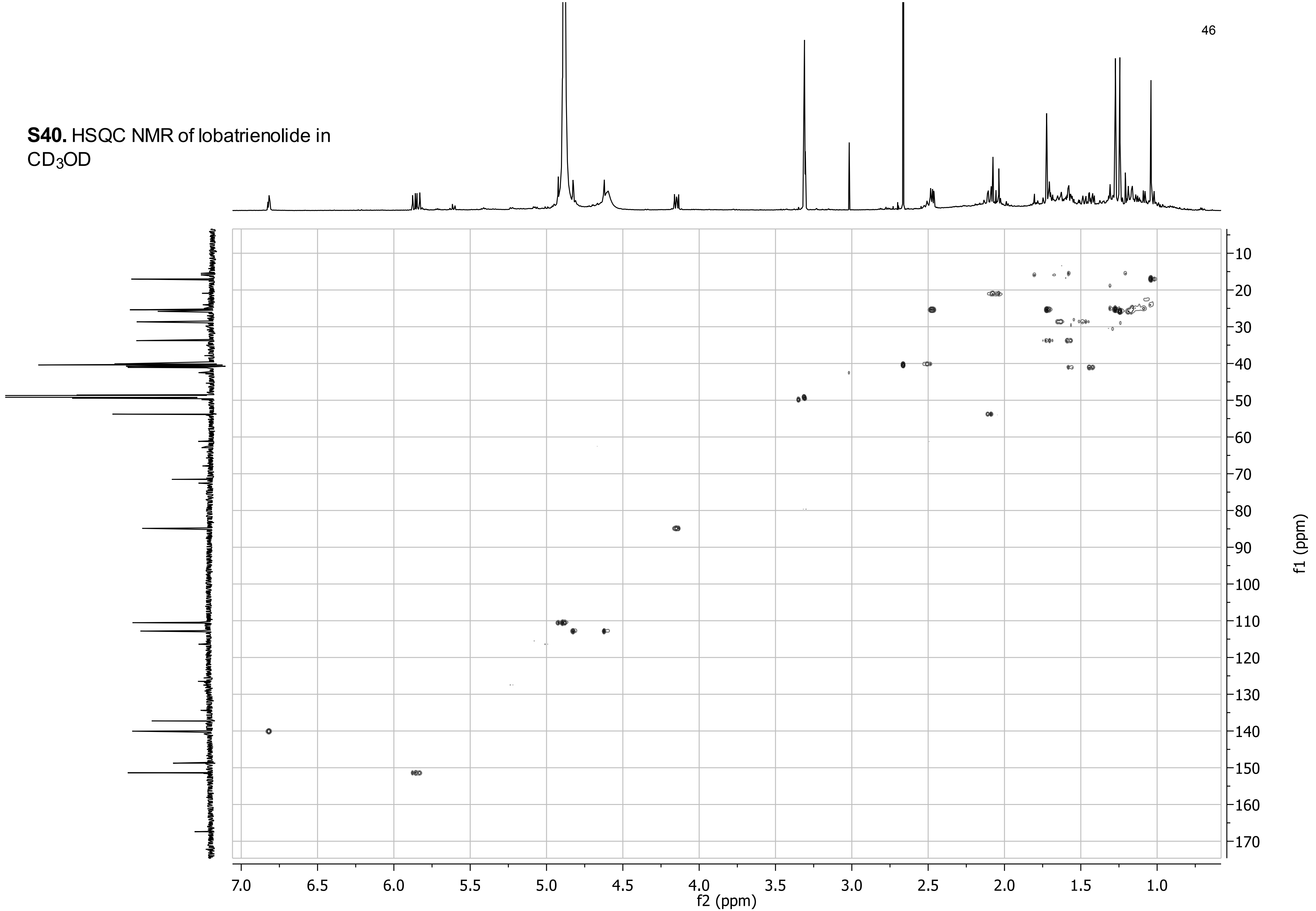

S41. HMBC NMR of lobatrienolide in CD_3OD

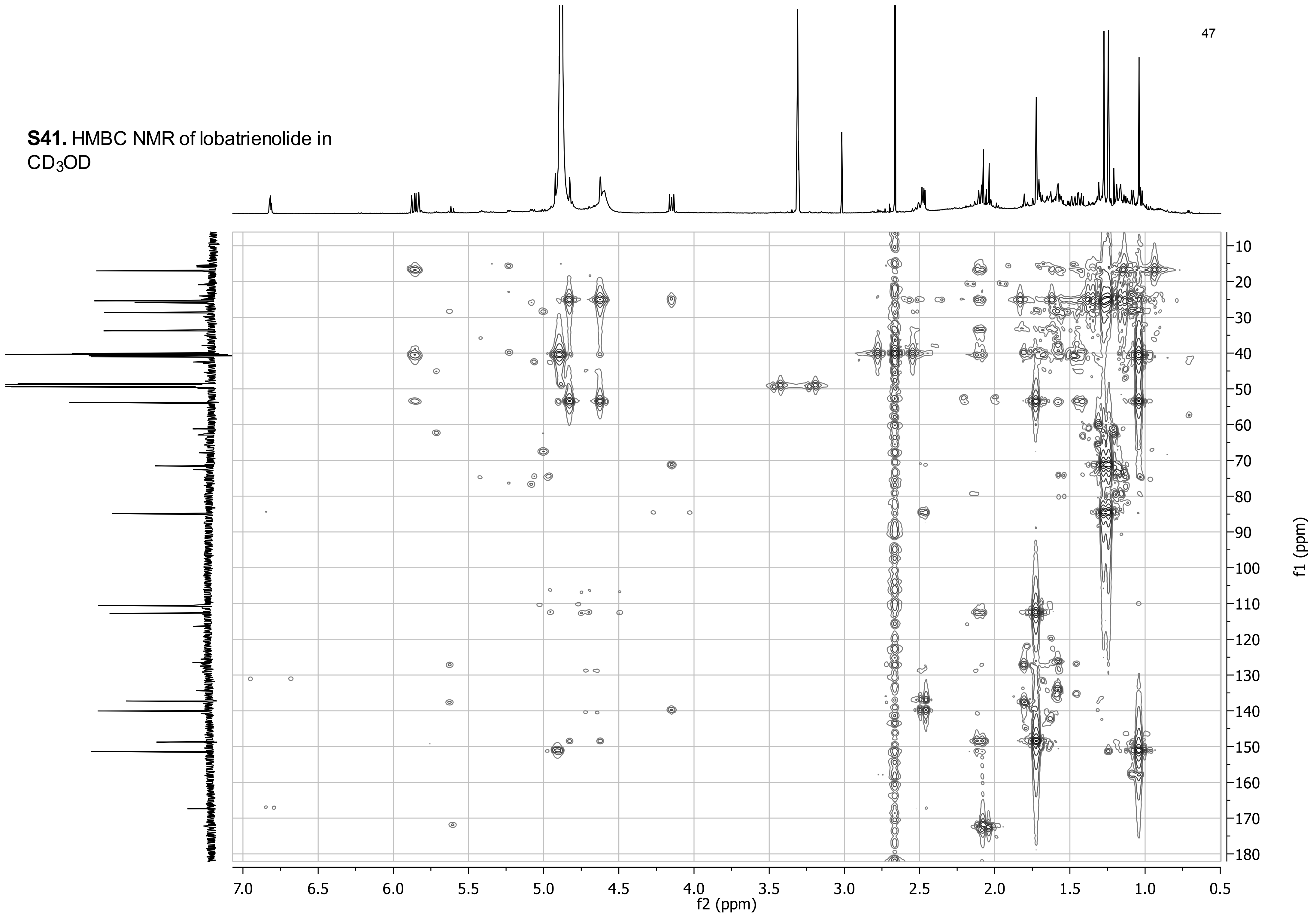

Table S2. ^{1}H and ^{13}C NMR data (300 MHz and 75 MHz, CD_3OD) for lobatrienolide.

**No.**	**^{13}C δ (m)**	**^{1}H δ (m, J Hz)**	**COSY**	**gHMBC**
1	40.7 (s)			
2	53.8(d)	2.10 (1H, dd, 3.5, 12.7)	H_a-3, H_b-3	C-1, C-3, C-7, C-10, C-11, C-12
3	33.8 (t)	1.71 (1H, m)	H-2, H_b-3, H4	C-1, C-2, C-10, C-11
		1.58 (1H, m)	H-2, H_a-3, H-4	C-1, C-2, C-4, C-5
4	40.2 (d)	2.51 (1H, m)	H_a-3, H_b-3, H_a-5, H_b-5	C-13, C-14, C-15
5	28.5 (t)	1.60 (1H, m)	H4	C-3, C-7
		1.48 (1H, m)	H-4	C-1, C-4
6	41.1 (t)	1.57 (1H, m)	H_b-6	C-1, C-2, C-4, C-5, C-7
		1.43 (1H, m)	H_a-6	C-1, C-2, C-4, C-5, C-7
7	17.1 (q)	1.04 (3H, s)		C-1, C-2, C-5, C-8
8	151.4 (d)	5.85 (1H, dd, 10.8, 17.5)	H-9	C-1, C-2, C-7
9	110.5 (t)	4.92 (1H, dd, 1.4, 17.5)	H-8	C-1, C-2, C-8
		4.89 (1H, dd, 1.4, 10.8)	H-8	C-1, C-2, C-8
10	148.8 (s)			
11	112.8 (t)	4.83 (1H, dq, 1.5, 3.2)	H_b-11, H_3-12	C-1, C-2, C-10, C-12
		4.62 (1H, m)	H_a-11, H_3-12	C-1, C-2, C-10, C-12
12	25.3 (q)	1.72 (3H, brdd, 0.8, 1.5)	H_a-11, H_b-11	C-1, C-2, C-10, C-11
13	137.3 (s)			
14	167.4 (s)			
15	140.1 (d)	6.82 (1H, ddd, 0.9, 3.6, 4.5)	H-16	C-4, C-14, C-16, C-17
16	25.5 (t)	2.48 (2H, m)	H-15, H-17	C-4, C-13, C-15, C-17, C-18, C-19, C-20
17	84.9 (d)	4.15 (1H, m)	H-16	C-14, C-15, C-18, C-19, C-20
18	71.5 (s)			
19	25.9 (q)	1.24 (3H, s)		C-17, C-18, C20
20	25.4 (q)	1.27 (3H, s)		C-17, C-18, C-19

S42. ^1H NMR of lobatrientriol in CD_3OD

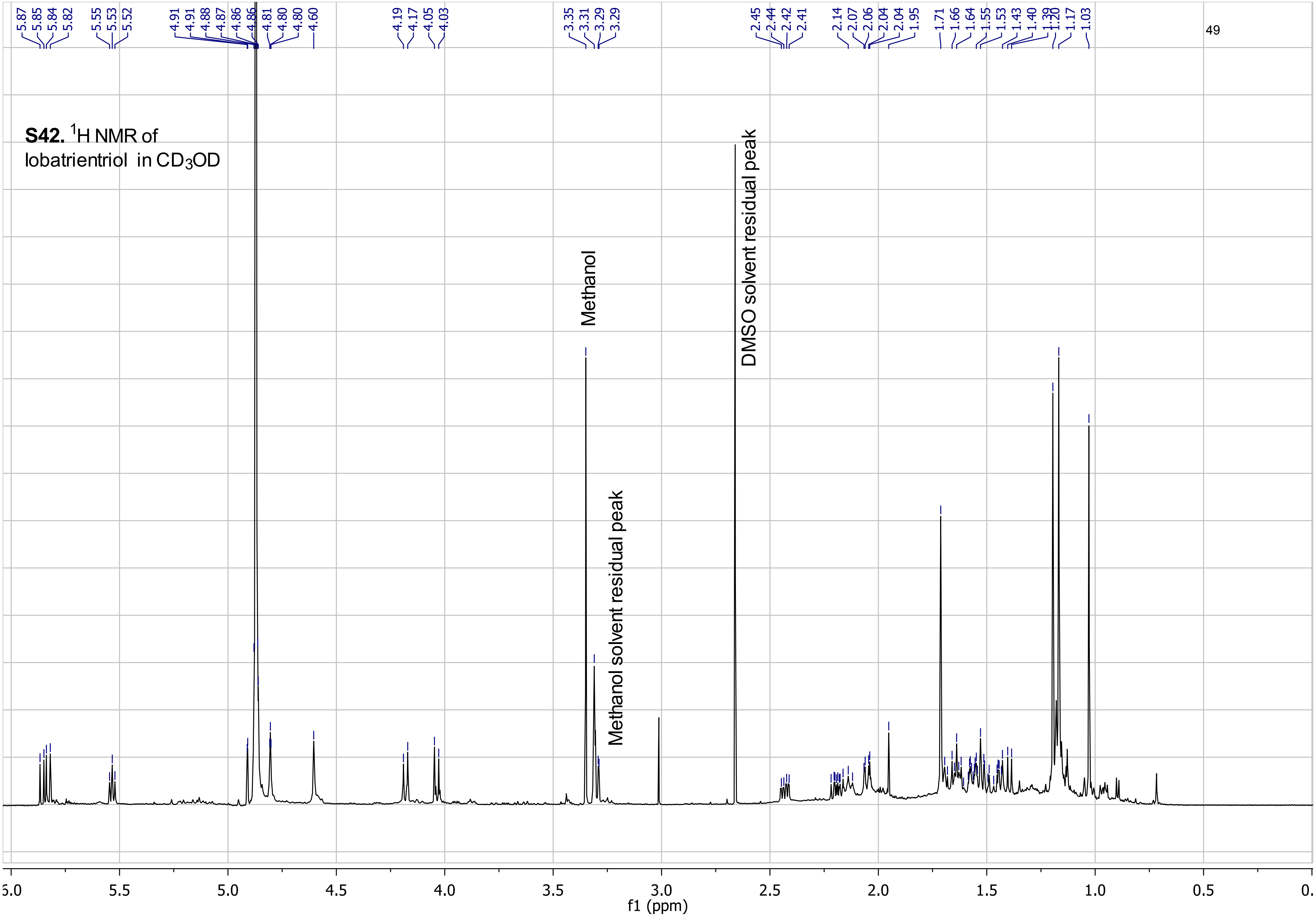

S43. ^{13}C NMR of lobatrientriol in CD_3OD

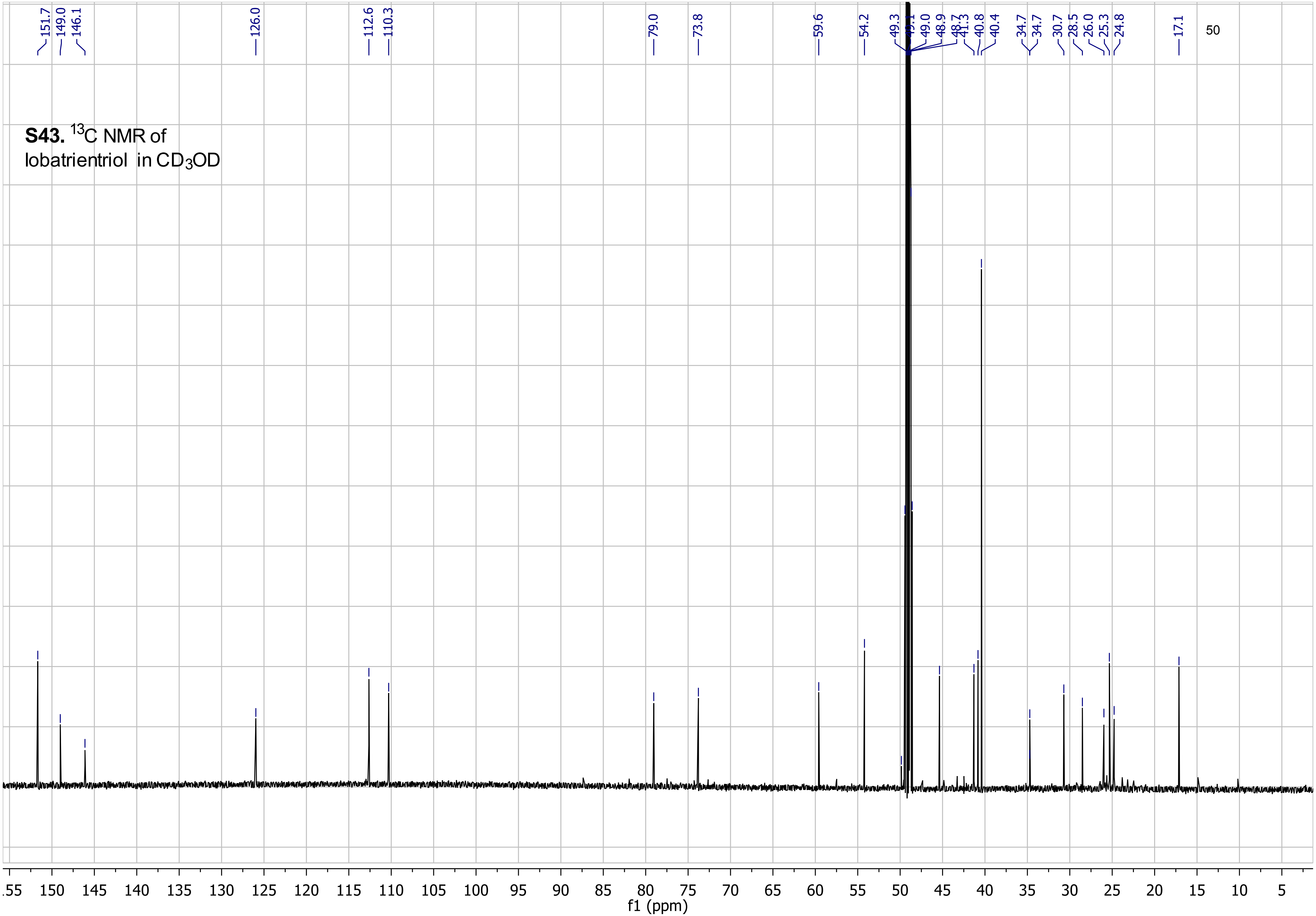

S44. COSY NMR of lobatrientriol in CD_3OD

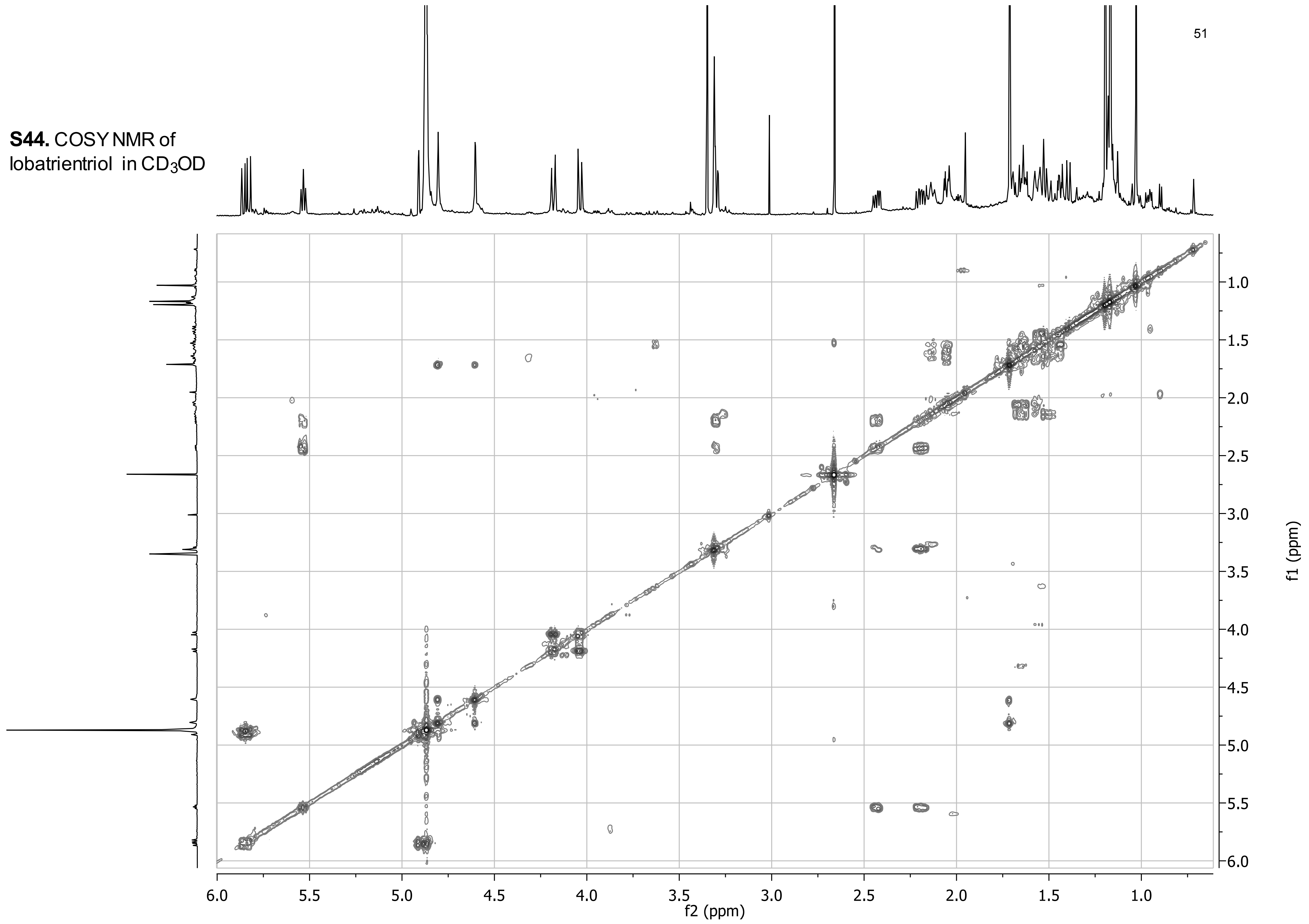

S45. HSQC NMR of lobatrientriol in CD_3OD

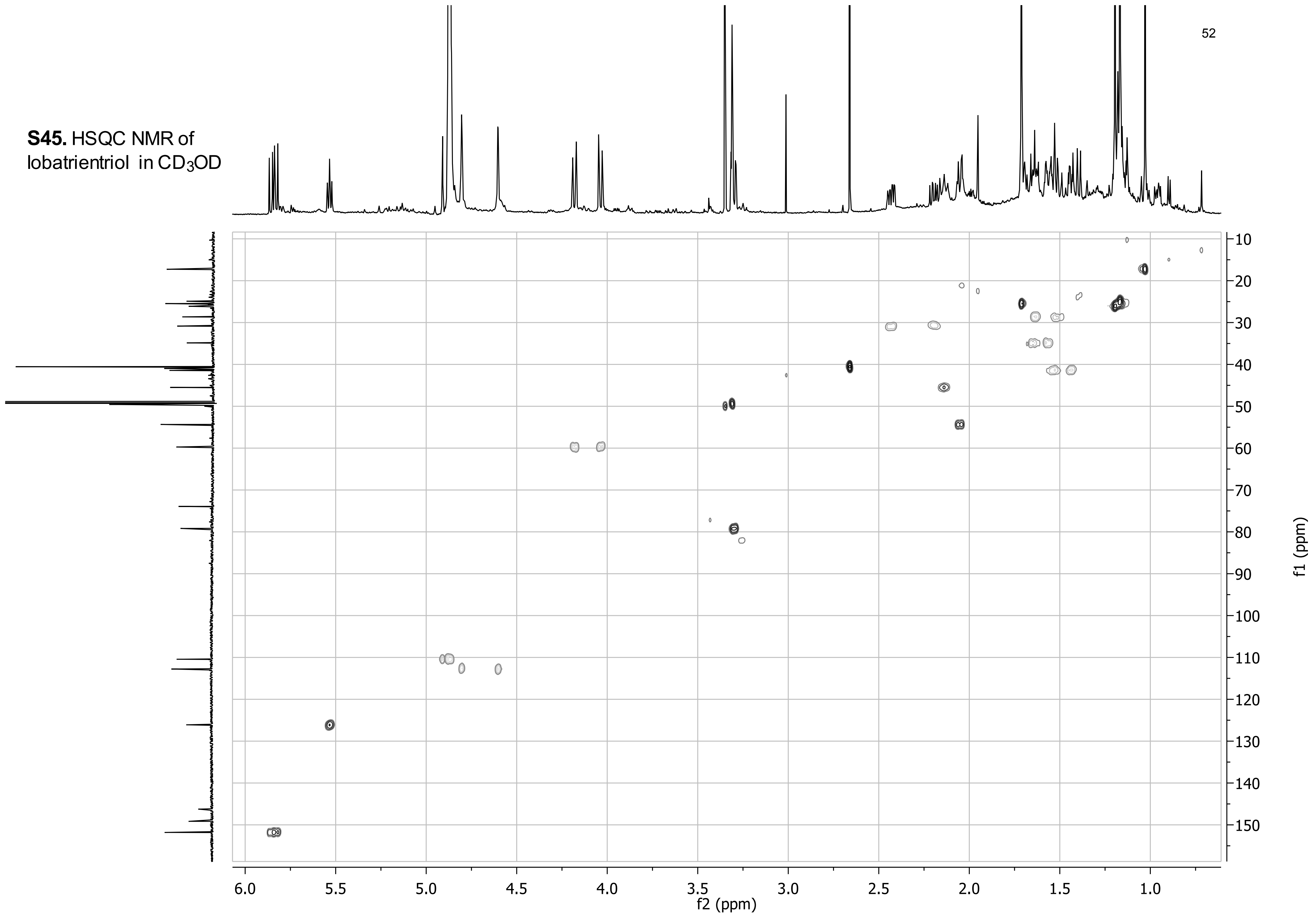

S46. HMBC NMR of lobatrientriol in CD_3OD

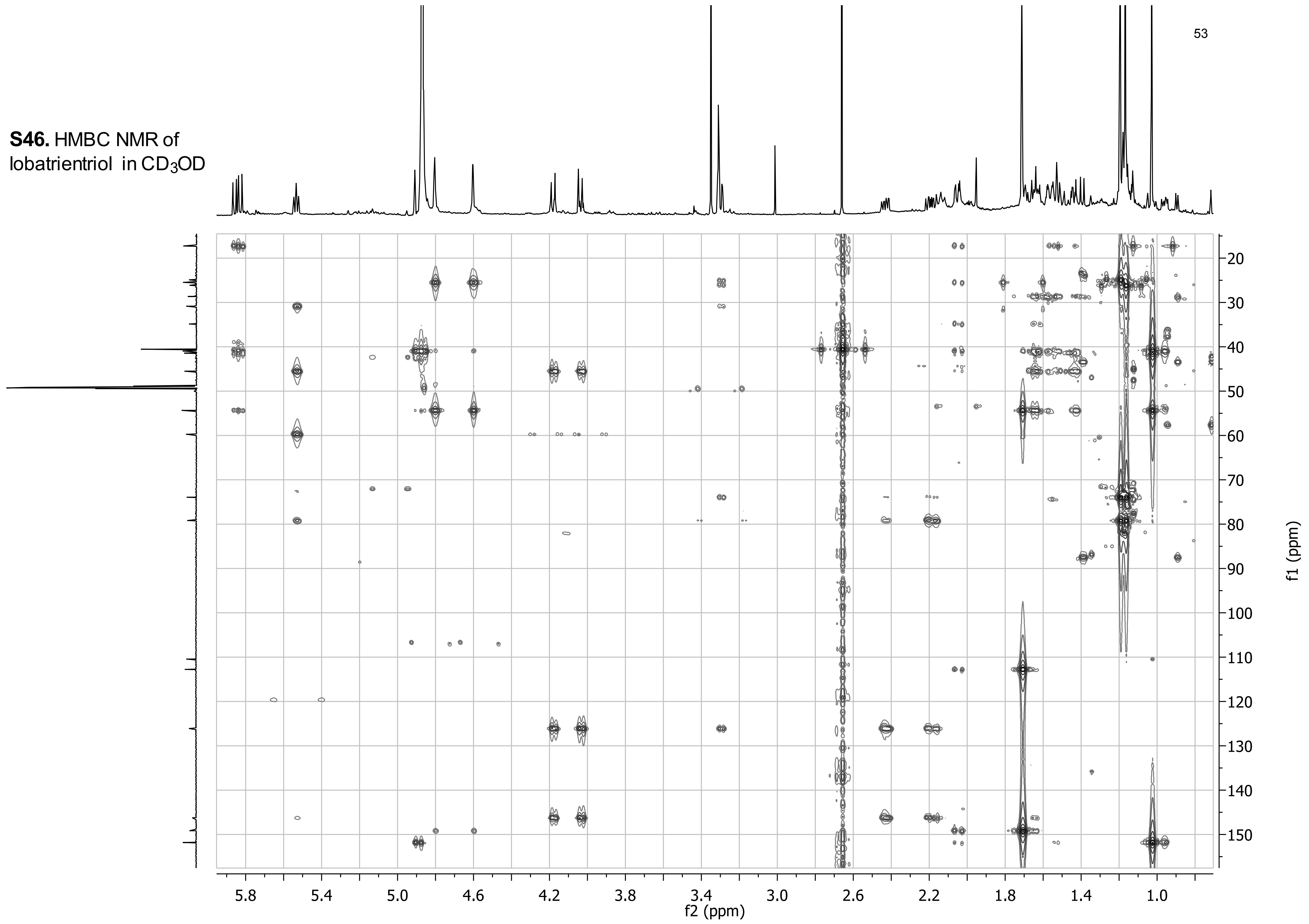

Table S3. ^{1}H and ^{13}C NMR data (300 MHz and 75 MHz, CD_3OD) for lobatrientriol.

No.	^{13}C δ (m)	^{1}H δ (m, J Hz)	COSY	gHMBC
1	40.8 (s)			
2	54.2(d)	2.05 (1H, dd, 3.1, 12.8)	H_a-3, H_b-3	C-1, C-3, C-4, C-7, C-8, C-10, C-11, C-12
3	34.7 (t)	1.65 (1H, m)	H-2, H-4	C-1, C-2, C-4, C-5
		1.57 (1H, m)	H-2, H-4	C-1, C-2, C-4, C-5
4	45.4 (d)	2.14 (1H, m)	H_a-3, H_b-3, H_a-5, H_b-5	C-2, C-3, C-5, C-13, C-14, C-15
5	28.5 (t)	1.64 (1H, m)	H-4, H_b-5, H_b-6	C-1, C-2, C-4
		1.52 (1H, m)	H-4, H_a-5, H_b-6	C-1, C-4
6	41.3 (t)	1.53 (1H, m)	H_a-5, H_b-6	C-1, C-4, C-5, C-7
		1.45 (1H, m)	H_a-5, H_b-5, H_a-6	C-1, C-2, C-4, C-7
7	17.1 (q)	1.03 (3H, s)		C-1, C-2, C-6, C-8, C-9
8	151.7 (d)	5.84 (1H, dd, 10.8, 17.6)	H_a-9, H_b-9	C-1, C-2, C-6, C-7
9	110.3 (t)	4.91 (1H, d, 1.4, 17.6)	H-8, H_b-9	C-1, C-2, C-8
		4.87 (1H, 1.4, 10.8)	H-8, H_b-9	C-1, C-2, C-8
10	149.0 (s)			
11	112.6 (t)	4.81 (1H, dq, 1.4, 3.2)	H_b-11, H_3-12	C-1, C-2, C-10, C-12
		4.60 (1H, brs)	H_a-11, H_3-12	C-1, C-2, C-10, C-12
12	25.3 (q)	1.71 (3H, brs)	H_a-11, H_b-11	C-1, C-2, C-10, C-11
13	146.1 (s)			
14	59.6 (t)	4.18 (1H, d, 11.8)	H_b-14, H-15	C-4, C-13, C-15
		4.04 (1H, d, 11.8)	H_a-14	C-4, C-13, C-15
15	126.0(d)	5.53 (1H, brdd, 7.3, 8.2)	H_a-14, H_a-16, H_b-16	C-4, C-13, C-14, C-16, C-17
16	30.7(t)	2.43 (1H, ddd, 2.3, 7.3, 14.6)	H-15, H_b-16, H-17	C-13,C-15, C-17, C-18
		2.19 (1H, ddd, 8.2, 10.3, 14.6)	H-15, H_a-16, H-17	C-13,C-15, C-17, C-18
17	79.1 (d)	3.29 (1H, brd, 2.3)	H_a-16, H_b-16	C-15, C-16, C-18, C-19, C-20
18	73.8 (s)			
19	24.8 (q)	1.17 (3H, s)		C-17, C-18, C-20
20	26.0(q)	1.20 (3H, s)		C-17, C-18, C-19

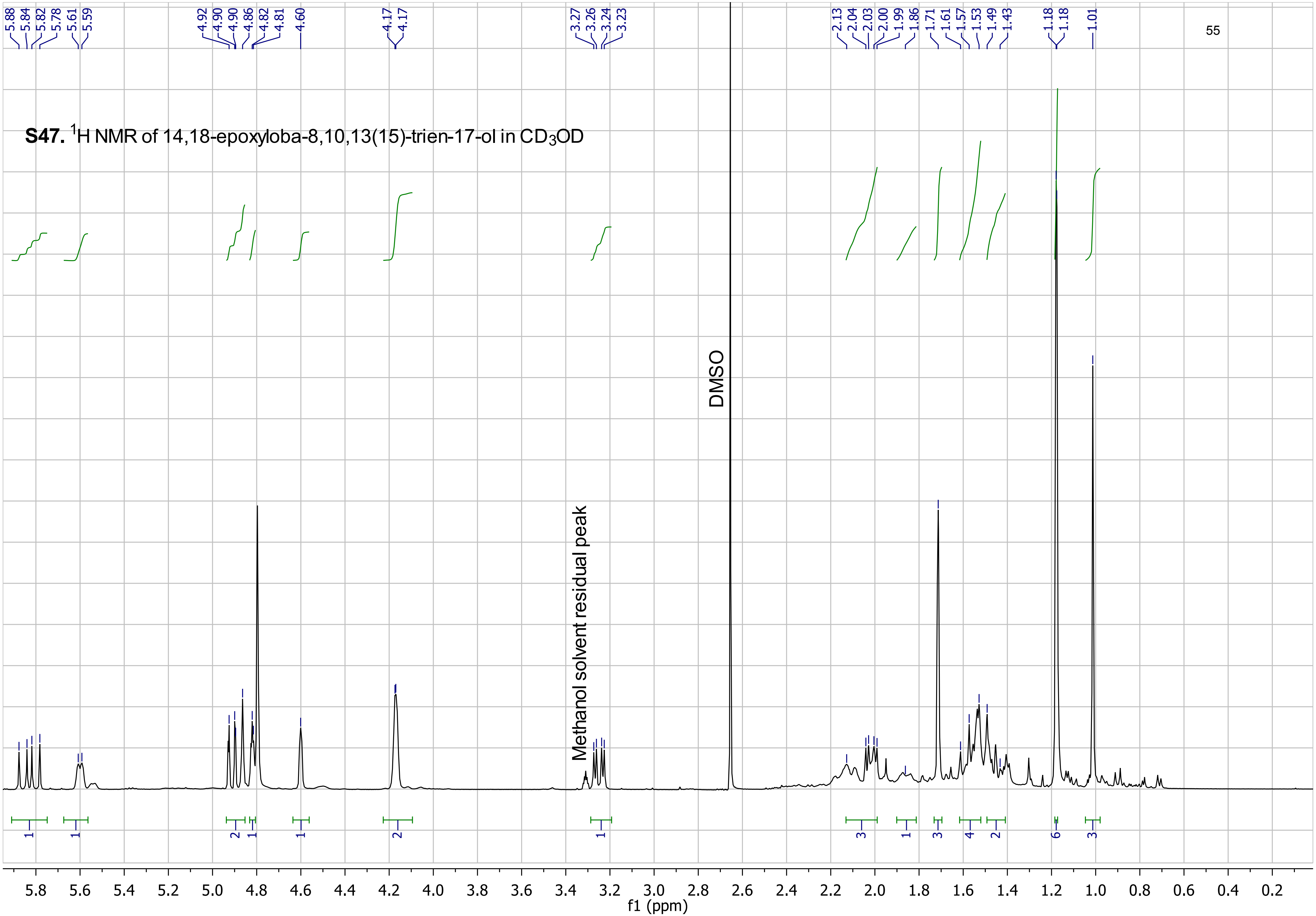
S47. 1H NMR of 14,18-epoxyloba-8,10,13(15)-trien-17-ol in CD3OD
5.88 5.84 5.82 5.78 5.61 5.59
4.92 4.90 4.90 4.86 4.82 4.81 4.60
4.17 4.17
3.27 3.26 3.24 3.23
2.13 2.04 2.03 2.00 1.99 1.86 1.71 1.61 1.57 1.53 1.49 1.43
1.18 1.18 1.01
DMSO
Methanol solvent residual peak
1 1 2 1 1 2 1 3 1 3 4 2 6 3
5.8 5.6 5.4 5.2 5.0 4.8 4.6 4.4 4.2 4.0 3.8 3.6 3.4 3.2 3.0 2.8 2.6 2.4 2.2 2.0 1.8 1.6 1.4 1.2 1.0 0.8 0.6 0.4 0.2
f1 (ppm)

S48. ^{13}C NMR of 14,18-epoxyloba-8,10,13(15)-trien-17-ol in CD_3OD

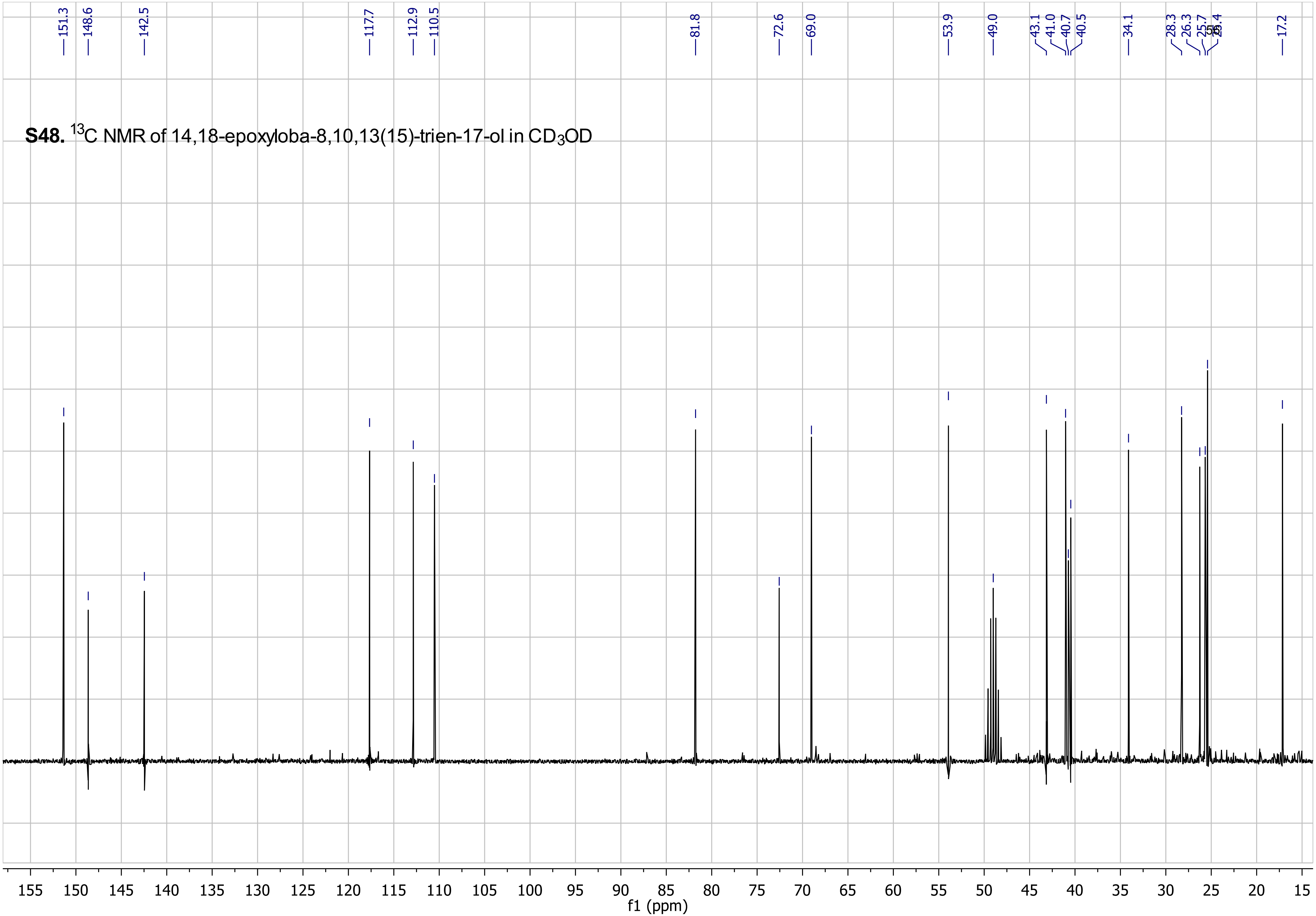

S49. DEPT NMR of 14,18-epoxyloba-8,10,13(15)-trien-17-ol in CD_3OD

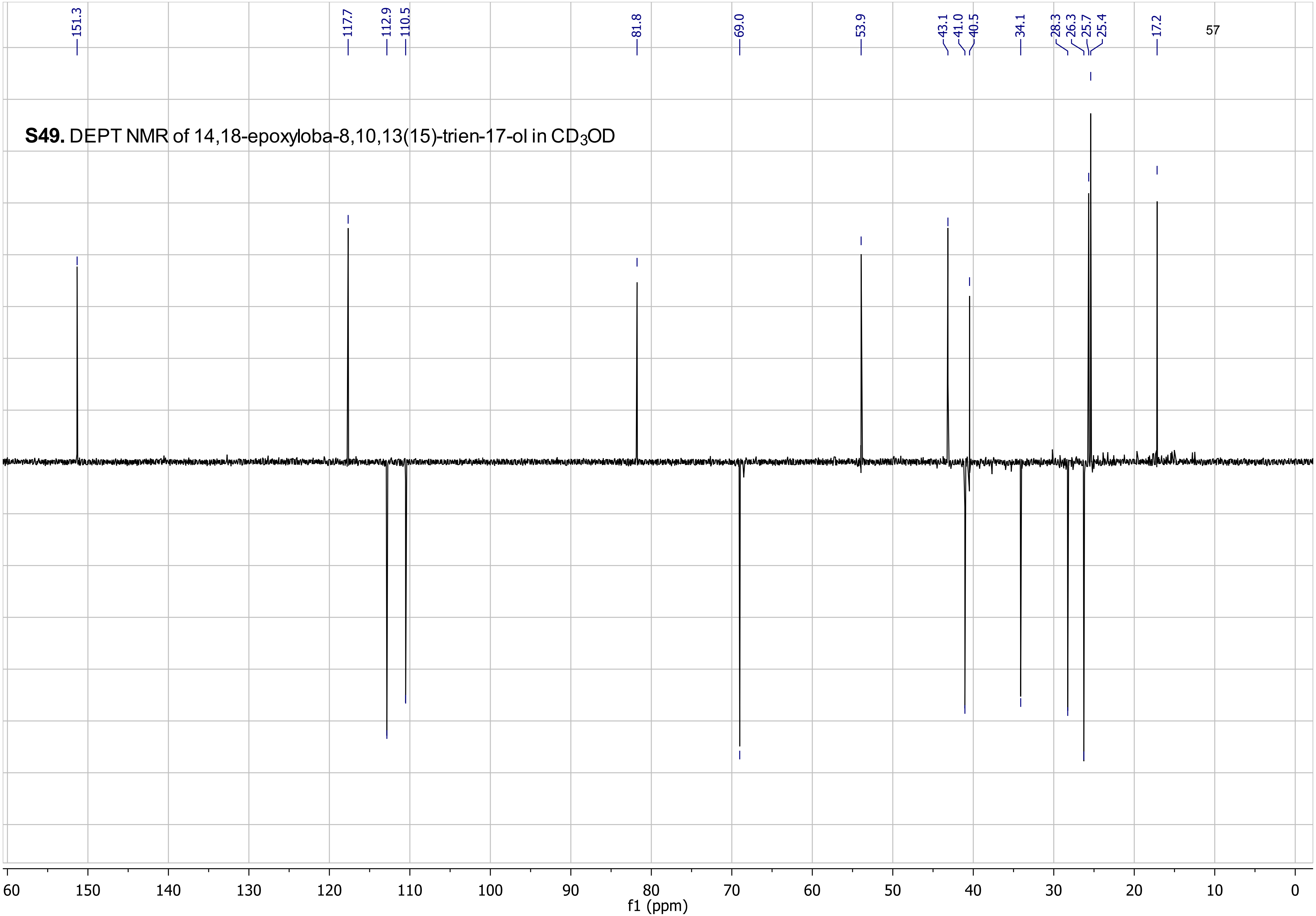

S50. COSY NMR of 14,18-epoxyloba-8,10,13(15)-trien-17-ol in CD_3OD

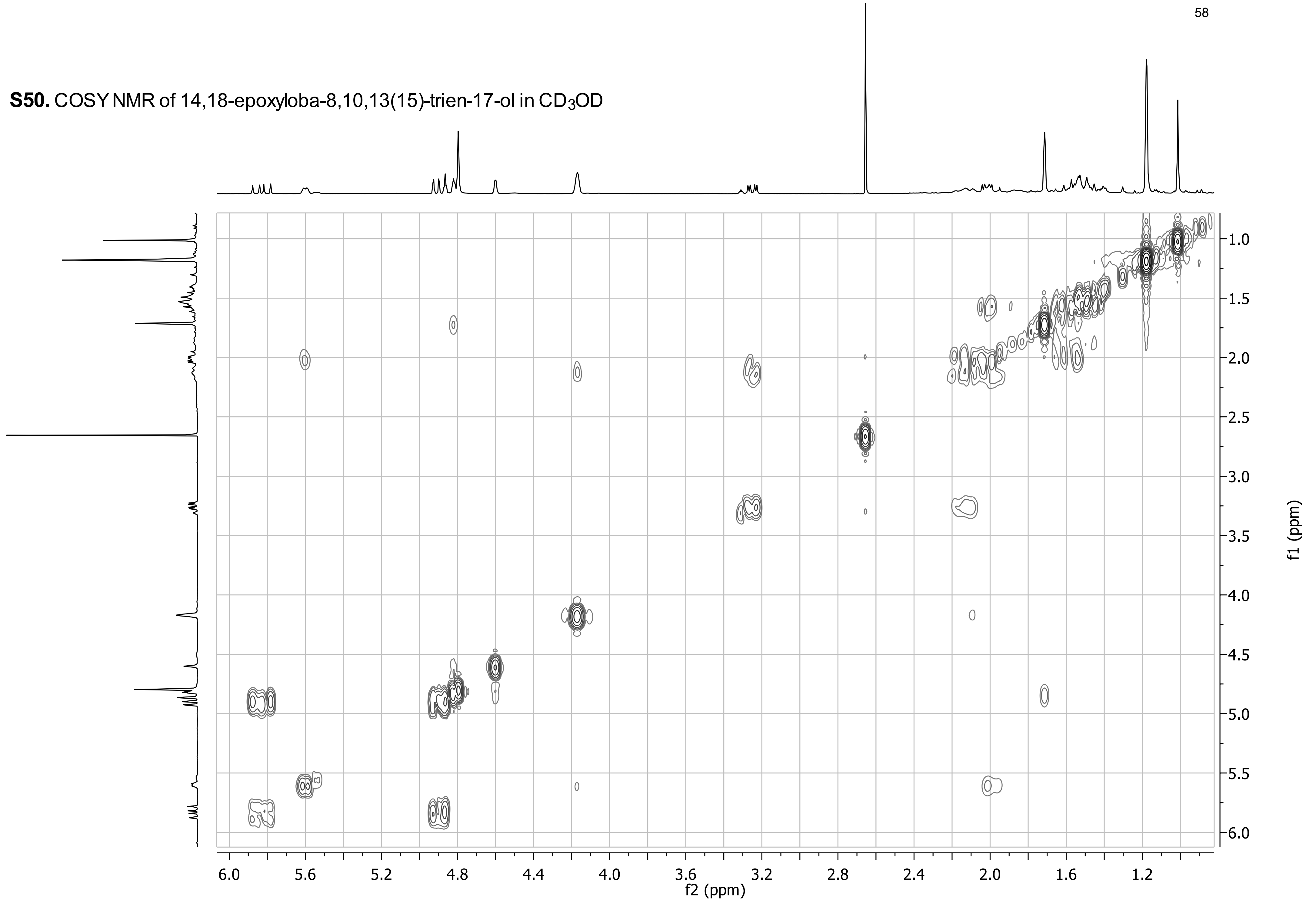

S51. HSQC NMR of 14,18-epoxyloba-8,10,13(15)-trien-17-ol in CD_3OD

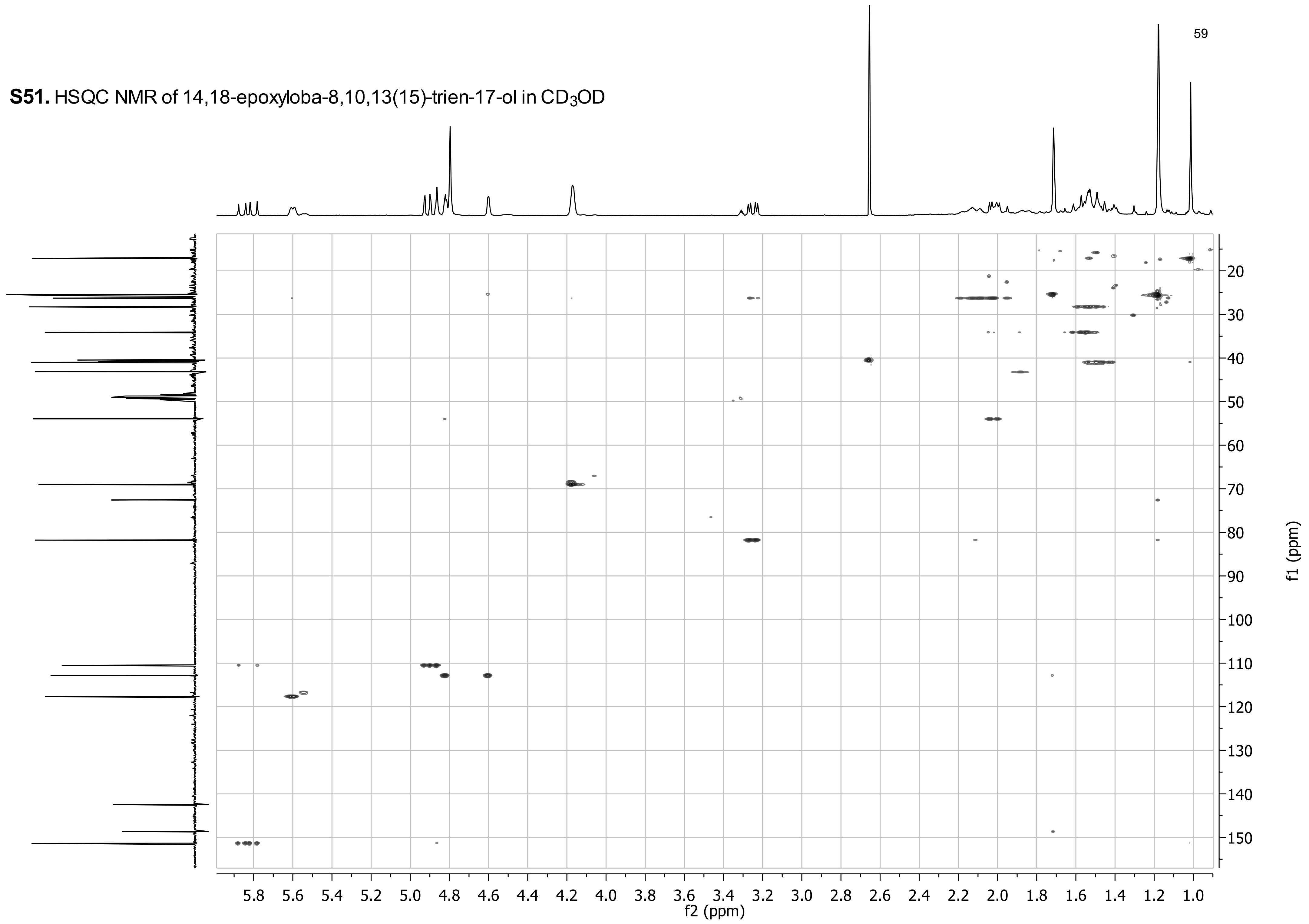

S52. HMBC NMR of 14,18-epoxyloba-8,10,13(15)-trien-17-ol in CD_3OD

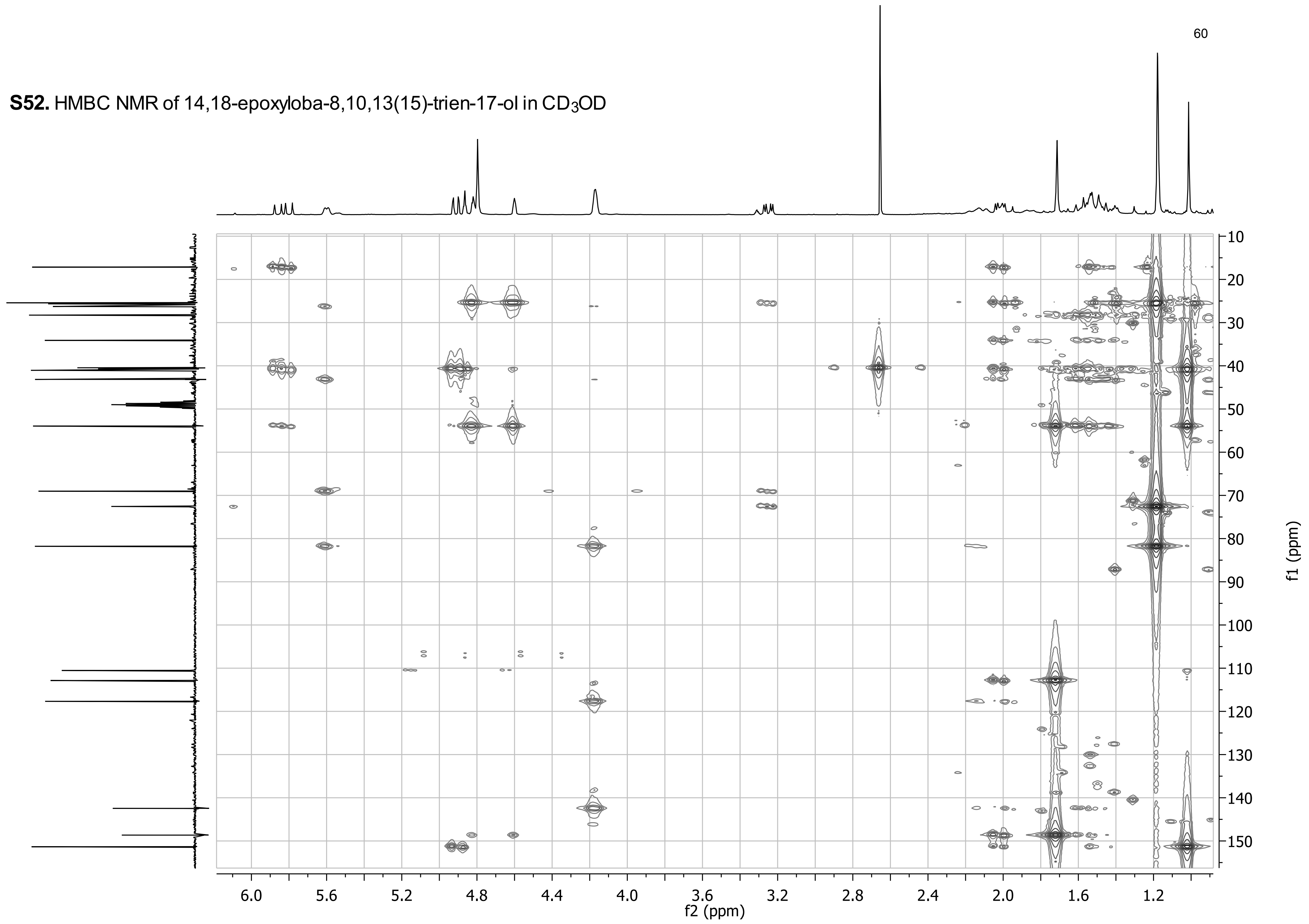

Table S4. ^{1}H and ^{13}C NMR data (300 MHz and 57 MHz, CD_3OD) for 14,18-epoxyloba-8,10,13(15)-trien-17-ol.

No.	^{13}C δ (m)	^{1}H δ (m, J Hz)	COSY	gHMBC
1	40.7 (s)			
2	53.9(d)	2.01 (1H, dd, 4.0, 11.2)	H_a-3, H_b-3	C-1, C-6, C-7, C-8, C-10, C-11, C-12
3	34.1 (t)	1.60 (1H, m)	H-2, H_b-3, H4	C-2, C-4, C-5, C-10, C-13
		1.52 (1H, m)	H-2, H_a-3, H-4	C-1, C-2, C-4, C-5, C-10, C-12, C-13
4	43.2 (d)	1.86 (1H, m)	H_b-3, H_a-5, H_b-5	C-3, C-5, C-13
5	28.3 (t)	1.57 (1H, m)	H4	C-3, C-2, C-4, C-13
		1.48 (1H, m)	H-4	C-3, C-4
6	41.0 (t)	1.52 (1H, m)	H_b-6	C-2, C-3, C-5, C-7, C-8, C-10
		1.43 (1H, m)	H_a-6	C-2, C-3, C-5, C-7, C-8, C-10
7	17.2 (q)	1.01 (3H, s)		C-1, C-2, C-6, C-8
8	151.3 (d)	5.83 (1H, 10.9, 17.5)	H-9	C-1, C-2, C-6, C-7
9	110.5 (t)	4.90 (1H, dd, 1.4, 17.5)	H-8, H_b-9	C-1, C-8
		4.88 (1H, t, 1.4, 10.9)	H-8, H_b-9	C-1, C-8
10	148.6 (s)			
11	112.9 (t)	4.82 (1H, dq, 1.3, 2.9)	H_b-11, H_3-12	C-1, C-2, C-10, C-12
		4.60 (1H, brs)	H_a-11, H_3-12	C-1, C-2, C-10, C-12
12	25.4 (q)	1.71 (3H, brs)	H_a-11, H_b-11	C-1, C-2, C-10, C-11
13	142.5 (s)			
14	69.0 (t)	4.17 (2H, m)	H-15	C-4, C-13, C-15, C-16, C-17
15	117.7(d)	5.60 (1H, brddd, 1.2, 3.7, 3.8)	H-14	C-4, C-14, C-16, C-17
16	26.3 (t)	2.13 (1H, m)	H-15, H_b-16, H-17, H-14	C-13, C-15, C-17
		2.01 (1H, m)	H-15, H_a-16, H-17, H-14	
17	82.1 (d)	3.25 (1H, dd, 3.6, 10.7)	H-16	C-14, C-16, C-18
18	72.6 (s)			
19	25.7 (q)	1.17 (3H, s)		C-16, C-17, C-18
20	25.7 (q)	1.18 (3H, s)		C-16, C-17, C-18

S53. ^{1}H NMR of 14,17-epoxyloba-8,10,13(15)-trien-18-ol-18-acetate in CD_3OD

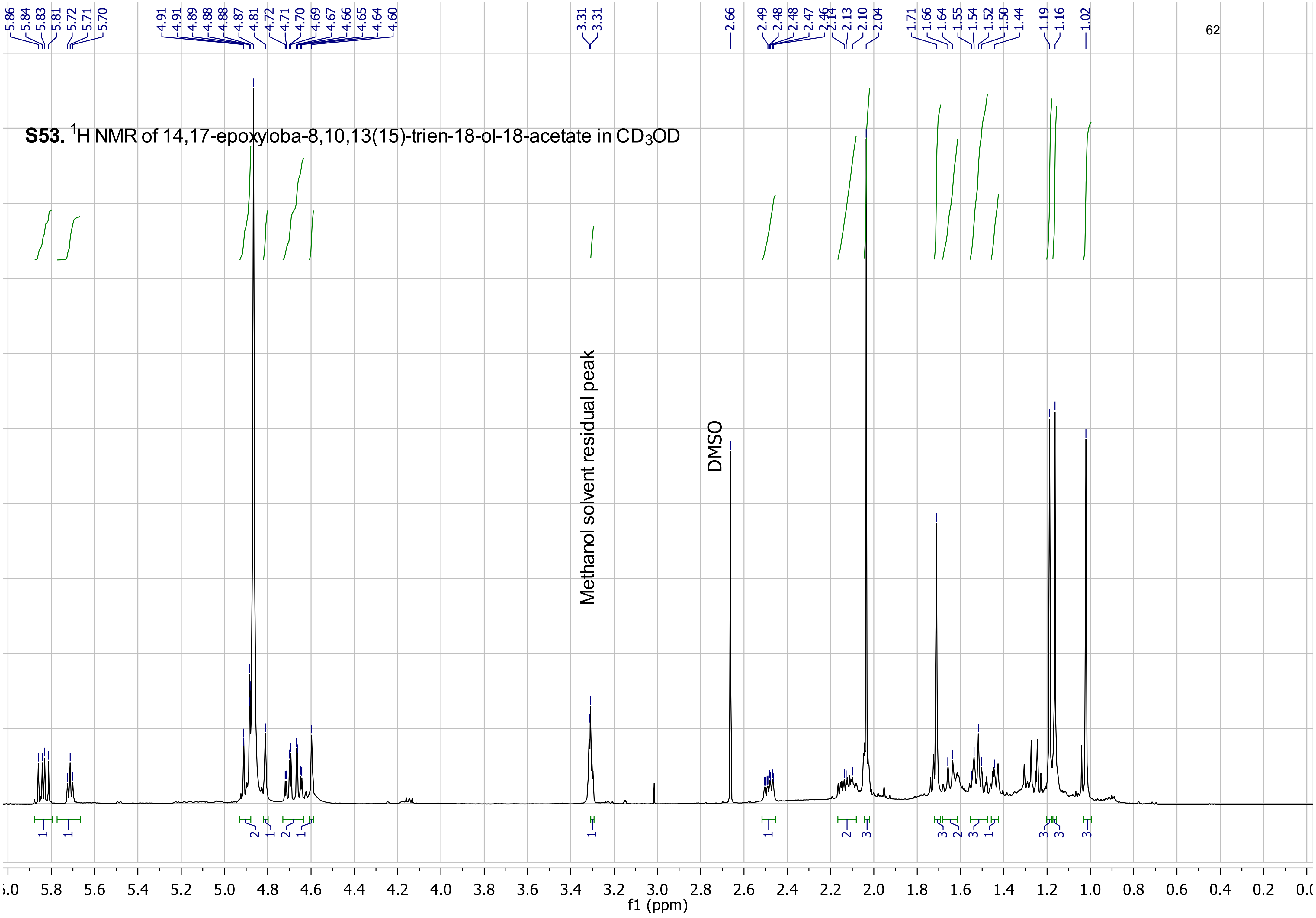

S54. ^{13}C NMR of 14,17-epoxyloba-8,10,13(15)-trien-18-ol-18-acetate in CD_3OD

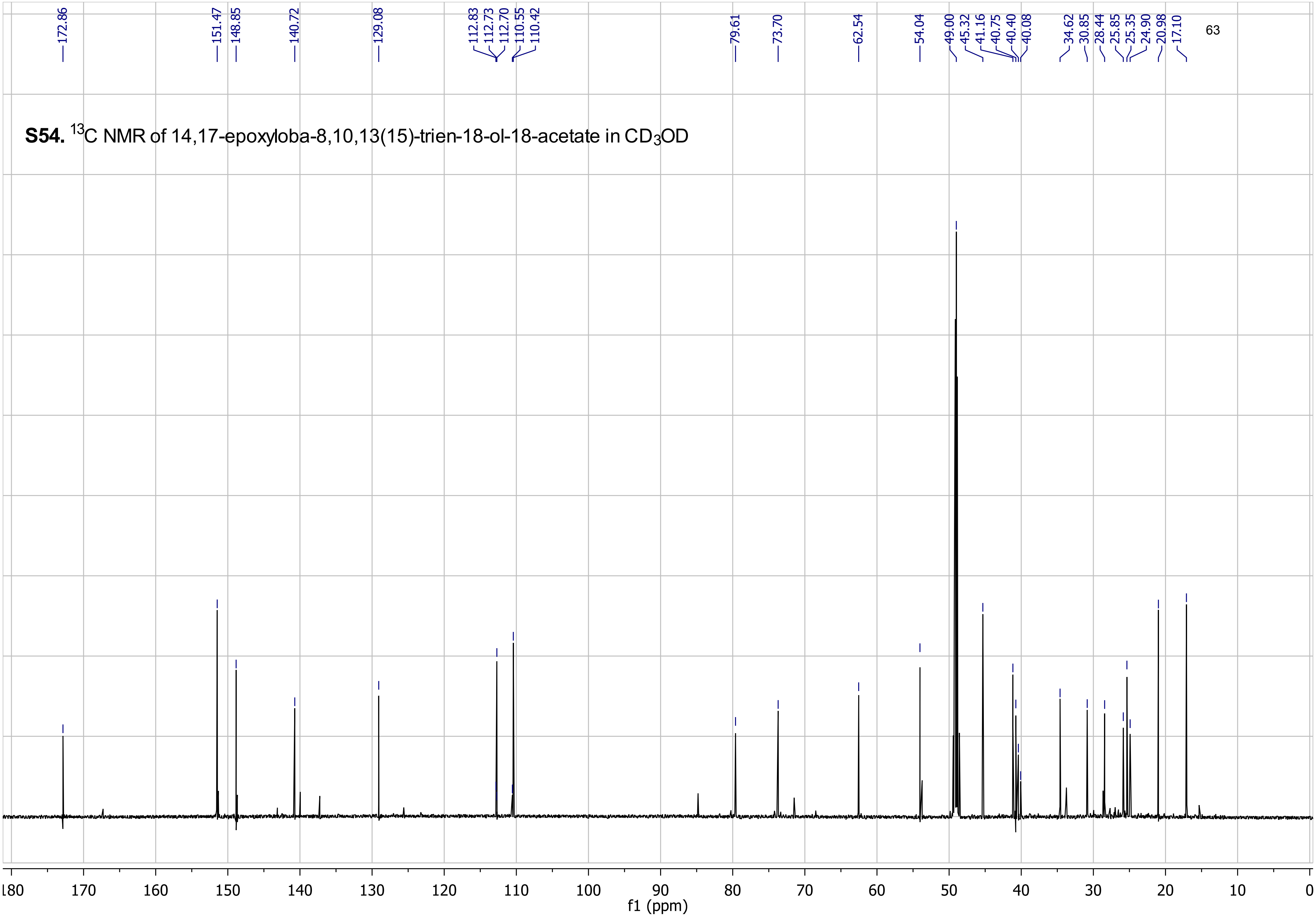

S55. DEPT NMR of 14,17-epoxyloba-8,10,13(15)-trien-18-ol-18-acetate in CD_3OD

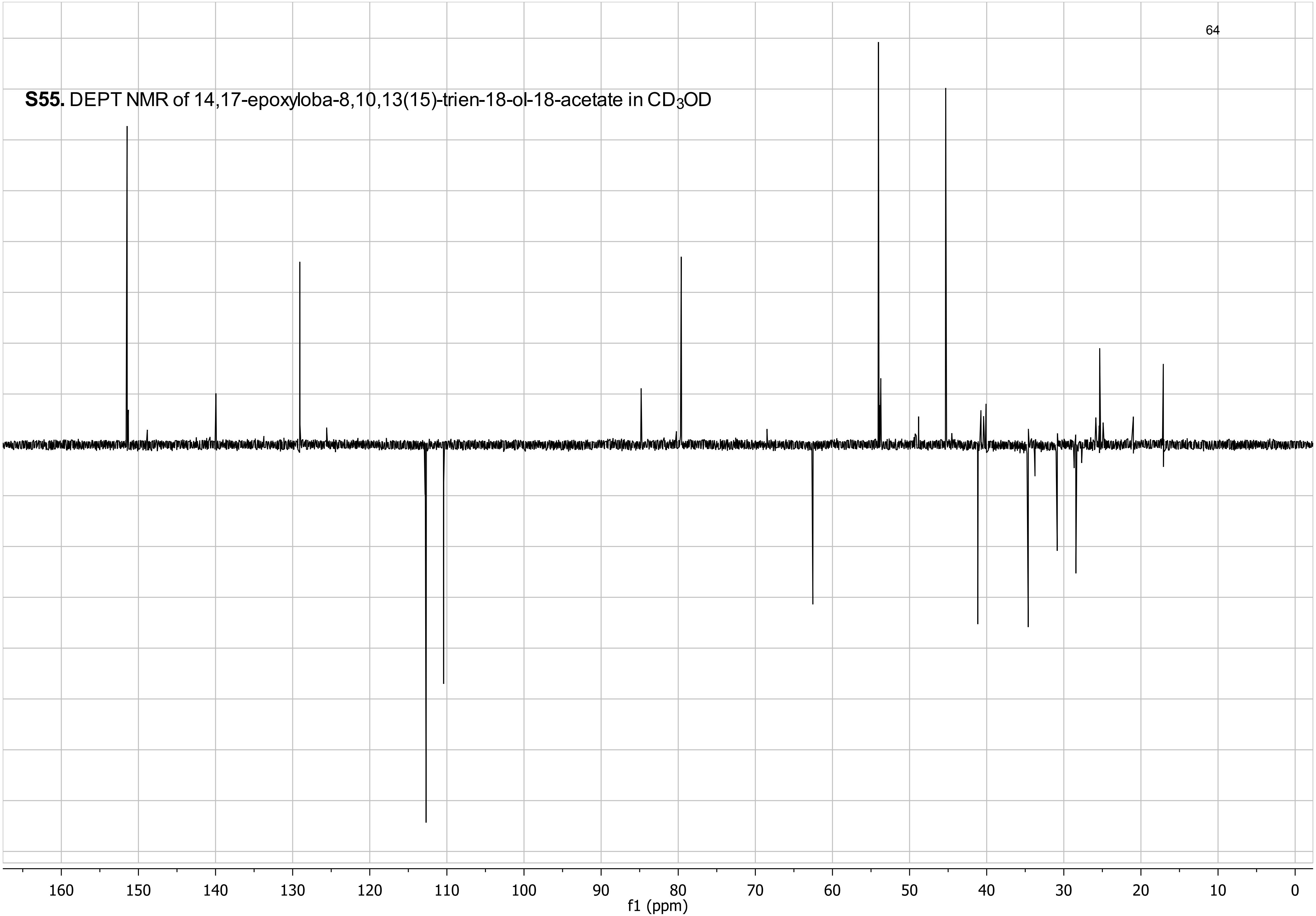

S56. COSY NMR of 14,17-epoxyloba-8,10,13(15)-trien-18-ol-18-acetate in CD_3OD

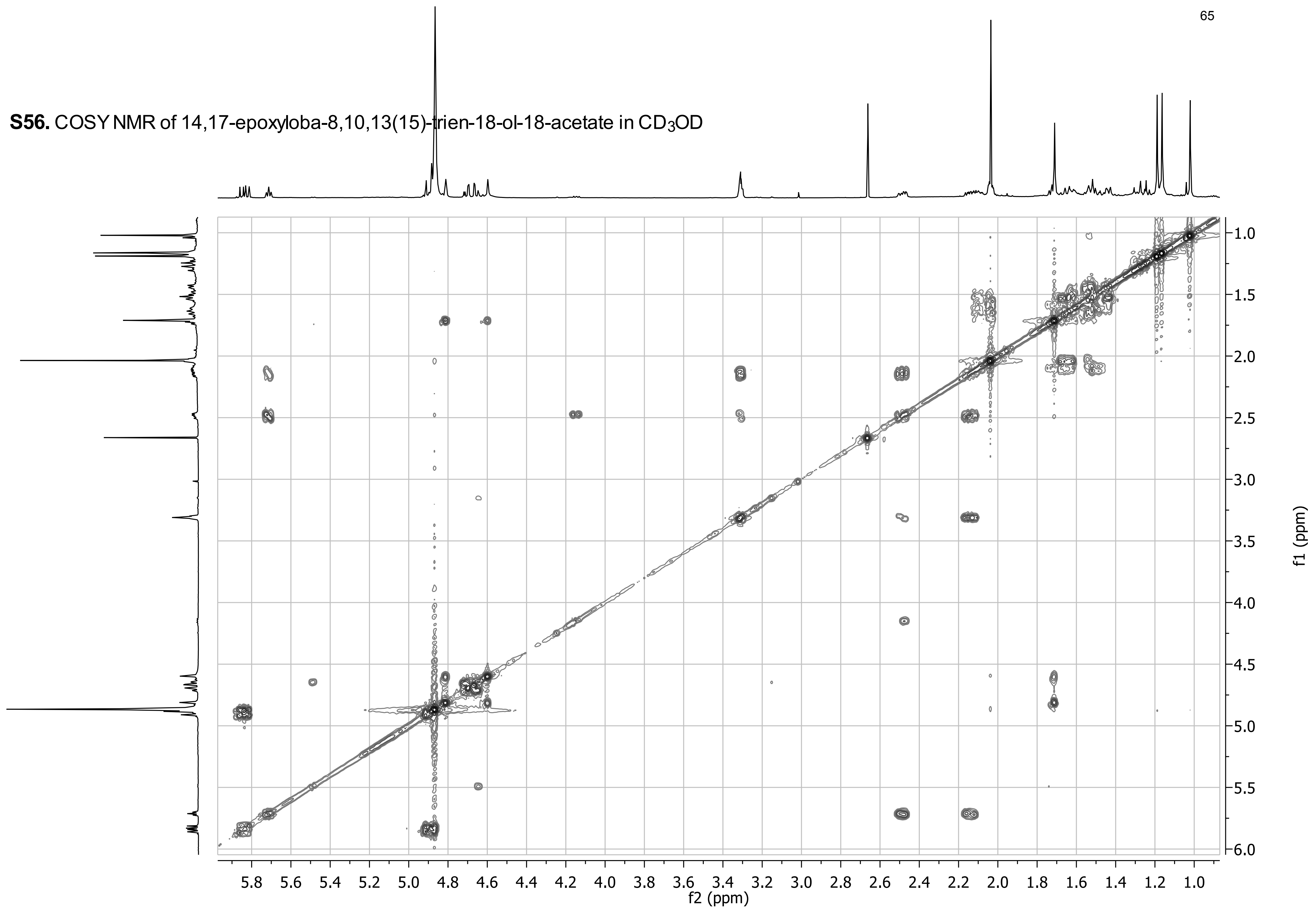

S57. HSQC NMR of 14,17-epoxyloba-8,10,13(15)-trien-18-ol-18-acetate in CD_3OD

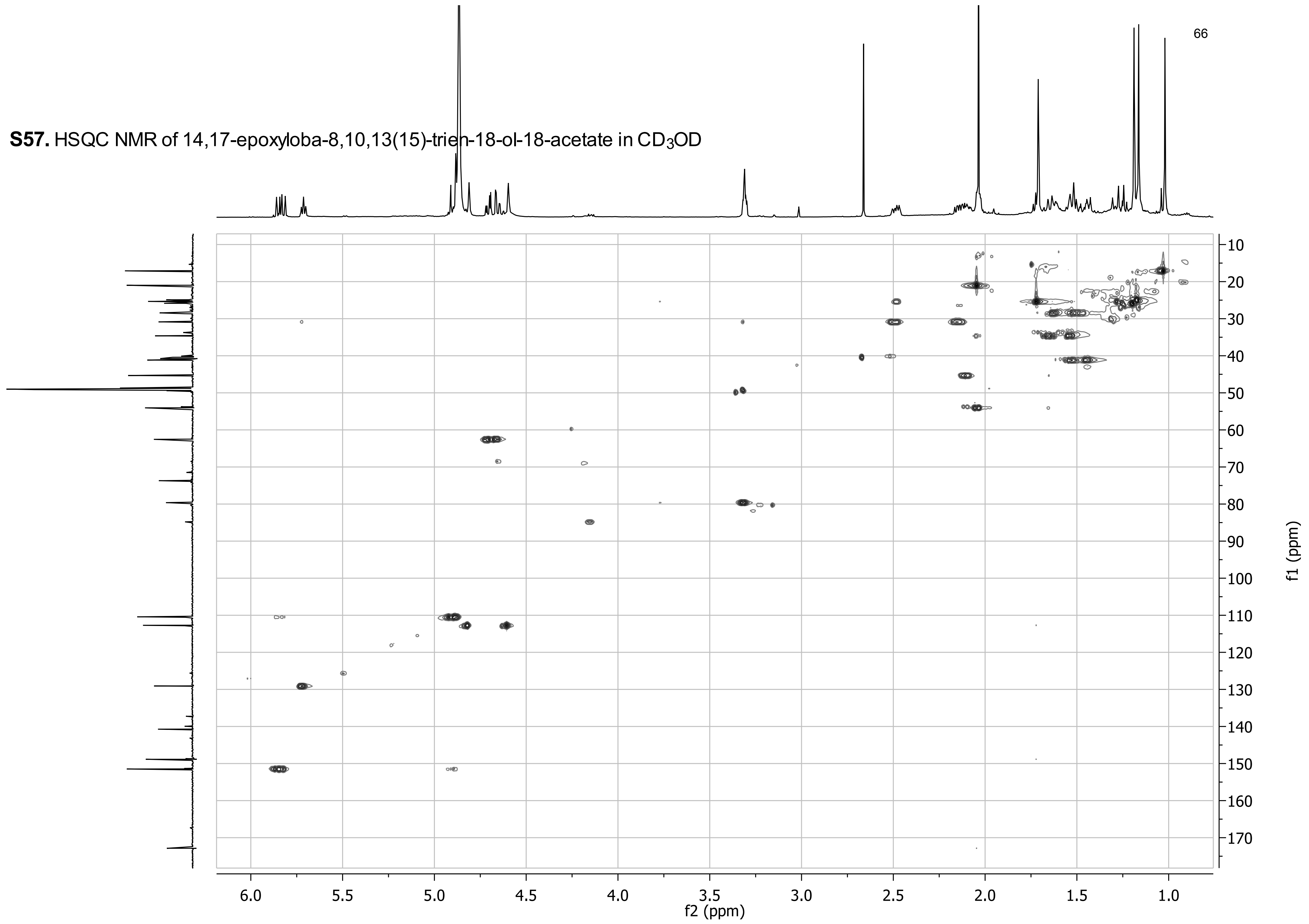

S58. HMBC NMR of 14,17-epoxyloba-8,10,13(15)-trien-18-ol-18-acetate in CD_3OD

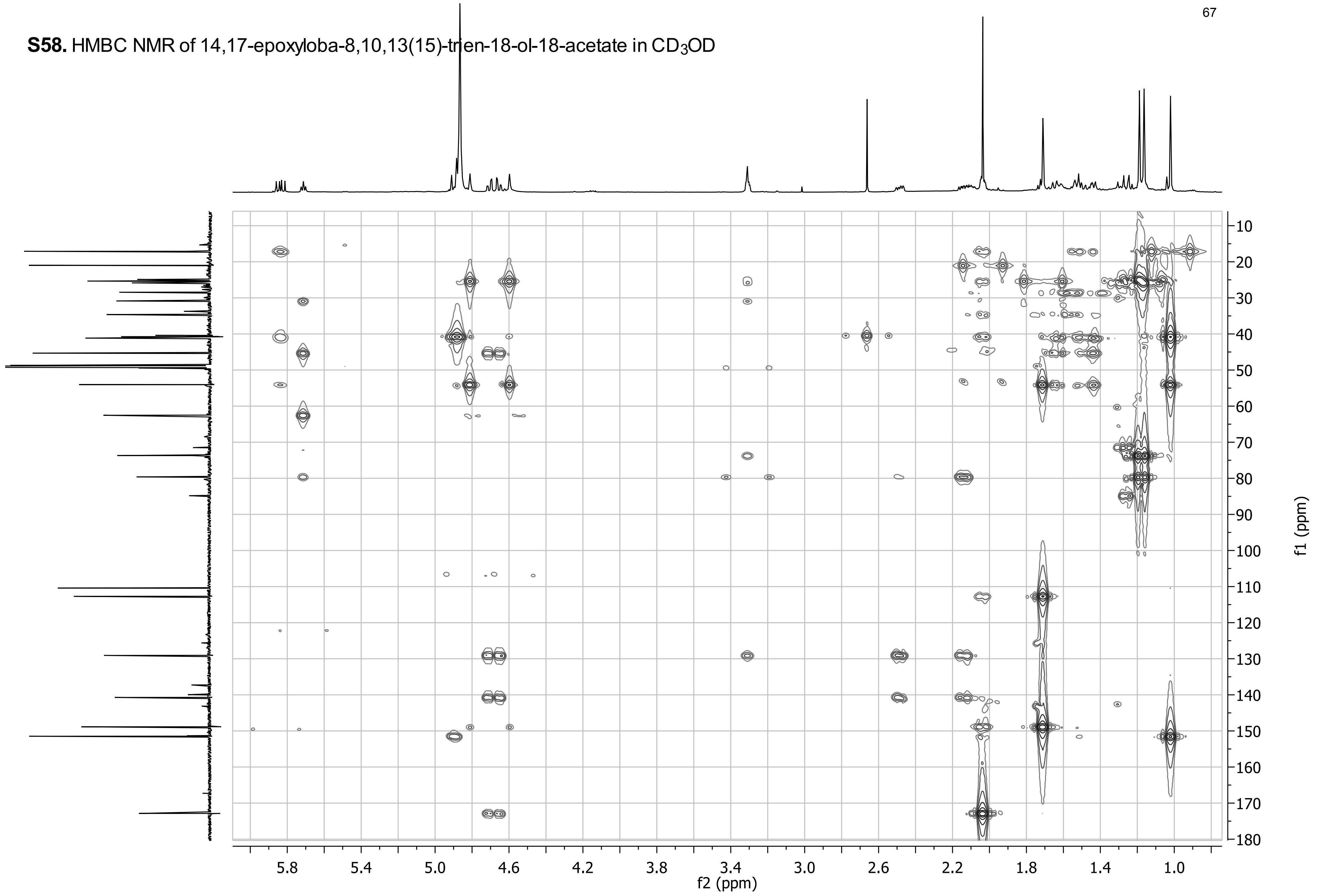

Table S5. ^{1}H and ^{13}C NMR data (300 MHz and 75 MHz, CD_3OD) for 14,17-epoxyloba-8,10,13(15)-trien-18-ol-18-acetate.

No.	^{13}C δ (m)	^{1}H δ (m, J Hz)	COSY	gHMBC
1	40.8 (s)			
2	54.0 (d)	2.04 (1H, m)	H_a-3, H_b-3	C-1, C-3, C-5, C-6, C-7, C-10, C-11, C-12
3	34.6 (t)	1.66 (1H, m)	H_b-3, H4	C-1, C-2, C-4, C-5
		1.54 (1H, dt, 4.0, 6.7)	H-2, H_a-3	C-1, C-2, C-4, C-5, C-7
4	45.3 (d)	2.11 (1H, m)	H-15	C-2, C-13, C-14, C-15
5	28.4 (t)	1.64 (1H, m)	H_b-5, H-6	C-1, C-2, C-4
		1.50 (1H, m)	H_a-5	C-8, C-10
6	41.2 (t)	1.55 (1H, m)	H_a-5	C-1, C-2, C-4, C-5,
		1.44 (1H, m)		C-1, C-2, C-4, C-5, C-7, C-8, C-10, C-13
7	17.1 (q)	1.02 (3H, s)		C-1, C-2, C-8, C-9
8	151.5 (d)	5.84 (1H, 10.8, 17.5)	H-9	C-1, C-2, C-7
9	110.4 (t)	4.91 (1H, dd, 1.4, 17.5)	H-8	C-1, C-2, C-8
		4.89 (1H, t, 1.4, 10.8)	H-8	C-1, C-2, C-8
10	148.9 (s)			
11	112.7 (t)	4.81 (1H, brdq, 1.7, 3.0)	H_b-11, H_3-12	C-1, C-2, C-10, C-12
		4.60 (1H, brdq, 0.8, 3.0)	H_a-11, H_3-12	C-1, C-2, C-10, C-12
12	25.4 (q)	1.71 (3H, brdd, 0.8)	H_a-11, H_b-11	C-2, C-10, C-11
13	141.7 (s)			
14	62.5 (t)	4.71 (1H, dd, 3.7, 12.1)		C-4, C-13, C-15, C-22
		4.66 (1H, dd, 2.6, 12.1)		
15	129.1 (d)	5.71 (1H, brt, 7.2)	H_a-16, H_b-16	C-4, C-13, C-14, C-16, C-17
16	30.9 (t)	2.49 (1H, ddd, 2.4, 7.2, 15.2)	H-15	C-4, C-13, C-14, C-15, C-17, C-18
		2.13 (1H, ddd, 7.2, 10.1, 15.2)	H-14, H-15, H_a-16	C-13, C-14, C-15, C-18
17	79.6 (d)	3.31 (1H, dd, 2.4, 10.1)	H_a-16, H_b-16	C-15, C-16, C-18, C-19, C-20
18	73.7 (s)			
19	24.9 (q)	1.16 (3H, s)		C-14, C-18, C20
20	25.9 (q)	1.19 (3H, s)		C-14, C-18, C-19
OAc	172.9 (s)			
OAcMe	21.0 (q)	2.04 (3H, s)		C-22

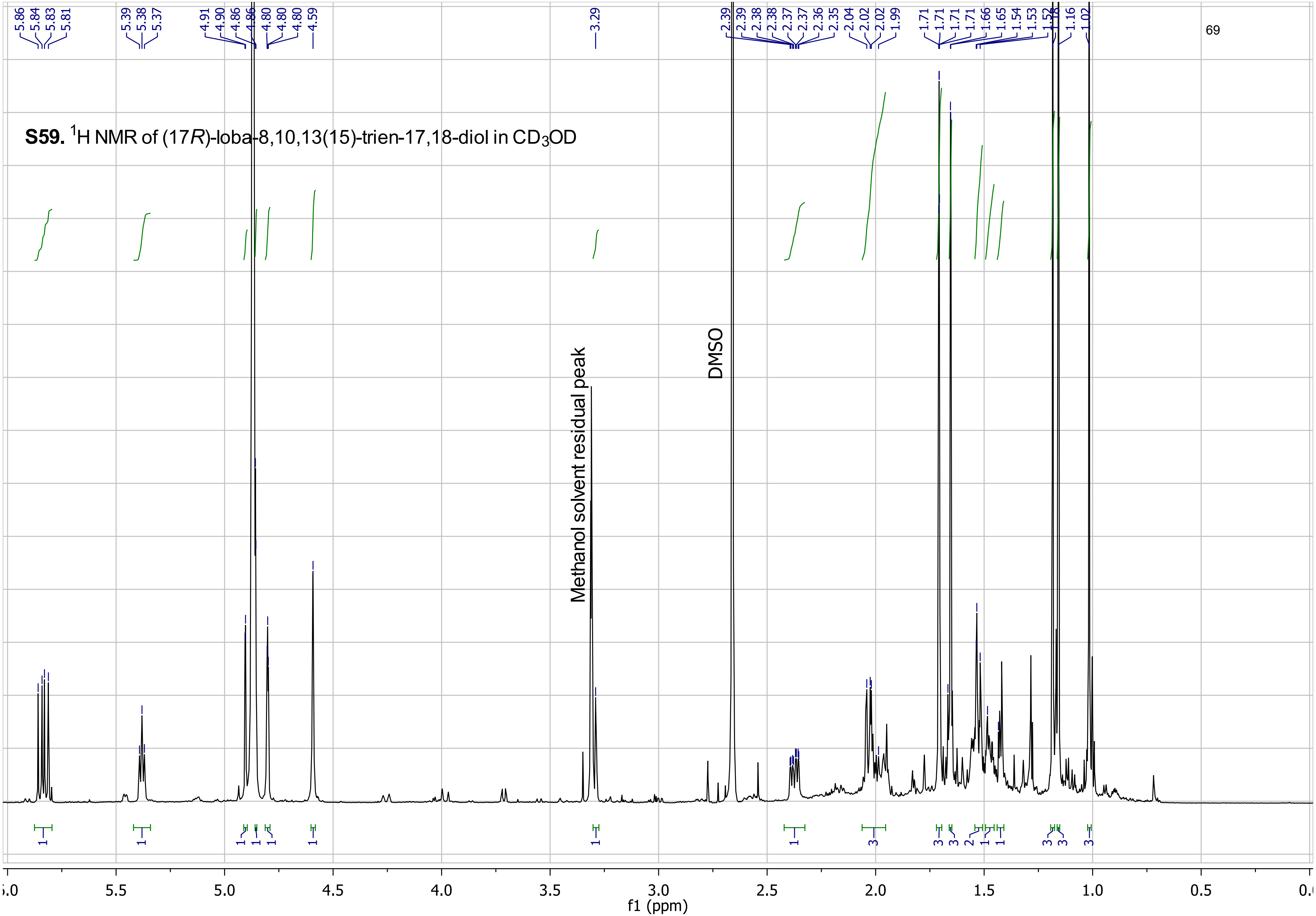

S59. ^{1}H NMR of (17*R*)-loba-8,10,13(15)-trien-17,18-diol in CD_3OD

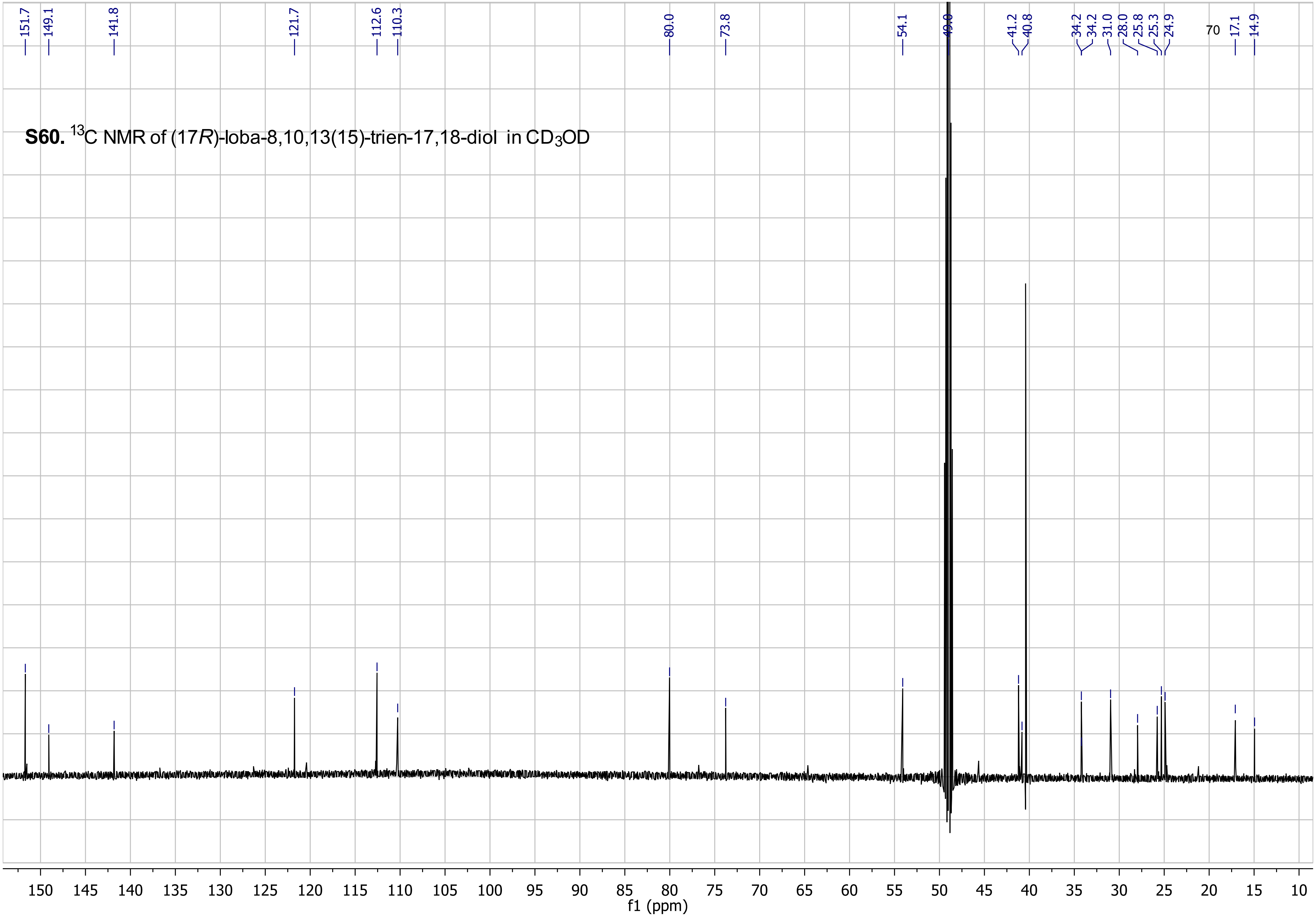

S60. ^{13}C NMR of (17*R*)-loba-8,10,13(15)-trien-17,18-diol in CD_3OD
151.7
149.1
141.8
121.7
112.6
110.3
80.0
73.8
54.1
49.0
41.2
40.8
34.2
34.2
31.0
28.0
25.8
25.3
24.9
17.1
14.9
150 145 140 135 130 125 120 115 110 105 100 95 90 85 80 75 70 65 60 55 50 45 40 35 30 25 20 15 10
f1 (ppm)

S61. COSY NMR of (17*R*)-loba-8,10,13(15)-trien-17,18-diol in CD_3OD

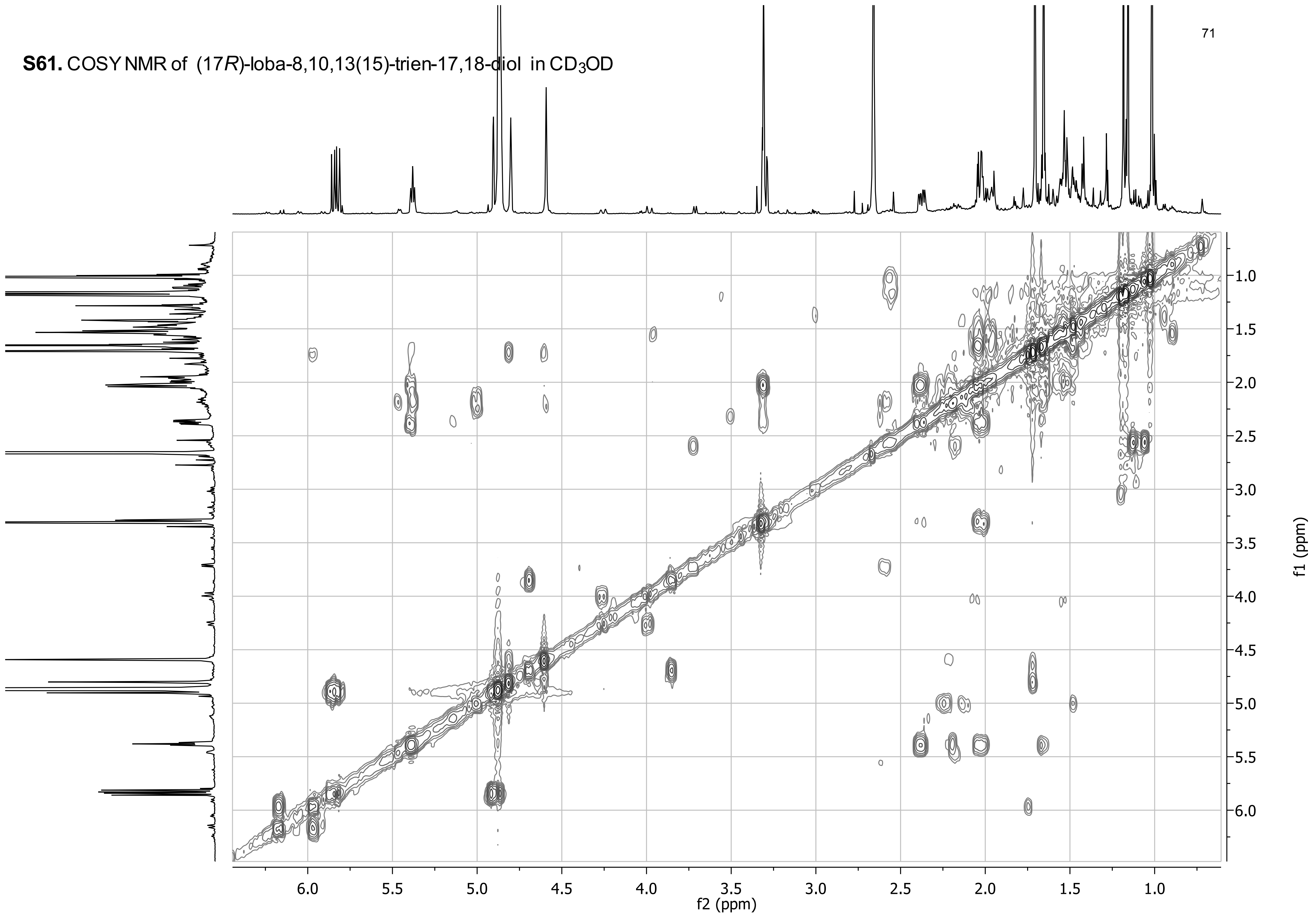

S62. HSQC NMR of (17*R*)-loba-8,10,13(15)-trien-17,18-diol in CD_3OD

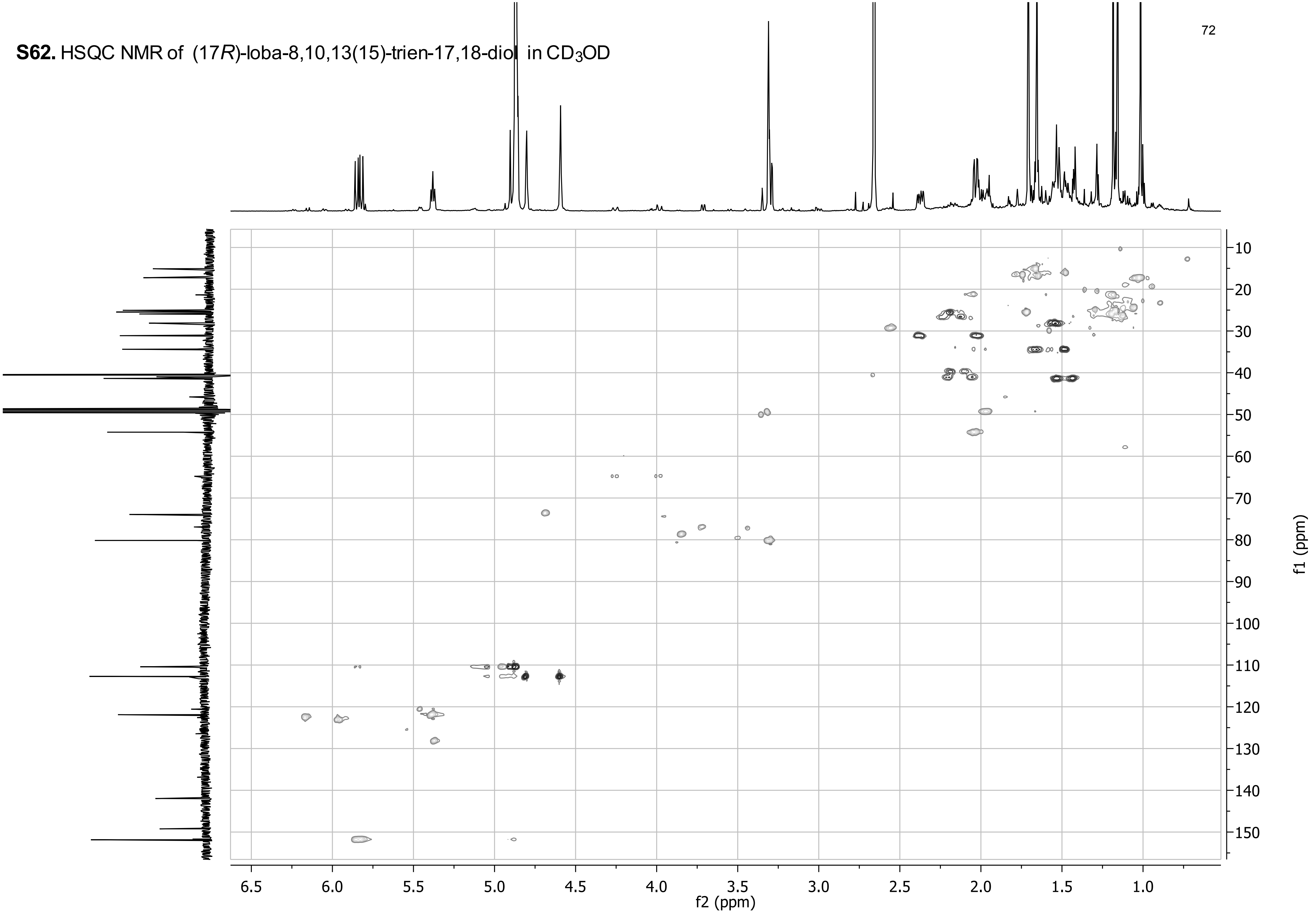

S63. HMBC NMR of (17*R*)-loba-8,10,13(15)-trien-17,18-diol in CD_3OD

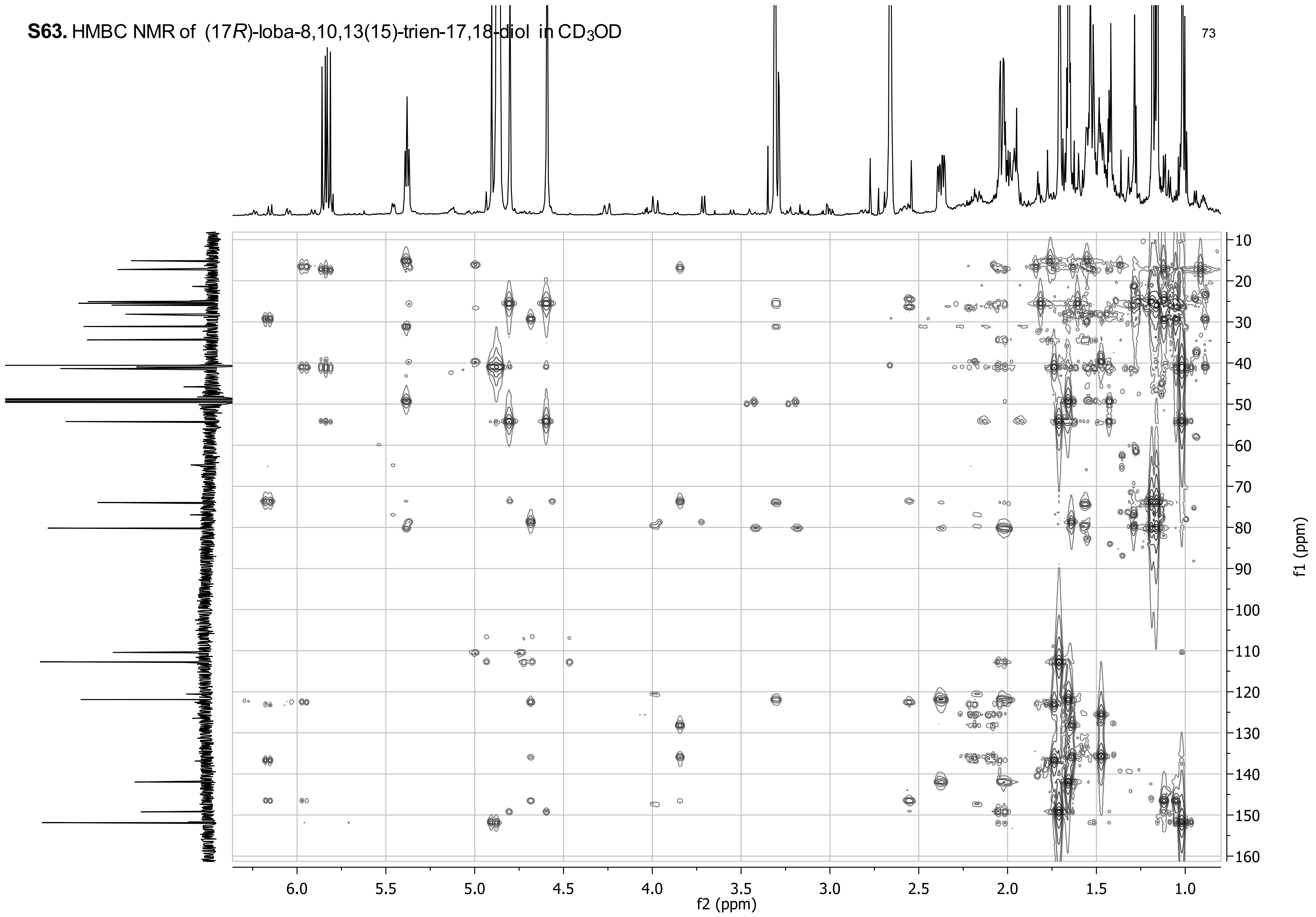

Table S6. ^{1}H and ^{13}C NMR data (300 MHz and 75 MHz, CD_3OD) for (17*R*)-loba-8,10,13(15)-trien-17,18-diol.

No.	^{13}C δ (m)	^{1}H δ (m, J Hz)	COSY	gHMBC
1	40.8 (s)			
2	54.1(d)	2.03 (1H, dd, 3.1, 12.6)	H-3	C-1, C-2, C-4, C-7, C-10, C-11, C-12
3	34.2 (t)	1.67 (1H, m)	H-2, H_b-3, H-4	C-1, C-2, C-4, C-5
		1.48 (1H, m)	H_a-3	C-2, C-5
4	49.3 (d)	1.98 (1H, m)	H_a-3, H-5	C-3, C-6, C-13, C-15
5	28.0 (t)	1.54 (2H, m)	H-4, H_b-6	C-3, C-6, C-4, C-14
6	41.2 (t)	1.53 (1H, m)	H_b-6	C-5, C-7, C-8
		1.43 (1H, m)	H-4, H_a-6	C-1, C-2, C-4, C-7, C-8, C-10
7	17.1 (q)	1.02 (3H, s)		C-1, C-2, C-6, C-8
8	151.7 (d)	5.84 (1H, 10.8, 17.6)	H_a-9	C-1, C-2, C-6, C-7, C-8
9	110.3 (t)	4.90 (1H, d, 1.4, 17.6)	H-8, H_b-9	C-1, C-2, C-8
		4.86 (1H, t, 1.4, 10.8)	H-8, H_b-9	C-1, C-2, C-8
10	149.1 (s)			
11	112.6 (t)	4.80 (1H, dq, 1.4, 3.2)	H_b-11, H_3-12	C-1, C-2, C-10, C-12
		4.59 (1H, brs)	H_a-11, H_3-12	C-1, C-2, C-10, C-12
12	25.3 (q)	1.71 (3H, brdd, 0.8, 1.4)	H_a-11, H_b-11	C-1, C-2, C-10, C-11
13	141.8 (s)			
14	15.0 (q)	1.66 (3H, brd, 0.8)	H-15	C-4, C-13, C-15
15	121.7(d)	5.38 (1H, ddq, 0.8, 6.3, 7.3)	H-4, H-14, H-16, H-17	C-4, C-14, C-16, C-17
16	31.0 (t)	2.37 (1H, ddd, 2.4, 7.3, 14.8)	H-15, H_b-16, H-17	C-13, C-15, C-17, C-18
		2.03 (1H, m)	H-15, H_a-16, H-17, H-14	C-13, C-15, C-17, C-18
17	80.0 (d)	3.29 (1H, d, 2.4)	H_a-16, H_b-16	C-15, C-16, C-18, C-19, C-20
18	73.8 (s)			
19	24.9 (q)	1.16 (3H, s)		C-17, C-18
20	25.8 (q)	1.19 (3H, s)		C-17, C-18

S64. ^{1}H NMR of sarcophytol-B in CD_3OD

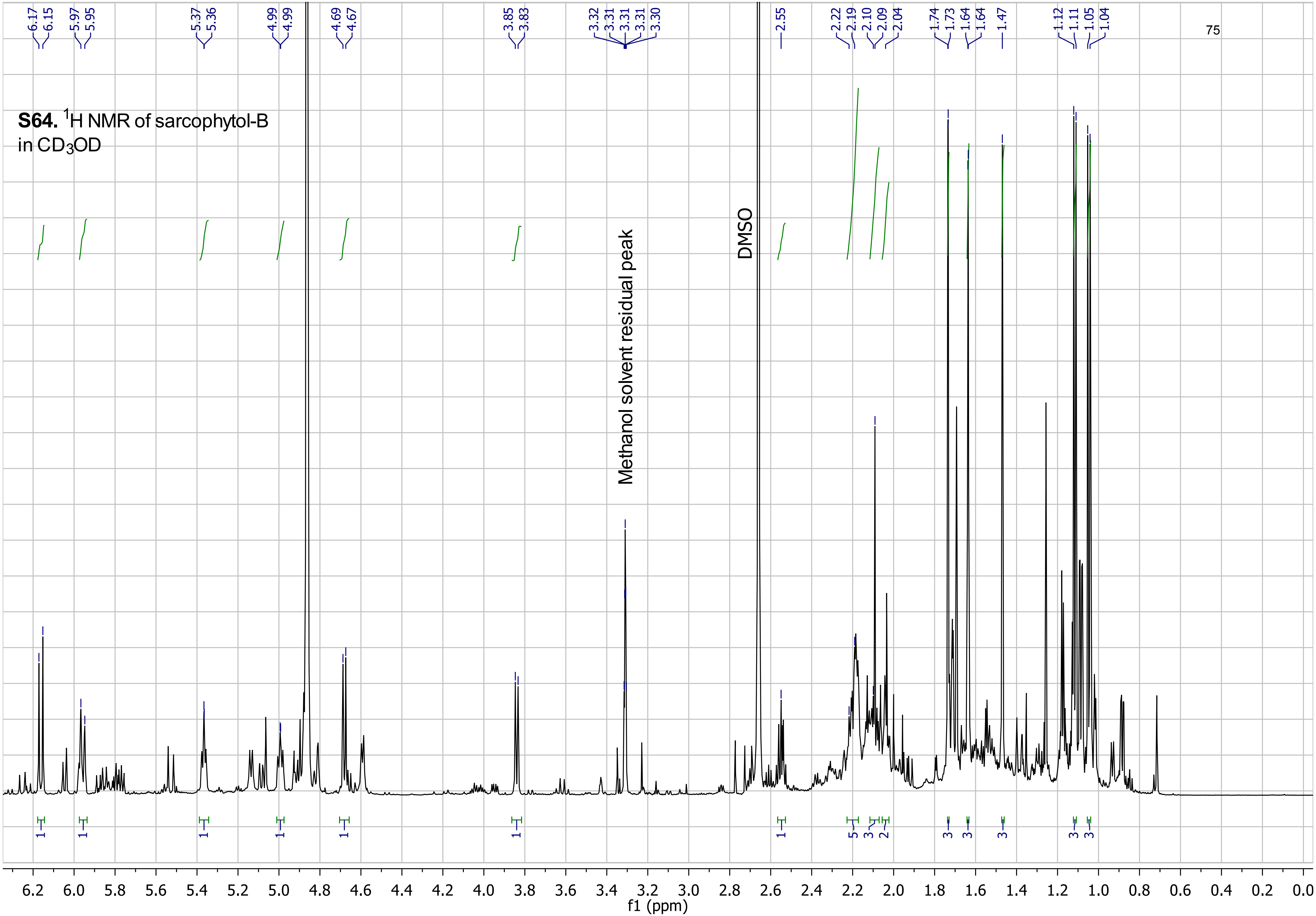

S65. ^{13}C NMR of sarcophytol-B in CD_3OD

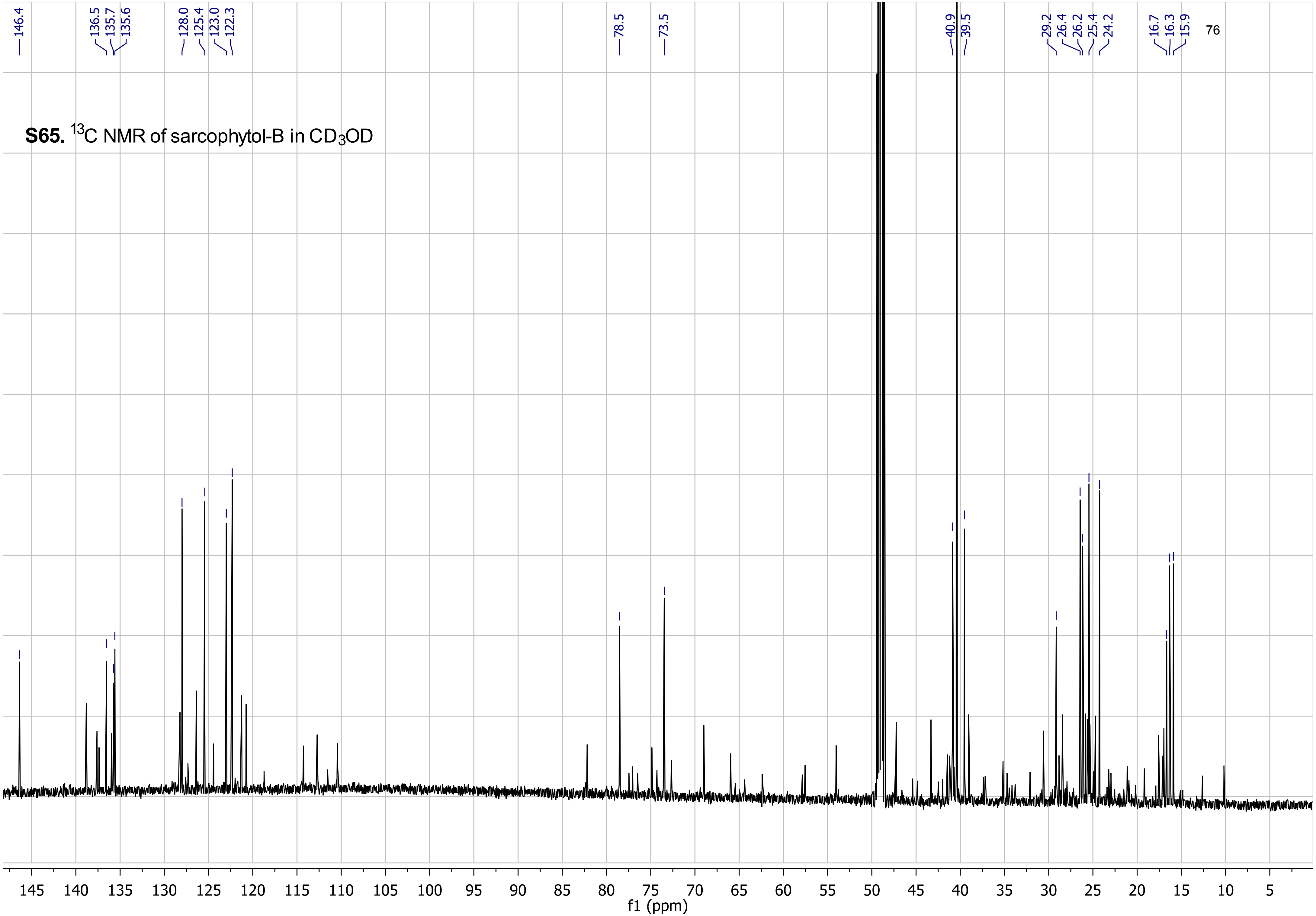

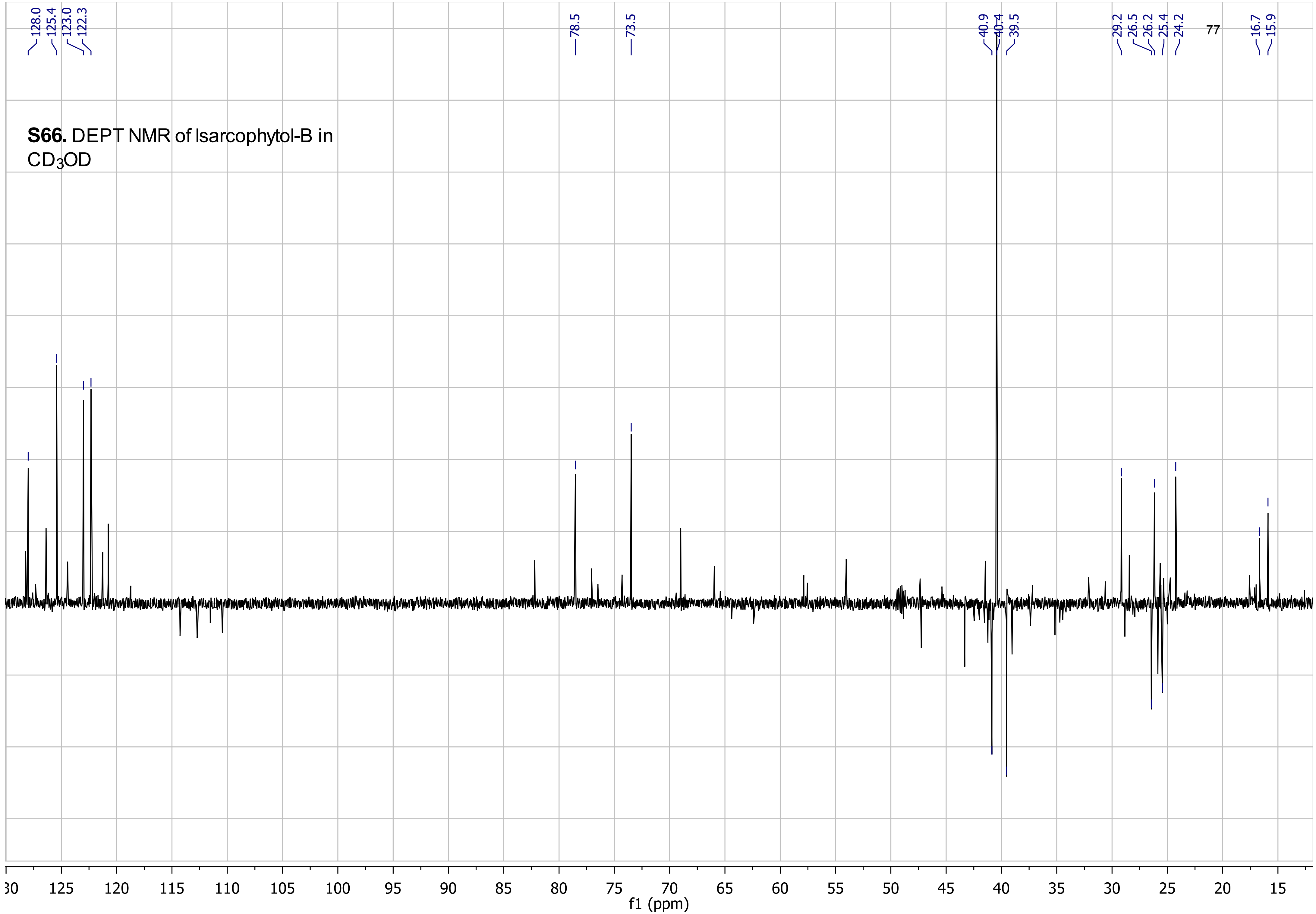

S66. DEPT NMR of Isarcophytol-B in CD_3OD

S67. COSY NMR of sarcophytol-B in CD_3OD

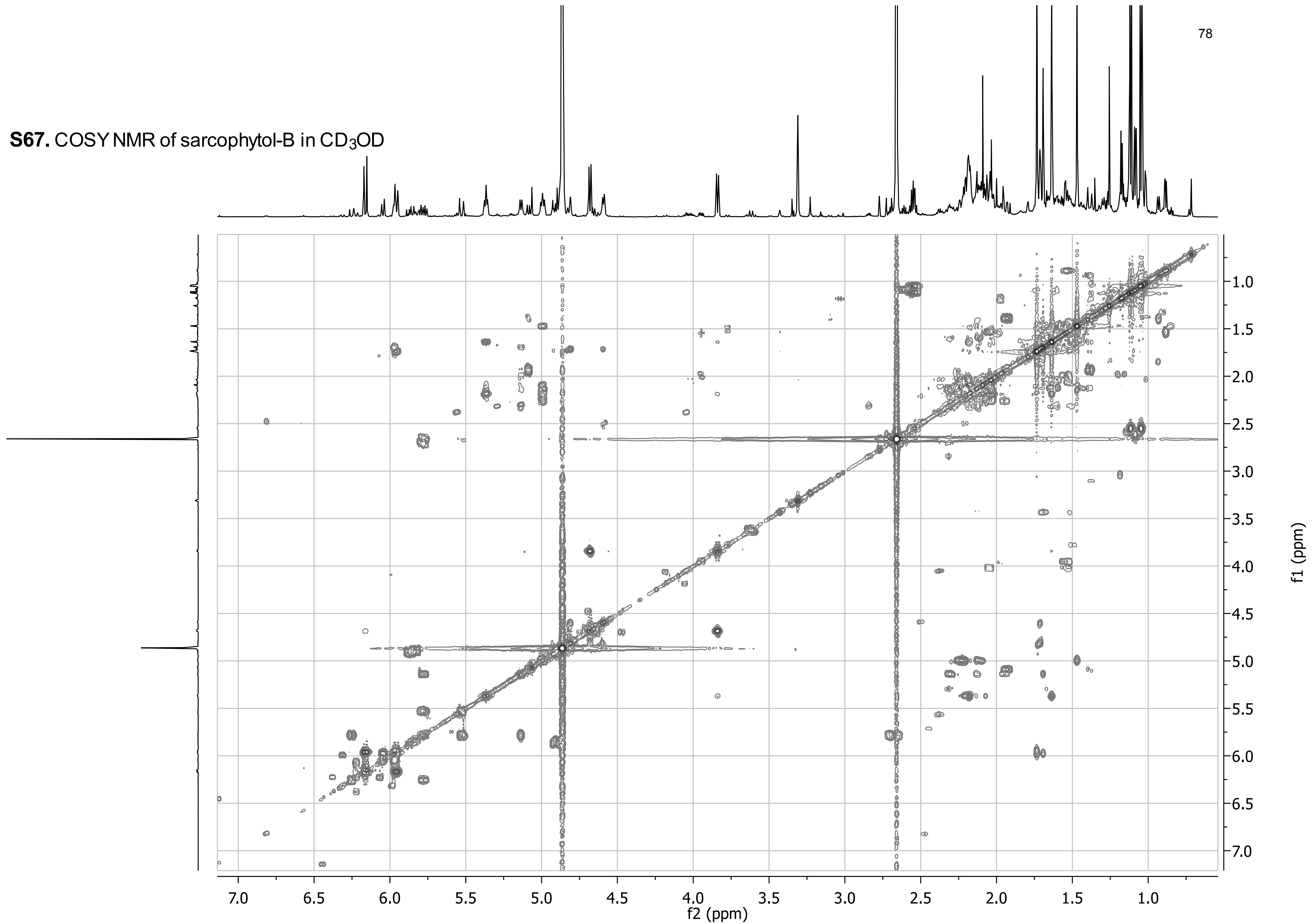

S68. HSQC NMR of sarcophytol-B in CD_3OD

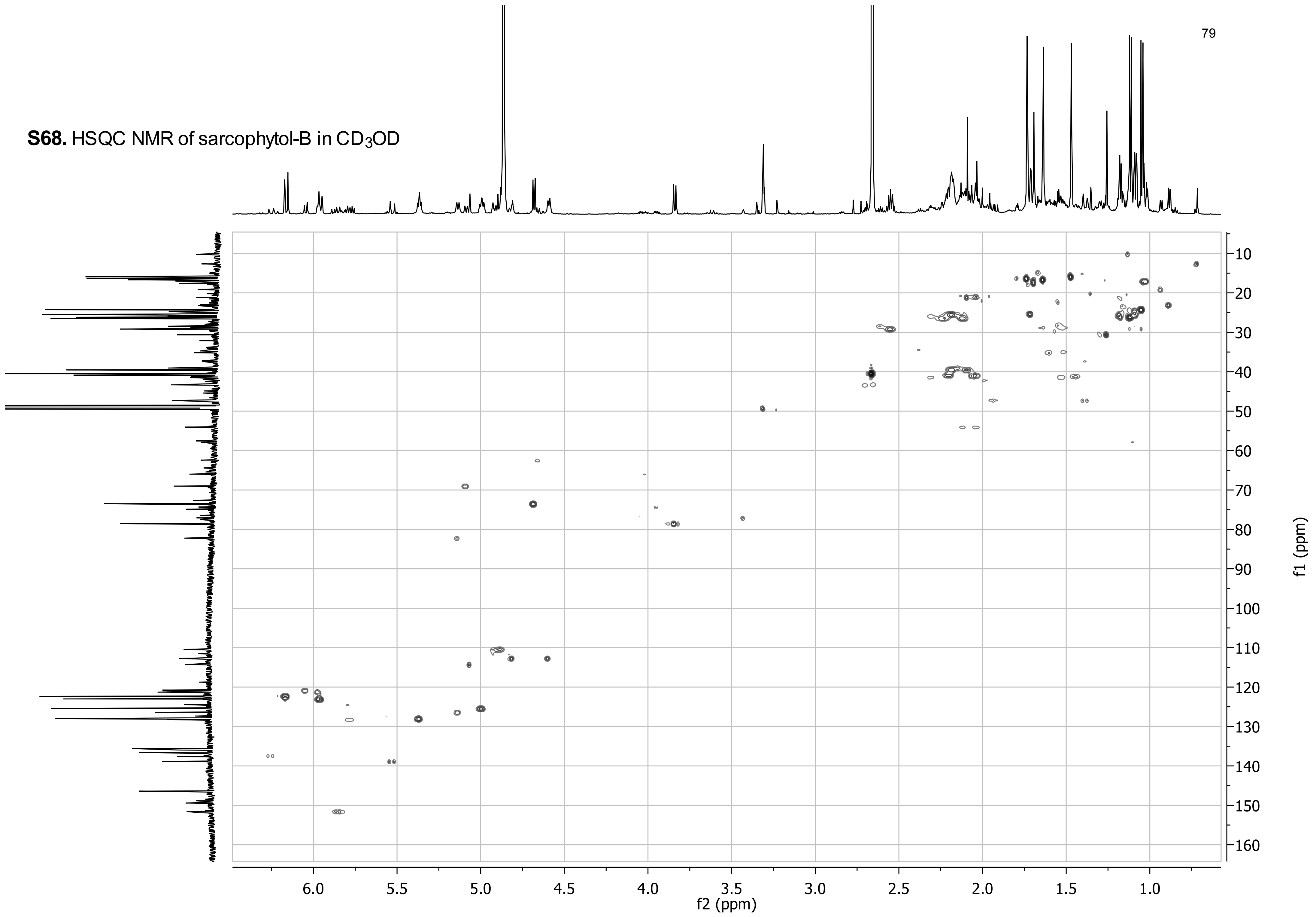

S69. HMBC NMR of sarcophytol-B in CD_3OD

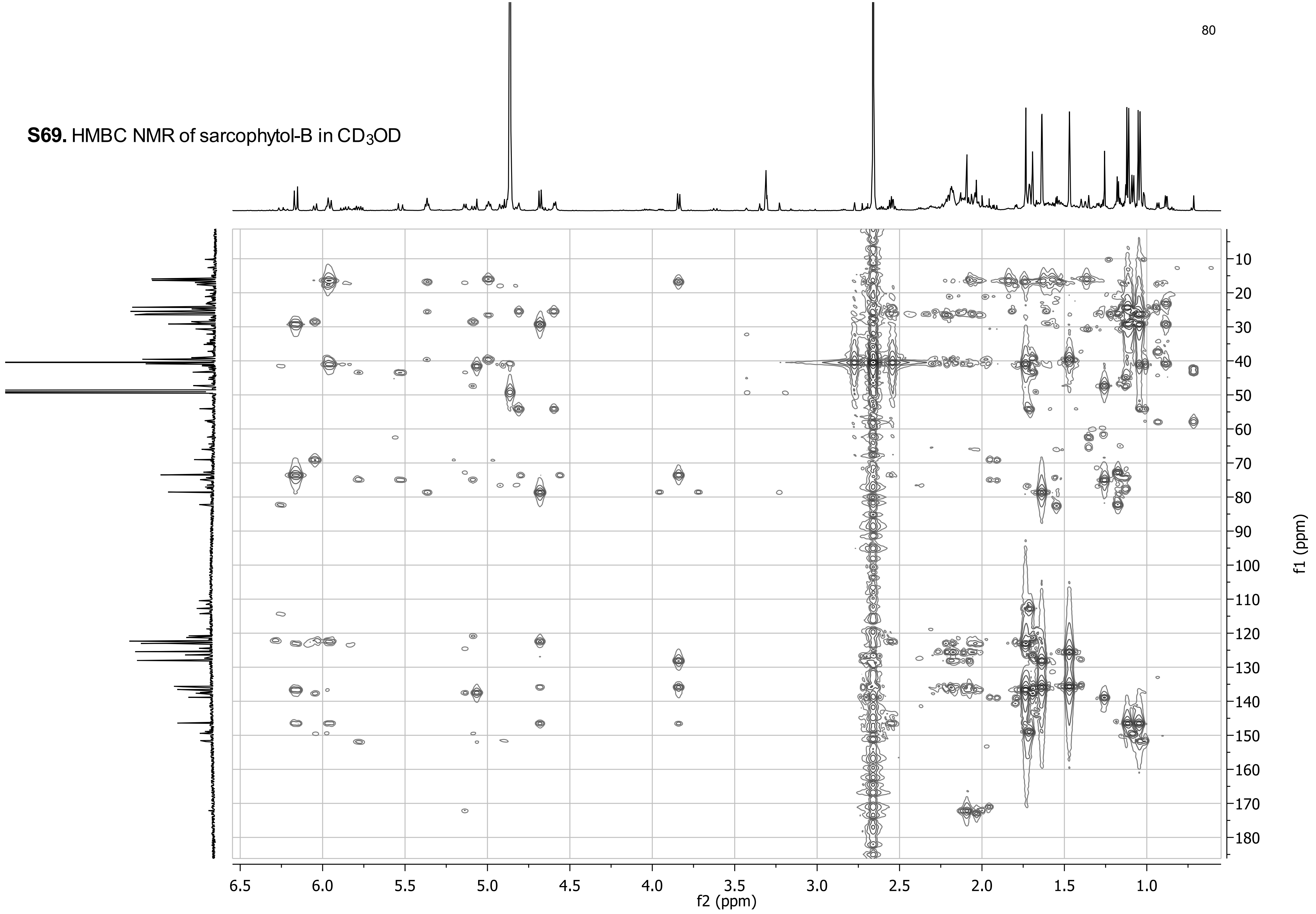

Table S7. ^{1}H and ^{13}C NMR data (300 MHz and 75 MHz, CD_3OD) for sarcophytol-B.

No.	^{13}C δ (m)	^{1}H δ (m, J Hz)	COSY	gHMBC
1	146.4 (s)			
2	122.3(d)	6.16 (1H, d, 11.5)	H-3, H-13	C-1, C-4, C-14, C-15
3	123.0 (d)	5.96 (1H, d, 11.5)	H-2, H-18	C-1, C-2, C-5, C-18
4	136.5 (s)			
5	26.6 (t)	2.21 (1H, m)	H_b-5, H_b-6	C-3, C-6
		2.05 (1H, m)	H-7, H_a-5	C-3, C-4, C-6, C-18
6	40.9 (t)	2.23 (1H, m)	H_b-6, H-7	C-7, C-8
		2.11 (1H, m)	H-7, H_a-5, H_a-6	C-5, C-7, C-8
7	125.4 (d)	4.99 (1H, ddq, 1.1, 6.5, 8.5)	H_a-6, H_b-6, H_3-19	C-6, C-10, C-19
8	135.6 (s)			
9	39.5 (t)	2.19 (1H, m)	H_b-9	C-7, C-8
		2.09 (1H, m)	H-10, H_a-9	C-8, C-11, C-19
10	25.8 (t)	2.18 (2H, m)	H_b-9, H-11	C-11
11	128.0 (d)	5.37 (1H, tq, 1.0, 11.9)	H_a-10, H_b-10, H-13, H_3-20	C-9, C-10, C-13, C-21
12	135.7 (s)			
13	78.5 (d)	3.84 (1H, d, 8.2)	H-11, H-14	C-1, C-11, C-12, C-14, C-20
14	73.5 (d)	4.68 (1H, d, 8.2)	H-2, H-13	C-1, C-2, C-12, C-13, C-15
15	29.2(d)	2.54 (1H, dq, 6.8, 13.7)	H-16, H-17	C-1,C-2, C-14, C-16, C-17
16	24.2 (q)	1.05 (3H, d, 6.8)	H-15	C-1, C-15, C-17
17	26.2 (q)	1.12 (3H, d, 6.8)	H-15	C-1, C-15, C-16
18	16.3 (q)	1.73 (3H, d, 1.0)	H-3	C-3, C-4, C-5
19	15.9(q)	1.47 (3H, brs)	H-7	C-7, C-8, C-9
20	16.7 (q)	1.64 (3H, d, 1.1)	H-11	C-11, C-12, C-13

S70. ^{1}H NMR of 1(*E*),3(*E*),7(*E*)-11,12-epoxycembratrien-15-ol in CD_3OD

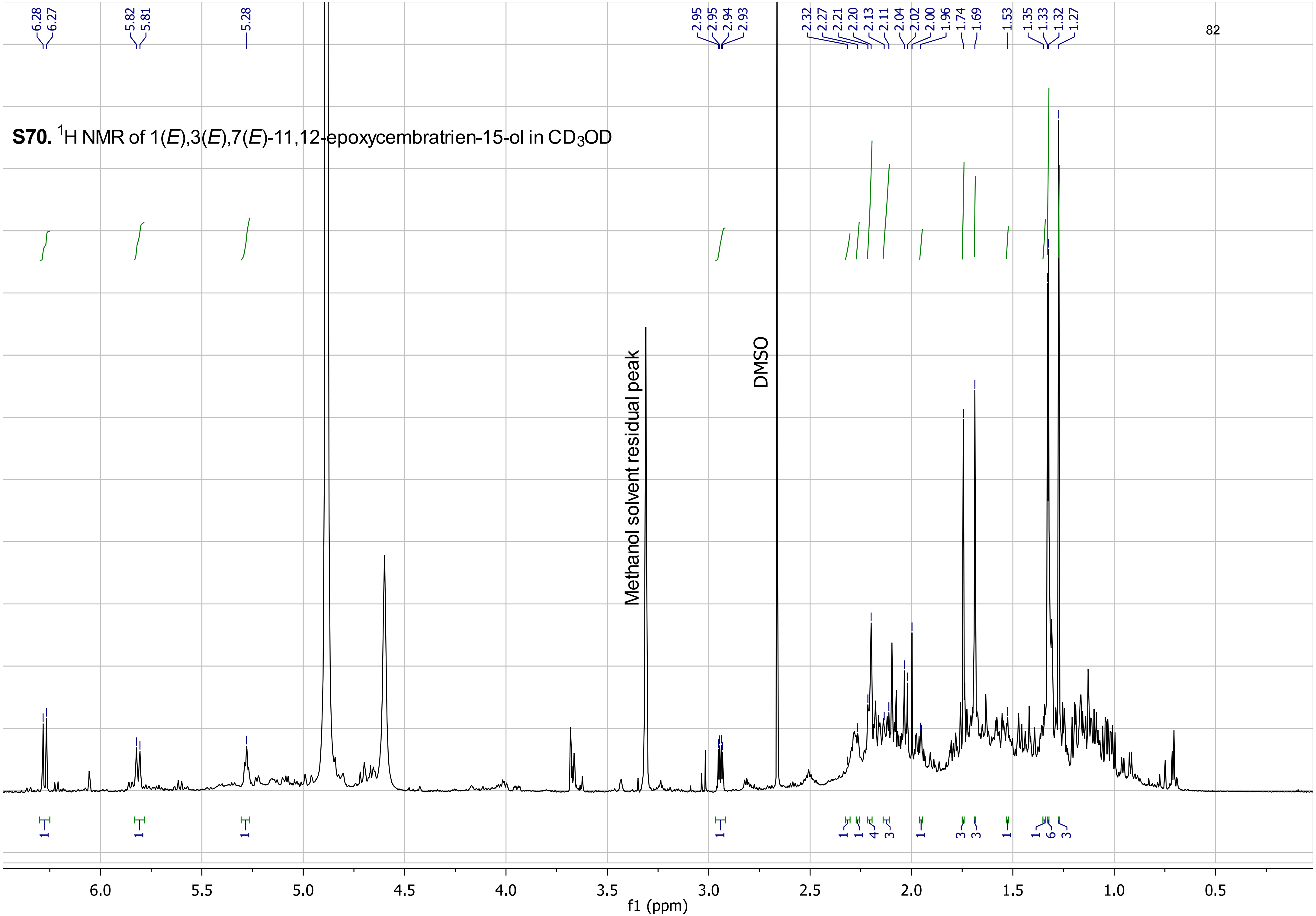

S71. ^{13}C NMR of 1(*E*),3(*E*),7(*E*)-11,12-epoxycembratrien-15-ol in CD_3OD

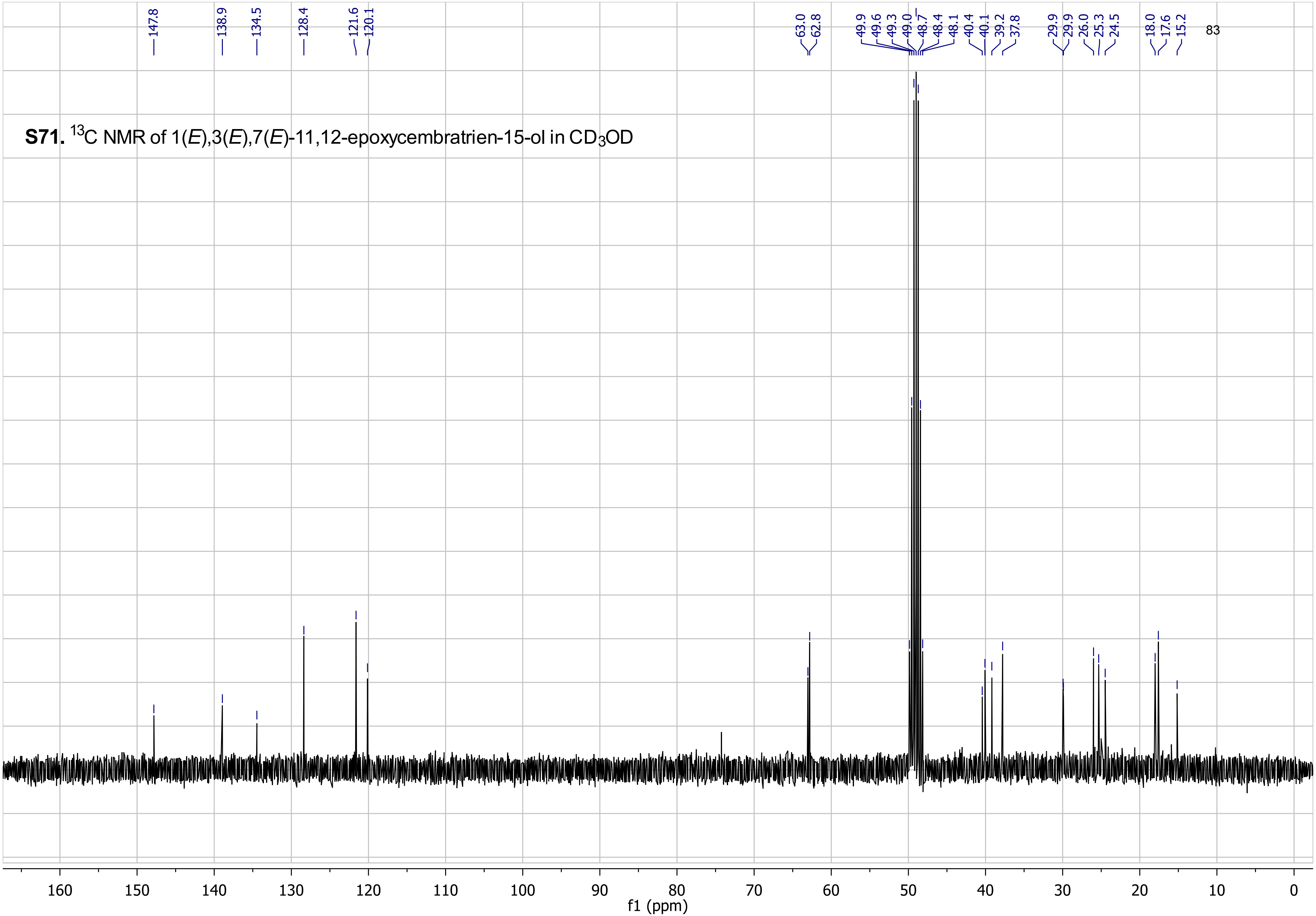

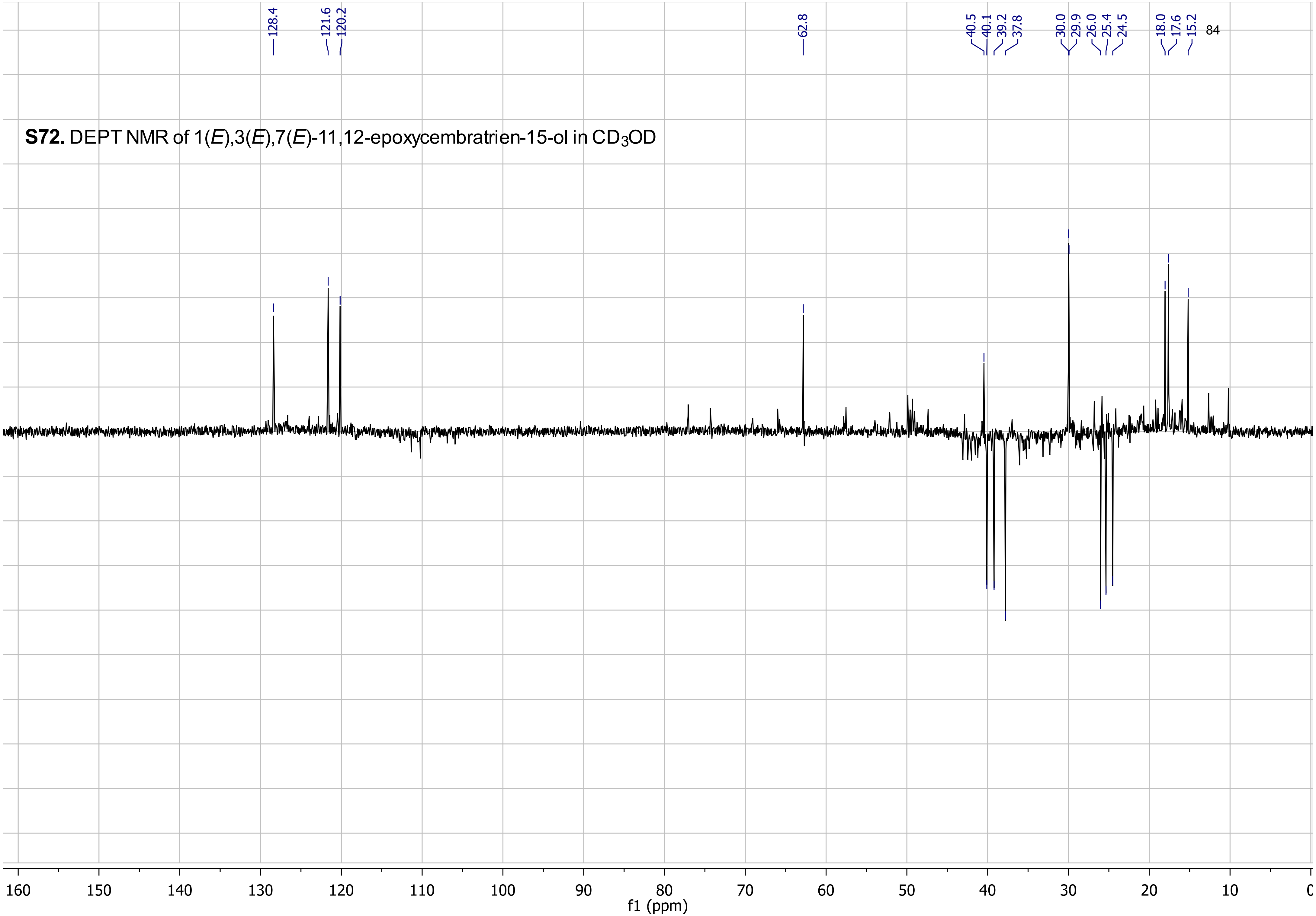

S72. DEPT NMR of 1(*E*),3(*E*),7(*E*)-11,12-epoxycembratrien-15-ol in CD_3OD

S73. COSY NMR of 1(*E*),3(*E*),7(*E*)-11,12-epoxycembratrien-15-ol in CD_3OD

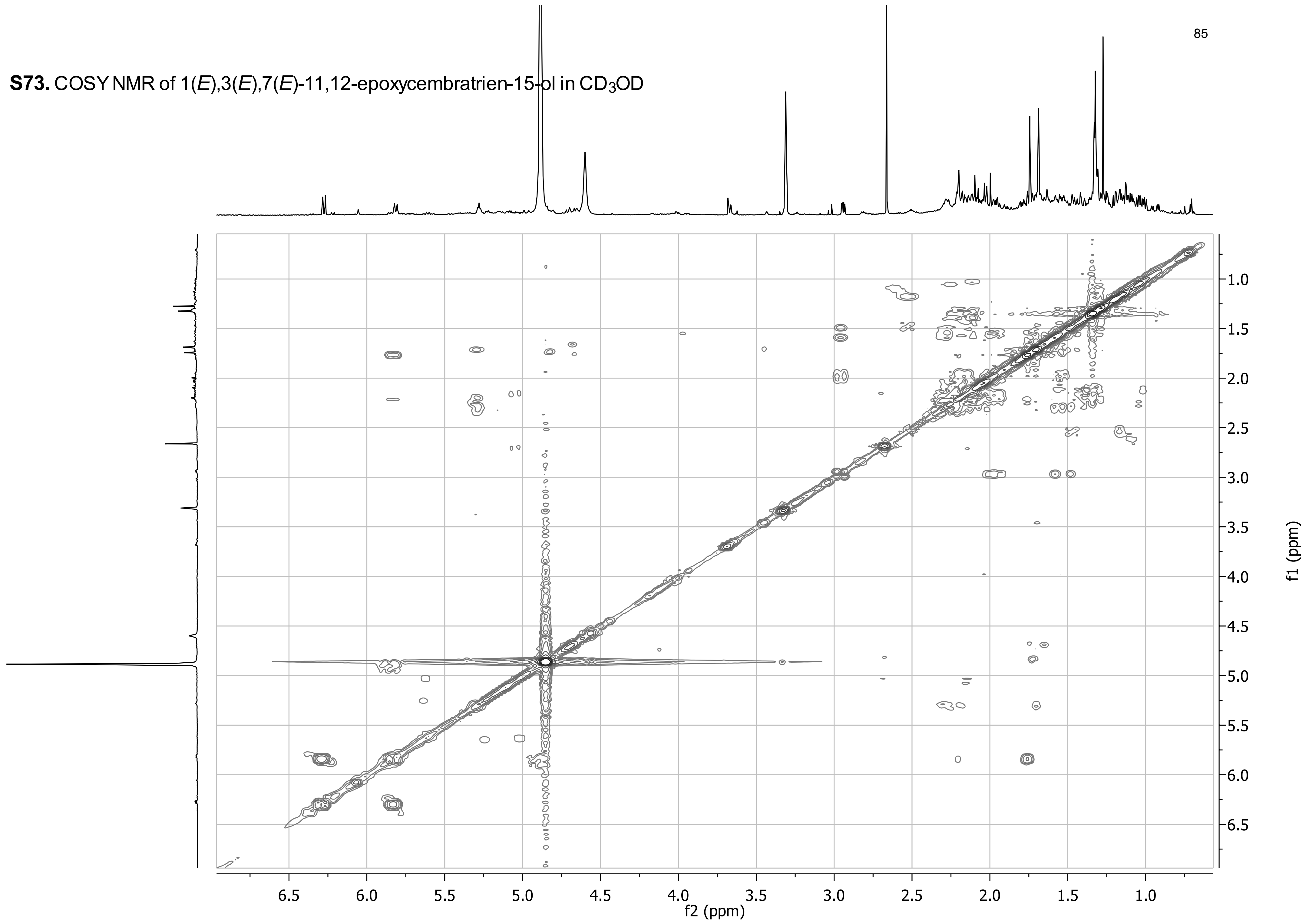

S74. HSQC NMR of 1(*E*),3(*E*),7(*E*)-11,12-epoxycembratrien-15-ol in CD_3OD

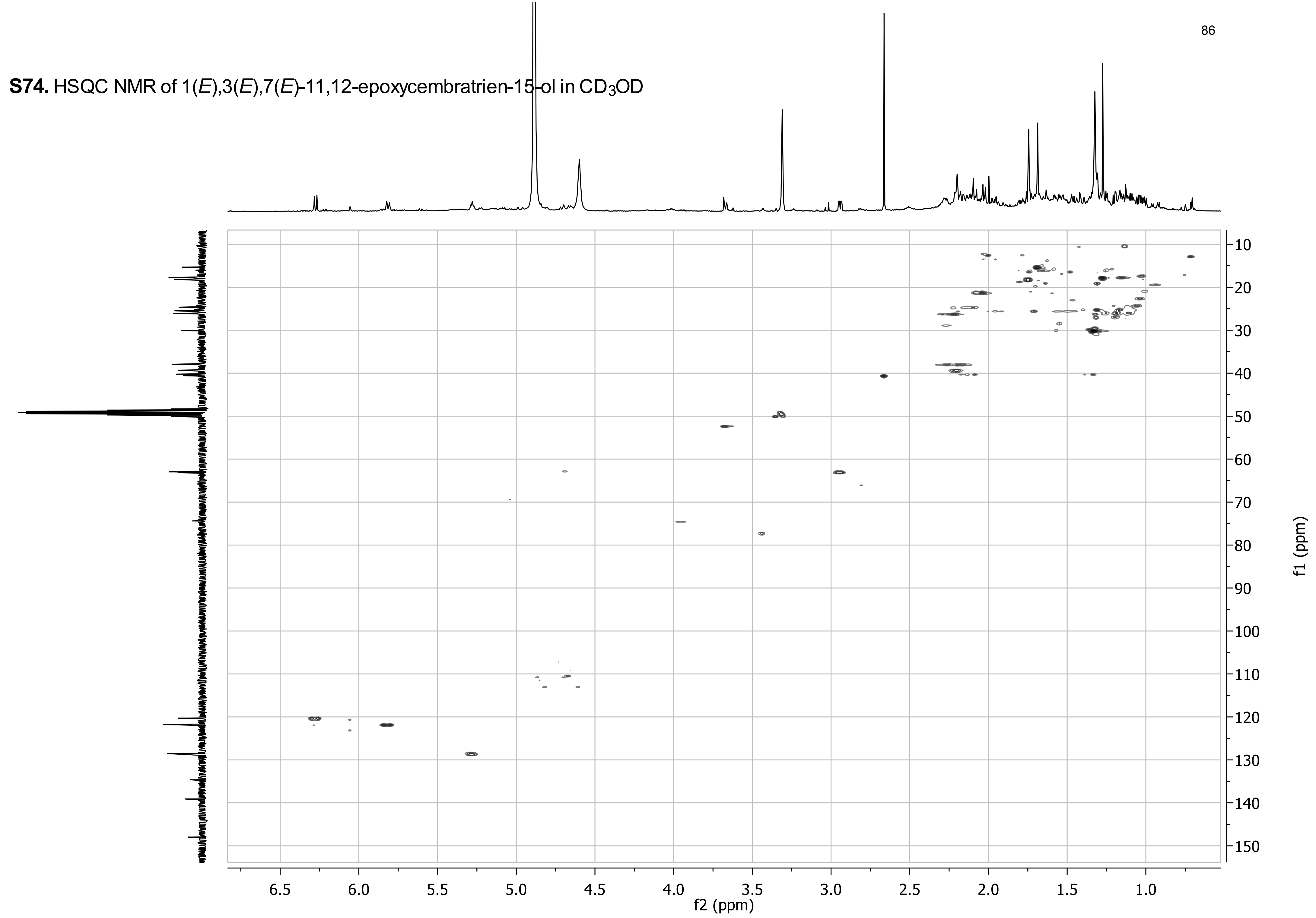

S75. HMBC NMR of 1(*E*),3(*E*),7(*E*)-11,12-epoxycembratrien-15-ol in CD_3OD

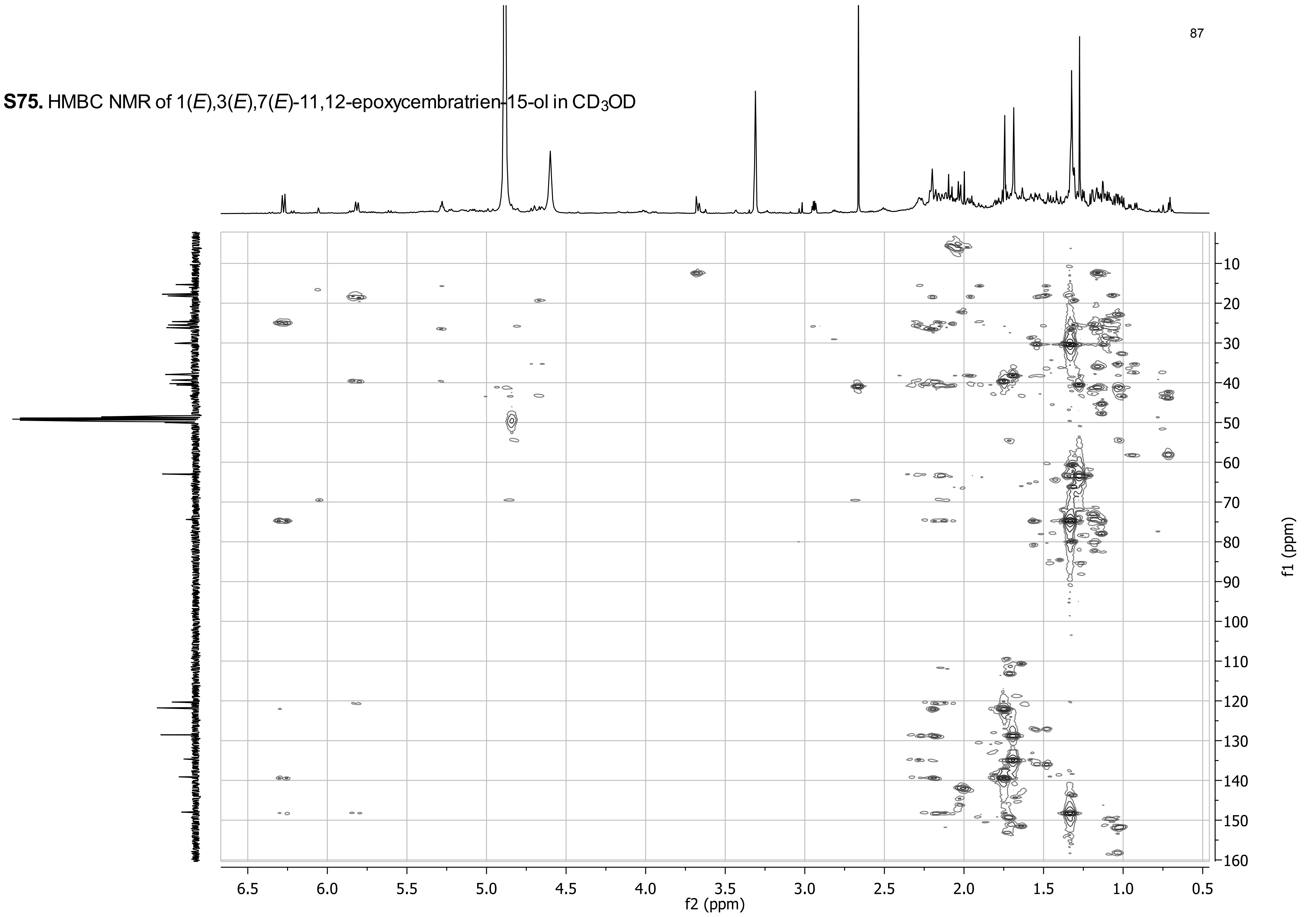

Table S8. ^{1}H and ^{13}C NMR data (300 MHz and 75 MHz, CD_3OD) for 1(*E*),3(*E*),7(*E*)-11,12-epoxycembratrien-15-ol.

No.	^{13}C δ (m)	^{1}H δ (m, J Hz)	COSY	gHMBC
1	147.8 (s)			
2	120.1(d)	6.27 (1H, d, 10.1)	H-3	C-1, C-3, C-4, C-14, C-15
3	121.6 (d)	5.81 (1H, d, 10.1)	H-2, H-18	C-1, C-2, C-5, C-18
4	138.9 (s)			
5	39.2 (t)	2.20 (2H, m)	H_a-6	C-3, C-4, C-6, C-18
6	26.0 (t)	2.32 (1H, m)	H_b-6, H-5	C-4, C-5, C-7, C-8
		2.20 (1H, m)	H_a-6	C-4, C-5, C-7, C-8
7	128.4 (d)	5.28 (1H, brt, 5.7)	H_a-6, H_b-6, H_a-9, H_3-19	C-5, C-9, C-19
8	134.5 (s)			
9	37.8 (t)	2.26 (1H, m)	H-7, H_b-9, H_b-10	C-7, C-8, C-10, C-11, C-19
		2.21 (1H, m)	H_a-9	
10	25.3 (t)	1.96 (1H, m)	H_b-9, H_b-10, H-11	C-9, C-13, C-20
		1.53 (1H, m)	H_a-9, H_a-10, H-11	C-11
11	62.8 (d)	2.94 (1H, dd, 3.9, 8.8)	H_a-10, H_b-10	C-10
12	63.0 (s)			
13	40.0 (t)	2.11 (1H, m)	H_b-13	C-1, C-2, C-11, C-12, C-13, C-15
		1.35 (1H, m)	H_a-13, H-14	C-11, C-12, C-14
14	24.5 (t)	2.13(2H, m)	H_b-13	C-1, C-2, C-12, C-13, C-15
15	74.2(s)			
16	29.9 (q)	1.32 (3H, s)		C-1, C-14, C-15
17	30.0 (q)	1.32 (3H, s)		C-1, C-14, C-15
18	18.0 (q)	1.74 (3H, brs)	H-3	C-3, C-4, C-5
19	15.2(q)	1.69 (3H, brs)	H-7	C-7, C-8, C-9
20	17.6 (q)	1.27 (3H, s)		C-11, C-12, C-13
